# Dramatic biases in terrestrial nitrogen fixation in Earth System Models revealed by natural isotope signatures

**DOI:** 10.1093/nsr/nwaf459

**Published:** 2025-10-28

**Authors:** Maoyuan Feng, Shushi Peng, Philippe Ciais, Daniel S Goll, Benjamin Z Houlton, Ying-Ping Wang, Yilong Wang, Pan Liu, Joshua B Fisher, Pierre Regnier

**Affiliations:** Sino-French Institute for Earth System Science, College of Urban and Environmental Sciences, and Laboratory for Earth Surface Processes, Peking University, Beijing 100871, China; Institute of Carbon Neutrality, Peking University, Beijing 100871, China; Department of Geoscience, Environment & Society—BGEOSYS, Université Libre de Bruxelles, Brussels 1050, Belgium; Sino-French Institute for Earth System Science, College of Urban and Environmental Sciences, and Laboratory for Earth Surface Processes, Peking University, Beijing 100871, China; Institute of Carbon Neutrality, Peking University, Beijing 100871, China; Laboratoire des Sciences du Climat et de l’Environnement, LSCE/IPSL, CEA-CNRS-UVSQ, Université Paris-Saclay, Gif-sur-Yvette 91191, France; The Cyprus Institute, Nicosia 2121, Cyprus; Laboratoire des Sciences du Climat et de l’Environnement, LSCE/IPSL, CEA-CNRS-UVSQ, Université Paris-Saclay, Gif-sur-Yvette 91191, France; Department of Ecology and Evolutionary Biology and Department of Global Development, Cornell University, Ithaca, NY 14853, USA; CSIRO Environment, Clayton South 3169, Australia; State Key Laboratory of Tibetan Plateau Earth System, Environment and Resources (TPESER), Institute of Tibetan Plateau Research, Chinese Academy of Sciences, Beijing 100101, China; State Key Laboratory of Water Resources Engineering and Management, Wuhan University, Wuhan 430072, China; Schmid College of Science and Technology, Chapman University, Orange, CA 92866, USA; Department of Geoscience, Environment & Society—BGEOSYS, Université Libre de Bruxelles, Brussels 1050, Belgium

**Keywords:** biological nitrogen fixation, nitrogen isotope ratios, mycorrhizal fungi, Bayesian approach, machine learning

## Abstract

Biological nitrogen fixation (BNF) is the primary input of new reactive nitrogen to natural terrestrial ecosystems. However, this flux is poorly constrained due to its unclear drivers and associated control mechanisms. Here, we extend the existing theory of nitrogen (N) isotope mass balance to estimate BNF rates and then use a Bayesian approach to constrain the BNF rates in natural terrestrial ecosystems by using measurements of natural N-isotope ratios (δ^15^N) in plants (δ_P_) and soil (δ_S_). Together with pairwise δ_P_ and δ_S_ measurements from 18 forest sites covering diverse climates and thousands of δ_P_ and δ_S_ observations worldwide, we show that the spatial distribution of the fraction of symbiotic BNF relative to the total external N acquisition by plants (*f*_BNFs_) is primarily controlled by temperature (29%) and mycorrhizal fungi (14%), with colder climate and higher ectomycorrhizal fungi abundance leading to a lower *f*_BNFs_. We find a large discrepancy between the spatial distributions of isotope-based BNF and those simulated by using Earth System Models (ESMs) in the Sixth Phase of the Coupled Model Intercomparison Project (CMIP6). Moreover, we constrain the global total BNF from natural terrestrial ecosystems as 78.2–89.8 Tg N yr^−1^, suggesting a ≥18% underestimation of the global BNF in CMIP6 models. In addition to the temperature dependence found in previous laboratory studies, our isotope-based study suggests a competitive relationship between BNF and mycorrhizal N uptake as another important control mechanism. This complex interplay remains unresolved in ESMs and has the potential to improve BNF simulations in the next phase of CMIP.

## Teaser

This study maps global natural biological nitrogen fixation by combining a Bayesian approach and thousands of nitrogen isotope data from plants and soils worldwide based on the nitrogen isotope mass balance. It is found that the current CMIP6 Earth System Models often fail to accurately predict how nitrogen is naturally fixed on land.

## INTRODUCTION

The biological nitrogen fixation (BNF) of atmospheric dinitrogen, via symbiotic (by root nodules hosting N-fixing bacteria (N-fixers)) and free-living (taken by soil microbes) pathways [1,2], is the largest reactive nitrogen (N) input into natural terrestrial ecosystems [3,4]. Although recognized as a key N supply sustaining terrestrial carbon sequestration under elevated CO_2_ [5,6], the size, drivers and main control mechanisms of BNF remain unclear. Recent published estimates of the global natural BNF range from 100–290 Tg N yr^−1^ (model-data based upscaling) [7] to 40–100 Tg N yr^−1^ (N isotopes) [6] and to 52–130 Tg N yr^−1^ (meta-analysis of site observations) [2]. This large variation is primarily due to (i) the limited number of *in situ* BNF observations [2]; (ii) the uncertainty in scaling up BNF fluxes from local site measurements to larger spatial and temporal scales [1,8]; and (iii) the complex and partly unresolved interplay between N_2_-fixing microbes and plants, which provide the energy required for nitrogenase-mediated BNF reactions, either in the form of symbioses or via free-living bacteria feeding on dead plant material [9]. To overcome these limitations, we constrain BNF in natural terrestrial ecosystems with paired observations of N isotopes (^15^N/^14^N or δ^15^N) in soils and plants (δ_S_ and δ_P_) by using a Bayesian approach, which is rooted

in the isotope mass balance principle and also accounts for the complex interplay between microbes and plants.

**Figure 1.**
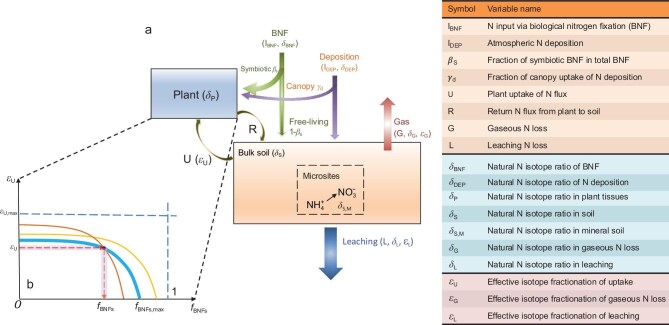
Theoretical basis for inferring BNF from natural N-isotope ratios (δ^15^N, δ = (α_sample_/α_standard_ – 1) × 1000) of plant and soil. (a) Box model for the mass balance of N and ^15^N in a plant–soil system, extended from Houlton *et al*. [10,11]. The symbols and names of involved variables are listed in the accompanied table. (b) Theoretical negative relationship between the fraction of symbiotic BNF in vegetation-external N demand (*f*_BNFs_) and the isotope fractionation of plant uptake (*ε*_U_). *f*_BNFs, max_ and *ε*_U, max_ are potential maximum values of *f*_BNFs_ and *ε*_U_, respectively, given a pair of δ_P_ and δ_S_ measurements.

Nitrogen isotope observations (δ^15^N) have been used to infer key biological processes in ecosystem N cycling based on the kinetic discrimination of ^15^N over ^14^N [10–16]. Soil δ^15^N observations were used to partition N losses into gaseous versus leaching processes [10–12] and to provide earlier estimates of global N fixation rates, yet without spatial or temporal details [6]. Soil and plant δ^15^N observations were together used to evaluate the relative contributions of different N species (nitrate, ammonium and organic N) to plant-used N in soils [16]. The difference in δ^15^N between plants and soil (δ_P_ − δ_S_) is positively correlated with soil N mineralization rates that control the recycling of N within ecosystems and thus indicates plant N availability [14,17,18]. Here, to leverage the isotope signature of δ_P_ − δ_S_ to infer the BNF rates, we extended the existing theory of N-isotope mass balance [10,11] by adding previously omitted fluxes of symbiotic BNF and the canopy uptake of N deposition (Fig. 1a and Text S1). Moreover, we separated the observed δ_P_ − δ_S_ into the contributions of plant uptake, which fractionates the heavier isotopes, versus new input by symbiotic fixation. Symbiotic N fixation does not fractionate N isotopes and brings new nitrogen with a δ^15^N signature close to zero, which dilutes ^15^N into the plant N stock. Specifically, to explicitly represent the different N-acquisition pathways of vegetation, we defined two key variables—*f*_BNFs_ and *f*_DEPd_, the fractions of symbiotic BNF and canopy uptake of N deposition, respectively—composing the total flux of N acquired by plants (see definitions by Equation (2)). Based on isotope mass balance theory, we derived a theoretical negative relationship between the isotope fractionation of plant N uptake (*ε*_U_) and the symbiotic fixation fraction *f*_BNFs_ (Equations (3–4), Fig. 1b and Figs S1–S4), which allows us to constrain *f*_BNFs_ from δ_S_ and δ_P_ observations by using a Bayesian approach, given the prior knowledge of *ε*_U_ and *f*_DEPd_ (see ‘Materials and methods’).

In the Bayesian cost function that is minimized to fit the δ_S_ and δ_P_ observations and solve for *f*_BNFs_, the prior values of *ε*_U_ and *f*_DEPd_ are assumed to be Gaussian-distributed and are optimized together (see ‘Materials and methods’). Combining a unique dataset (*n* = 30) of pairwise collocated *in situ* measurements of δ_P_ and δ_S_ from 18 forests growing under diverse climates [19,20] with a much larger number (>5000) of global δ_P_ and δ_S_ samples in which the plant and soil δ^15^N measurements do not come from the same site [18,21,22], we identify the key climatic and biological drivers of the fraction of symbiotic BNF to total plant N acquisition by using explainable machine-learning tools and produce global maps of BNF. This isotope-based knowledge is finally used to benchmark the recent Earth System Models (ESMs) in the Sixth Phase of Coupled Model Intercomparison Project (CMIP6), which include a process-based representation of BNF and simulate carbon–nitrogen interactions.

## RESULTS AND DISCUSSION

### Theoretical basis of isotope-based partition of vegetation N acquisition

We extended the theoretical isotope mass balance model of the plant–soil system of Houlton *et al.* [10,11] by adding previously omitted fluxes of symbiotic BNF and the canopy uptake of atmospheric N deposition (Fig. 1a). Although rock N weathering is a globally or regionally important flux in terrestrial ecosystems that accounts for 11%–20% of new N supply [23], this input occurs mainly in areas of high relief and tectonic uplift that are underlain by N-enriched sedimentary rocks [23] and the fraction of this input that goes into the vegetation N demand, from 1.2 [24] to 2.7 Pg N yr^−1^ [25], is relatively small, especially for the environments in which the BNF is high. Thus, the isotope effect of this N input is negligible and was not considered in our model. Our extended theoretical model to estimate BNF requires two assumptions: (i) that the ecosystem gains and losses of N and ^15^N are in balance or close to steady state; and (ii) that the N resorption and storage in plant organs prior to leaf and root senescence do not substantially alter the plant N-isotope composition (δ_P_). With these two assumptions, δ_P_ can be expressed by using the flux-weighted sum of the incoming δ^15^N from plant N uptake, symbiotic BNF and the direct canopy uptake of N deposition (Text S1), leading to:

**Figure:**
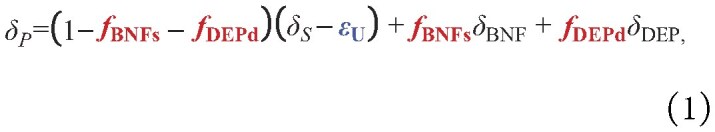


where *ε*_U_ is the isotope fractionation of plant N uptake that depends on direct root versus mycorrhiza-mediated uptake [13,15,17] (key parameter, in blue). δ_BNF_ and δ_DEP_ are the respective δ^15^N signatures of BNF and deposition. The *ε*_U_ value is higher for plants associated with ectomycorrhizae (ECM) than for those with arbuscular mycorrhiza (AM), while the direct root uptake of soil mineral N has a lower fractionation than mycorrhizal uptake [17]. The two unknowns in Equation (1) are *f*_BNFs_ and *f*_DEPd_ (in red)—the fractions of symbiotic BNF and the canopy uptake of N deposition in the total external N demand of plants that is defined as the sum of symbiotic BNF, the canopy uptake of N deposition and the root uptake of soil mineral N:


(2a) \begin{eqnarray*}
{f}_{{\mathrm{BNFs}}} = \displaystyle\frac{{{\beta }_{\mathrm{s}}{I}_{{\mathrm{BNF}}}}}{{{\beta }_{\mathrm{s}}{I}_{{\mathrm{BNF}}} + {\gamma }_{\mathrm{d}}{I}_{{\mathrm{DEP}}} + {\mathrm{ }}U}},
\end{eqnarray*}

(2b) \begin{eqnarray*}
{f}_{{\mathrm{DEPd}}} = \displaystyle\frac{{{\gamma }_{\mathrm{d}}{I}_{{\mathrm{DEP}}}}}{{{\beta }_{\mathrm{s}}{I}_{{\mathrm{BNF}}} + {\gamma }_{\mathrm{d}}{I}_{{\mathrm{DEP}}} + U}},
\end{eqnarray*}

where *I*_BNF_ is the total BNF flux (via symbiotic and free-living pathways) and *β*_s_ is the fraction of symbiotic fixation in *I*_BNF_; *I*_DEP_ is the total N deposition to the vegetation canopy and soil, *γ*_d_ is the fraction of canopy uptake in *I*_DEP_ and *U* is the total root uptake of soil mineral N from direct root uptake and the AM and ECM pathways. The term *β*_s_*I*_BNF_ + *γ*_d_*I*_DEP_ + *U* in the denominator of Equations (2a–2b) represents the vegetation N demand from external sources (here called the vegetation-external N demand) and is equal to the total vegetation N demand minus the internal vegetation-recycled N flux through resorption. Note that the free-living N fixation is excluded here because it is an influx of new N that enters the soil directly and thus is implicitly included in the plant N uptake. However, as some BNF fluxes that enter plants can originate from free-living N-fixers, this assumption may result in an overestimation of the symbiotic BNF in N-limited boreal regions.

For a given level of *f*_DEPd_, solving for the symbiotic fraction *f*_BNFs_ as the unknown term of Equation (1) results in a theoretical relationship (Equation (3)) that links *f*_BNF__s_ to δ_P_ − δ_S_:

**Figure:**
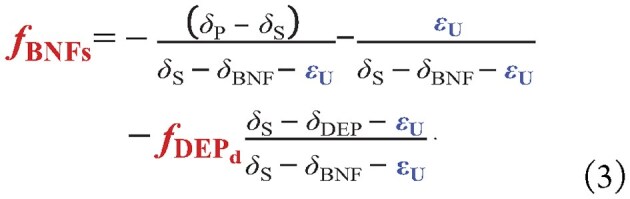


In most cases, δ_S_ − δ_BNF_ − *ε*_U_ > 0 and δ_P_ − δ_S_ < 0 (Figs S1–S4); thus, the first term on the right-hand side of Equation (3) is positive. Since *ε*_U_ could vary strongly with δ_P_ − δ_S_ and *f*_BNFs_ as in the real world, a more negative δ_P_ − δ_S_ indicates a smaller *f*_BNFs_ (when the absolute of derivative of eU over *f*_BNFs_ is large), corresponding to an ecosystem that is more prone to acquiring N by taking up soil mineral N rather than by performing symbiotic BNF [14,17,18]. The value of δ_P_ − δ_S_ is therefore related to the ecosystem N limitation/availability, as noted by Craine *et al.* [14]. From Equation (3), the isotope fractionation of plant uptake *ε*_U_ can be isolated and is equal to:

**Figure:**
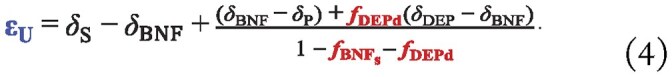


As ${\mathrm{(}}{\delta }_{{\mathrm{BNF}}\ {\mathrm{ }}}\mathfrak{-} {\mathrm{\ }}{\delta }_{\mathrm{P}}{\mathrm{)\ + \ }}{f}_{{\mathrm{DEPd}}}{\mathrm{(}}{\delta }_{{\mathrm{DEP}}}{\mathrm{\ }}\mathfrak{-}{\mathrm{\ }}{\delta }_{{\mathrm{BNF}}}{\mathrm{) < 0}}$ holds for almost all cases (Figs S1–S4), a higher *f*_BNFs_ corresponds to a lower *ε*_U_, i.e. Equation (4) shows a theoretical negative relationship between *f*_BNFs_ and *ε*_U_ (Fig. 1b, Figs S1–S4 and Text S1). This implies that an ecosystem with a higher *f*_BNF__s_ has plants that are more likely to take up the remaining N that is not delivered by BNF from direct root uptake and/or the AM pathway (lower *ε*_U_) rather than from the ECM pathway (higher *ε*_U_). Note that all these theoretical analyses were conducted at an ecosystem scale (composed of plants of different species) in which N-fixing nodules and mycorrhizal fungi could coexist, rather than at the level of individual plant species in which the microbial associations can be exclusive. Overall, the theoretical relationships set by Equations (1), (3) and (4) allow us to constrain *f*_BNF__s_ from δ_S_ and δ_P_ observations, if we include some prior knowledge of *ε*_U_ and *f*_DEPd_. Based on these theoretical relationships, we have thus developed a Bayesian approach to infer *f*_BNF__s_ from pairwise plant and soil isotope observations (δ_S_ and δ_P_) (see ‘Materials and methods’).

### Temperature dependence of isotope-based *f*_BNFs_

**Figure 2.**
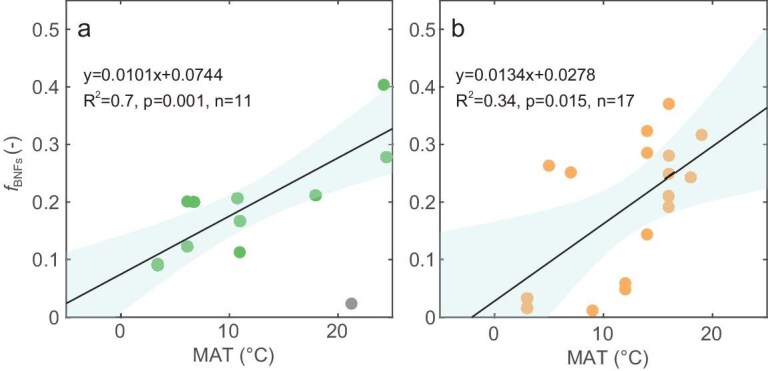
Temperature dependence of the isotope-based fraction of symbiotic BNF (*f*_BNFs_) in vegetation-external nitrogen (N) demand in natural forest ecosystems. (a) Isotope-based *f*_BNFs_ derived from paired plant and soil δ^15^N observations collected in 12 natural forests across tropical, subtropical, warm-temperate and cool-temperate climate regions (data from Gurmesa *et al.* [20]). (b) Isotope-based *f*_BNFs_ derived with paired plant and soil δ^15^N observations from climosequences at six forests compiled by Amundson *et al.* [19]. The gray point in (a) represents the forest site at Xishuangbanna, China, which was excluded from regression due to its exceptionally low fBNFs despite at a high temperature. The solid lines and the filled areas are the best regression lines and the 95% confidence intervals, respectively. Note that the theoretical model is only valid when δ^15^N of plants (δ_P_) values are higher than those of BNF (δ_BNF_), such that the points with δ_P_ ≤ δ_BNF_ were excluded from the regression.

Pairwise *in situ* measurements of δ_S_ and δ*_P_* from 12 natural forests in tropical, subtropical, warm-temperate and cool-temperate climate regions in China [20] (Table S1) were used to estimate the isotope-based *f*_BNF__s_ (see ‘Materials and methods’). We found a positive linear relationship between *f*_BNF__s_ and the mean annual temperature across the sampling locations (Fig. 2a; *R*^2^ = 0.70, *P* = 0.002). The fact that a warmer temperature favors higher *f*_BNF__s_ is consistent with the analysis from the climosequence data in six natural forests compiled by Amundson *et al.* [19] (Fig. 2b; *R*^2^ = 0.34, *P* = 0.015; Table S2). This positive effect of temperature on the symbiotic fraction of BNF is also supported by the temperature dependency of the rates of symbiotic N_2_-fixing reactions measured in the lab (between 0 and 20°C) found by Bytnerowicz *et al.* [26] and indirectly by the larger abundance of N_2_-fixing trees in warmer climates interpreted by Houlton *et al.* [27]. Note that the N-fixing trees are not necessarily fixing N, depending on the availability of other N sources at a given point in time and space, which might result in a lower carbon cost. In our dataset, one forest location (Xishuangbanna, China) has an exceptionally low *f*_BNF__s_ despite growing at a high temperature (21°C) (Fig. 2a, gray point), but we could not identify a peak (or optimal) temperature above which *f*_BNF__s_ declines [26,27]. Considering that *f*_BNF__s_ essentially reflects the strategies of plants interacting with microbial communities to acquire N, the temperature dependence of *f*_BNF__s_ also reflects the climatic control on major symbioses (N-fixers and ECM and AM fungi), in line with the global analysis by Steidinger *et al.* [8]. We acknowledge that, although the sites with paired measurements cover a wide range of climate conditions, the sites and data records are limited and not evenly distributed across regions, thereby failing to cover all critical regions to fully capture the spatial heterogeneity. Thus, the robustness of our analysis could be improved by expanding field campaigns in data-scarce regions and including more data from sites of diverse vegetation species (e.g. forests, grasses) in the near future.

**Figure 3.**
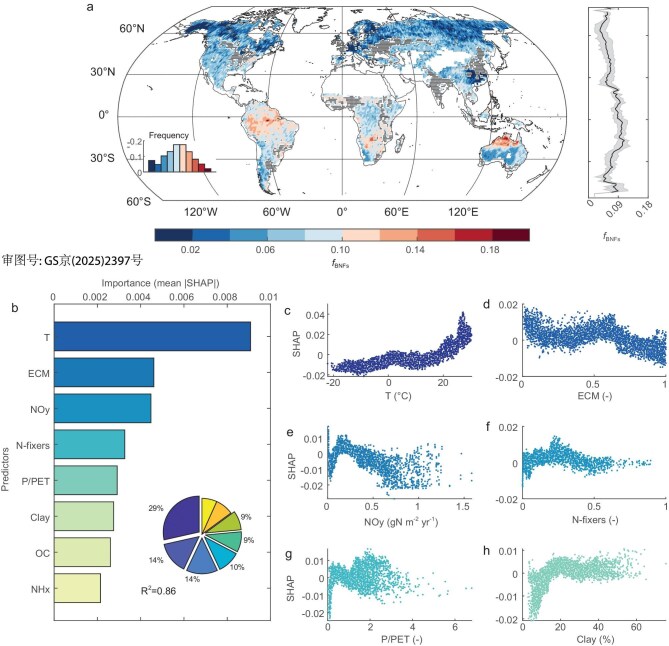
Key roles of temperature and soil fungal abundance in controlling global distribution of isotope-based fraction of symbiotic biological nitrogen fixation in total BNF (*f*_BNFs_). (a) Global distribution of isotope-based *f*_BNFs_ derived from the Bayesian approach. (b) Importance of all selected predictors, including temperature (*T*), ECM, NO*_y_* deposition, N-fixers, aridity index (P/PET), clay fraction (Clay), soil organic carbon content (OC) and NH*_x_* deposition. The pie chart in the bottom right corner indicates the percentage contributions of these predictors. (c–h) Partial dependence plots for the top six predictors. The partial dependence is evaluated by using the SHAP value, which indicates the marginal effect of including an additional variable in predicting *f*_BNFs_.

Evidence that *f*_BNF__s_ increases with temperature is further corroborated by our global map of *f*_BNF__s_ (1° × 1°; Fig. 3a) derived by upscaling thousands of observations of δ_S_ and δ_P_ (Texts S2–S4). Specifically, global maps of δ_S_, δ_P_ and their difference (δ_P_ – δ_S_) were upscaled from 38 646 foliar δ^15^N and 5887 soil δ^15^N measurements [18,21,22], which cover large portions of the land surface, by using random forest (RF) models (see ‘Materials and methods’; Text S2, Figs S5–S10 and Table S3). The global map of *f*_BNFs_ derived from the Bayesian optimization (see ‘Materials and methods’) shows that 6% [5–15] of the vegetation total external N demand is satisfied by symbiotic BNF (Fig. 3a and Fig. S11a). Symbiotic BNF represents 6% [5–18] of the N uptake from soil—slightly lower than the estimate of 9% simulated by a model based on N-acquisition-costs theory [22]. Spatially, *f*_BNF__s_ values decrease from 11% in tropical regions to 3% in boreal regions (Fig. 3a), consistently with the latitudinal variations reported by previous studies [2,7,22]. Among the eight factors involved in predicting *f*_BNF__s_ at a global scale (see ‘Materials and methods’), the temperature was the dominant factor, explaining 29% of the spatial variations in *f*_BNFs_ (Fig. 3b). The partial dependence plot indicates a monotonically increasing relationship between the isotope-based *f*_BNFs_ and temperature, with no decline at high temperatures (Fig. 3c).

### The control of soil fungal abundance on global *f*_BNFs_

In addition to temperature, we identified the abundance of mycorrhizal fungi [8] as another key factor for predicting *f*_BNF__s_, with the natural abundance of ECM explaining 14% of the spatial variations in *f*_BNF__s_ (Fig. 3b). Note that abundances of ECM and AM are highly correlated (with *R* = –0.96; see ‘Materials and methods’) and thus the latter were removed from the analysis. The partial dependence analysis suggests that *f*_BNF__s_ decreases with ECM abundance (Fig. 3d) and the effects of mycorrhizal fungi are stronger for higher temperatures (Figs S12 and S13). This implies that lower ECM (or higher AM) abundance and warmer temperatures together explain the higher values of *f*_BNFs_ found in warmer biomes such as tropical forests (Fig. S13), in line with the study of Houlton *et al.* [27]. As this finding can be supported by the analysis at a global scale (Fig. S12) and that from different latitudinal bands (Fig. S13), our global-scale results are robust despite the uneven distribution of data in different latitudinal regions. The controlling effects of ECM (or AM) on *f*_BNFs_ as well as their synergetic effect with temperature are the result of the competition and coevolution of vegetation N acquisition through mycorrhizal N uptake, bacterial N fixation and bare-root uptake pathways [9,25]. In particular, mycorrhizal N uptake from the ECM and AM pathways shows opposite latitudinal gradients (AM pathway dominates in tropics while ECM dominates in boreal regions (Fig. S14)) [28], which reflects the spatial distributions of natural abundances of ECM and AM fungi [8] and results from past coevolutions of vegetation, microbes and environmental conditions [9] as well as the acclimation of vegetation–symbiont associations with environmental N availability. These competitive and co-evolutive relationships are also recorded by isotopic signatures, which set the basis of our isotope-based Bayesian approach. Specifically, N-uptake pathways through ECM and AM fungi and bare roots without mycorrhizae have decreasing isotope-fractionation factors, i.e. *ε*_ECM_ > *ε*_AM_ > *ε*_root_. Thus, a higher δ_S_ − δ_P_ results from a higher fraction of N acquired from the pathway with the largest isotope fractionation (i.e. the ECM pathway), as revealed by our theoretical derivation (Equations (3–4)). These features explain why mycorrhizal fungi abundance is another key control factor of the large-scale spatial distribution of *f*_BNFs_ [8,9,25,28].

We found that the relative abundance of N-fixers [8] explains 10% of the spatial variations in isotope-based *f*_BNFs_ (Fig. 3f), consistent with the knowledge that the systems with a higher abundance N-fixers promote the N-acquisition pathway through symbiotic BNF [8,26,27]. In addition, NO*_y_* deposition, aridity index (the ratio of precipitation over potential evapotranspiration (P/PET)) and clay content also play important roles in predicting isotope-based *f*_BNF__s_ (Fig. 3e, g and h) (with 14%, 9% and 9% of the spatial variability in *f*_BNFs_ explained by these factors, respectively). In natural terrestrial ecosystems, with NO*_y_* deposition increasing from a low level, the N-limited condition could be progressively alleviated, promoting plant growth and increasing the carbon supply for symbiotic BNF (as revealed by increases in *f*_BNF__s_; Fig. 3d). As NO*_y_* deposition further increases, soil N follows the same pattern and becomes saturated, and plants reduce the investment in fixing N with N-fixers, leading to a decrease in *f*_BNF__s_ (Fig. 3d). The fraction *f*_BNFs_ tends to peak under mesic conditions, as adequate soil moisture supports photosynthesis, nodule formation and microbial metabolism, whereas either drought (low P/PET) or waterlogging (high P/PET) could inhibit the photosynthesis and N demand, thus limiting C investments in nodules. The soil texture (represented by the clay fraction) is also important for symbiotic fixation because it is closely related to the water retention, phosphorus availability, as well as the mineralization in the soil. Increasing the fraction of clay in soil will increase the water-holding capacity and phosphorus availability, but will reduce the N mineralization from organic matters and also reduce the accessibility of plant to mineral N (NH_4_^+^) due to its strong adsorption. Thus, with an increase in clay, the N fixation becomes more cost-efficient but excess clay may lead to waterlogging or poor aeration that suppresses the N fixation. Similarly to mycorrhizal fungi, the controlling effects of these drivers on *f*_BNF__s_ also reflect the coevolutions of vegetation, microbes and environmental N availability [9].

### Large discrepancy between the spatial distributions of isotope-based BNF and CMIP6 models

Based on our global map of isotope-based *f*_BNFs_, we estimated the symbiotic BNF as the product of *f*_BNFs_ by the vegetation-external N demand. The latter was constrained from datasets of the growth of plant tissues (leaf, wood and root; represented by the gross primary production (GPP) and its allocation to tissues) and their respective C:N ratios, as well as N-resorption efficiencies (NREs) [25] (Text S4 and Figs S15 and S16). As the GPP primarily sets the vegetation N demand, we used three different GPP products (MODerate Resolution Imaging Spectroradiometer (MODIS)-GPP [29], Keenan *et al.* [30] and Jung *et al.* [31]) and obtained three similar global patterns of symbiotic BNF (Fig. S17). The average of these three maps provides a global mean rate of symbiotic BNF equal to 0.59 (0.52–0.65) g N m^−2^ yr^−1^ (Fig. S18). The latitudinal mean of the symbiotic BNF rate decreases from 1.45 g N m^−2^ yr^−1^ in tropical regions to approximately zero (0.07 g N m^−2^ yr^−1^) in boreal regions, close to the values estimated by using meta-analysis [2].

With the fraction of symbiotic fixation in total BNF (*β*_s_) taken from observations [2] (Fig. 1, ‘Materials and methods’ and Fig. S15b), we further infer the fraction of total BNF in vegetation-external N demands (${f}_{{\mathrm{BNF}}_{\mathrm{T}}}$) as ${f}_{{\mathrm{BNF}}_{\mathrm{T}}} = {\mathrm{\ }}f_{\mathrm{BNFs}}$/*β*_S_ (Fig. S11c and d). Then, we estimate the total BNF as the product of ${f}_{{\mathrm{BNF}}_{\mathrm{T}}}$ by the vegetation-external N demand, noting that uncertainties in *β*_S_ propagate in the BNF assessments. The global map of total BNF has a spatial pattern which follows that of symbiotic fixation and leads to a global rate of 0.98 (0.88–1.08) g N m^−2^ yr^−1^ (Figs S18 and S19). Compared with previous studies [2,5,7], our BNF rates are slightly higher in tropical regions and lower in boreal regions (Fig. S20).

**Figure 4.**
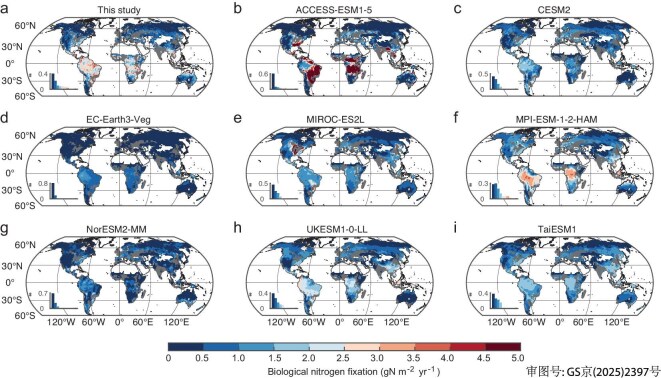
Large discrepancy between the isotope-based global map of BNF and those simulated by CMIP6 ESMs. (a) Isotope-based BNF estimate; (b–i) BNFs simulated by using ESMs in CMIP6. To derive the isotope-based BNF, the fraction of symbiotic fixation in total BNF (*β*_S_) is estimated from Davies-Barnard and Friedlingstein [2]. The areas of managed croplands and pastures were excluded in our analysis and are represented by gray regions.

Despite all the CMIP6 models featuring decreasing patterns of total BNF from tropical to boreal regions, similarly to our analysis, we found large discrepancies between their simulated northward gradients of BNF and our isotope-based estimate (Fig. 4). Among the eight ESMs representative of different groups of models participating in the CMIP6 ensemble runs (Tables S4 and S5), only MPI-ESM-1–2-HAM can produce a spatial pattern of BNF that is close to our isotope-based map, while the other models have latitudinal gradients that are either too large (ACCESS-ESM1-5) or too flat (CESM2, EC-Earth3-Veg, NorESM2-MM, MIROC-ES2L, UKESM1-0-LL and TaiESM1) (Fig. 4). With the observation-based *β*_s_ [2], we can extract the symbiotic fixation from the simulated total BNF and infer spatial patterns of *f*_BNF__s_ that correspond to those of the CMIP6 models (Fig. S21). We found that, although not explicitly simulated, most CMIP6 models (except ACCESS-ESM1-5) have highly biased *f*_BNF__s_ compared with those derived from the isotope constraints—either underestimated (EC-Earth3-Veg, MPI-ESM-1–2-HAM and NorESM2-MM) or overestimated (MIROC-ES2L and UKESM1-0-LL), or even with opposite latitudinal patterns (CESM2 and TaiESM1).

Most of these highly biased BNF estimates in ESMs can be attributed to the representation of this process as a function of the net primary production (NPP; UKESM1-0-LL) or evapotranspiration (ET; EC-Earth3-Veg, MIROC-ES2L and TaiESM1) (Table S5). As a result, the latitudinal gradients of these BNF estimates essentially reflect those of NPP or ET, as prescribed in the BNF model equations (Table S5). Nevertheless, having incorporated the fractions of plants with N_2_-fixing capability or the abundance of AM, ECM and N-fixers and carbon cost theory in some models (ACCESS-ESM1-5 with resource optimization theory based on carbon costs, CESM2 and NorESM2 with the embedding of the Fixation and Uptake of Nitrogen (FUN) model [22]) fails to help them capture the spatial pattern of isotope-based BNF.

### Underestimation of natural biological fixation of nitrogen in ESMs

The symbiotic and total BNF fluxes for global natural terrestrial ecosystems are estimated at 49.0 and 83.0 Tg N yr^−1^, respectively. These estimates account for uncertainties mainly from GPP products [29–31], C-allocation factors and C:N ratios [32–34], as well as prior values of *f*_DEPd_. In particular, the uncertainty ranges for the symbiotic and total BNF induced by the three different GPP products are 46.5–53.4 and 78.2–89.8 (min.–max.) Tg N yr^−1^, respectively (Table 1 and Table S6). Compared with the C-allocation factors obtained from Bloom *et al.* [32] and the leaf C:N ratios estimated from a satellite-based leaf N-concentration map [33], the set of parameters from Wang *et al.* [34] applied here results in a 19% lower symbiotic BNF and a 17% lower total BNF (see ‘Materials and methods’). Moreover, the isotope-based BNF is also sensitive to *f*_DEPd_ (or equivalently to *f*_DEPd, tot_, the fraction of canopy uptake of N deposition in total vegetation N demand) and we found that a 0.05 decrease in *f*_DEPd, tot_ increases the symbiotic and total BNF by 3.0 and 5.2 Tg yr^−1^, respectively (see ‘Materials and methods’ and Tables S7–S9). In addition, the isotope-based BNF could be biased by the observational and prior uncertainty ranges. Using a simple sensitivity test, we found that the BNF estimates are more sensitive to prior uncertainty ranges than observational ones, the latter leading to a variation in symbiotic and total BNF of <1% for a 50% variation in the observational standard deviation (SD) (Tables S10 and S11). Furthermore, the sensitivity to prior uncertainty is not symmetrical—a 50% decrease in prior SDs decreases the BNF estimates by 9%–10%, while a 50% increase in prior SDs only increases the BNF estimates by ∼1%. Compared with the isotope-based BNF estimate by Vitousek *et al.* [6], our analysis has the advances of (i) adopting an updated soil δ^15^N map with more observations and robust RF models; (ii) incorporating a new isotope signal of the plant δ^15^N map; and (iii) providing spatially resolved BNF estimates. Moreover, we highlight that our study provides a present-day BNF estimate, instead of the preindustrial estimate in Vitousek *et al.* [6].

**Table 1.** Comparison of biome-level BNF estimates in this study with previously reported values (in Tg N year^−1^).

		Terrestrial ecosystem model						Bayes inversion model			
		CSCA-CNP (Peng *et al.* [5])				Barnard and Friedlingstein [2]		Symbiotic BNF		Total BNF	
PFT	Area (M km^2^)	Method A	Method B	Method C	ORCHIDEE-CNP^33^	Symbiotic BNF	Total BNF	Mean	Range (min.–max.)	Mean	Range (min.–max.)
ENF	10.8	4.9	4.5	9.7	15.5	4.1	5.5	4.2	3.6–4.7	6.4	5.6–7.2
EBF	15.9	13.9	13.8	22.3	22.6	8.2	14.9	14.8	14.0–15.8	26.7	25.2–28.7
DNF	3.8	0.3	0.2	2.3	1.3	1.4	1.9	0.8	0.5–1.2	1.2	0.7–1.6
DBF	16.9	11.1	10.8	17.4	10.5	10.2	13.8	11.9	10.8–13.2	20.1	18.1–22.4
Grass	33.6	13.9	12.8	26.3	16.1	21. 6	32.5	17.4	15.6–20.5	28.6	26.0–33.8
Total	81.0	44.1	42.1	78.1	66.0	45.5	68.6	49.0	46.5–53.4	83.0	78.2–89.8

The Bayesian approach provides the means and ranges of symbiotic and total BNF estimates by using three sources of GPP: MODIS [29], Jung *et al*. [30] and Keenan *et al*. [31]. For the CASA-CNP model, Methods A and B are based on resources optimization theory, and Method C is C-limited and based on the NPP; see details in Peng *et al*. [5]. ENF, evergreen needleleaf forest; EBF, evergreen broadleaf forest; DNF, deciduous needleleaf forest; DBF, deciduous broadleaf forest; Grass, grasslands, savannas and woody savannas; PFT, plant functional type. Details of the PFT aggregation rules are presented in Table S18.

The steady-state assumption underpins our calculation of BNF fluxes from plant and soil isotope data [10–12] and we found that neglecting the transient effects leads to slightly lower BNF estimates. Although terrestrial N isotopes had negligible variations during the anthropocene due to the long residence time of soil organic N, the plant δ^15^N has declined significantly compared with preindustrial levels, i.e. by 1.6‰ over a recent 37-year period (1980–2017) [18]. We assessed that such a 1.6‰ reduction in plant δ^15^N translates into increases of 3.5 Tg N yr^−1^ in symbiotic BNF and 8.2 Tg N yr^−1^ in total BNF (see ‘Materials and methods’). Thus, our steady-state BNF estimates should be considered as a conservative (lower) benchmark when comparing them with transient simulations by ESMs (see the following paragraph). To overcome this limitation, our steady-state model would need to be extended to a temporal dynamic framework (e.g. Caldararu *et al.* [35]) simulating variations in δ^15^N signals coupled to C and N cycles, in which the dynamical inversion of *f*_BNFs_ and BNF could be implemented.

Our global mean BNF estimate for natural terrestrial ecosystems (83.0 Tg N yr^−1^) suggests an 18% underestimation in the ensemble mean of 11 CMIP6 models (67.7 Tg N yr^−1^) (Fig. S22). This globally lower simulated value results from a 30% underestimation of BNF in the tropical latitudinal band (30°S–30°N), which is partly offset by a 18% overestimation in the three other latitudinal bands (60°–90°N, 30°–60°N and 90°–30°S; Fig. S22 and Table S12). Moreover, the global-scale underestimation in the ESM ensemble could be even higher if we (i) remove the exceptionally high BNF estimate by ACCESS-ESM1-5 (lowering the ensemble mean by 6.3 Tg N yr^−1^); and (ii) account for the transient effects of a 1.6‰ reduction in plant δ^15^N (increasing the isotope-based estimate by 8.2 Tg N yr^−1^). This underestimation in BNF implies that the N limitation could be exaggerated or the N-recycling efficiency could be highly overestimated in CMIP6 models, especially if we combine the overestimated gaseous N losses revealed by our previous isotope-based research [12].

The underestimated BNF fluxes, combined with the highly biased spatial pattern of BNF, suggest that better predictors over NPP or ET should be selected for N_2_-fixing processes, as revealed by our isotope-based analysis and previous observation-based assessments [2], whereas the integration of the complex plants–microbes interplay still needs proper parameterizations and even optimization. To improve our confidence in future projections of the coupled terrestrial C–N cycles, we thus advocate for an improved representation and parameterization of BNF in ESMs. In particular, our isotope-based study underscores the urgent need to incorporate better-informed descriptions of the temperature dependence and the complex interplay between plants and microbes as key control factors of the BNF process. Specifically, symbiotic BNF could be directly formulated in models as a function of temperature, based on laboratory experiments, as in Bytnerowicz *et al.* [26], or the symbiotic BNF could be simulated as the product of *f*_BNFs_ and the vegetation N demand from external sources, with the former formulated as a multivariate function of the identified leading predictors as in Fig. 3 (that include temperature, ECM abundance, NO*_y_* deposition, N-fixers, etc.) established in our isotope-based study. However, this kind of formulation needs more support from *in situ* measurements from diverse vegetation types, ecosystems and climate conditions. Moreover, the model parameters (e.g. carbon costs) involved in the resource optimization theory (adopted in ACCESS-ESM1-5) and/or FUN model (adopted in CESM2 and NorESM2) could be better constrained with robust isotope products (e.g. global maps of plant and soil δ^15^N).

## MATERIALS AND METHODS

### Bayesian approach for deriving *f*_BNFs_ from δ^15^N signals

With the N-isotope mass balance model shown in Fig. 1a, the plant δ^15^N could be quantified by the flux-weighted sum of the plant N uptake, symbiotic BNF and canopy uptake of N deposition, and their respective isotope signatures (Equation (5); Text S1). By incorporating observation errors of plant and soil δ^15^N, the plant δ^15^N can be represented as follows:


(5) \begin{eqnarray*}
{\delta }_P &=& \left( {{\mathrm{1\ }}\! - \!\!{\mathrm{\ }}{f}_{{\mathrm{BNFs}}} {-}{\mathrm{\ }}{f}_{{\mathrm{DEPd}}}} \right)\left( {{\delta }_S{\mathrm{\ }} {-}{\mathrm{\ }}{\varepsilon }_{\mathrm{U}}} \right) +\! {f}_{{\mathrm{BNFs}}}{\delta }_{{\mathrm{BNF}}}\\
&&+\, {f}_{{\mathrm{DEPd}}}{\delta }_{{\mathrm{DEP}}} + \left( {{\mathrm{1\ }} {-}\!{\mathrm{\ }}{f}_{{\mathrm{BNFs}}} {-}\!{\mathrm{\ }}{f}_{{\mathrm{DEPd}}}} \right){e}_{{{\mathrm{\delta }}}_{\mathrm{S}}} + {e}_{{{\mathrm{\delta }}}_{\mathrm{P}}},\\
\end{eqnarray*}

where ${e}_{{{\mathrm{\delta }}}_{\mathrm{S}}}$ and ${e}_{{{\mathrm{\delta }}}_{\mathrm{P}}}$ are the observation errors of plant and soil δ^15^N, respectively, which were captured by ensembles produced by the RF models; δ_BNF_ and δ_DEPd_ are the δ^15^N signals of BNF and atmospheric N deposition, respectively, after considering isotope fractionations, which were set as constant across the globe [10–12] (Table S13). The δ_BNF_ was reported to be −2.02 ± 2.2‰ [15] and here we adopted a central value of −2.02‰. The δ_DEP_ was reported to be within a range of −3 to 3‰ [36,37] and here we adopted a value of 1.5‰, considering that the observations from ice core indicate a positive value of δ_DEP_ for natural ecosystems [38,39].

We adopted a Bayesian approach to derive the vector of parameter ${\mathrm{\bf m\ = \ }}{[ {{f}_{{\mathrm{BNFs}}}{\mathrm{,\ }}{f}_{{\mathrm{DEPd}}}{\mathrm{,\ }}{\varepsilon }_{\mathrm{U}}} ]}^T$, given the observational constraints ${\mathrm{\bf d\ = \ }}{[ {{{\mathrm{\delta }}}_P{\mathrm{,\ }}{{\mathrm{\delta }}}_S} ]}^T\ $(henceforth *p*(**m**|**d**)) [40,41], i.e.:


(6) \begin{eqnarray*}
p{\mathrm{(\bf m|\bf d)\ }} \propto {\mathrm{\ }}p{\mathrm{(\bf d| \bf m)}}p{\mathrm{(\bf m)}},
\end{eqnarray*}

where *p*(**m**) is the prior probability density function of the model parameters and *p*(**d**|**m**) is the likelihood of **d** with respective to **m**. The posterior model parameters can be optimized by using an ensemble implementation of the Kalman Filter (EnKF) iteratively [40,41] (Texts S3 and S5), with both the observation errors and the parameter uncertainties assumed as Gaussian distributions. Within each grid cell, the model parameter **m** is derived from 10 × 1000 samples (i.e. each time with 1000 samples and repeated 10 times to eliminate the impacts of stochasticity). The global maps of *f*_BNFs_, *f*_DEPd_ and *ε*_U_ were constrained simultaneously by plant and soil δ^15^N. Considering that some physical constraints (e.g. nonnegative conditions) on parameters *f*_BNFs_, *f*_DEPd_ and *ε*_U_ may have hindered the use of EnKF, we first implemented the standard EnKF (unconstrained) iteratively to obtain a stable solution and then adopted the physical constraints to constrain the final global maps of *f*_BNFs_, *f*_DEPd_ and *ε*_U_ (Texts S3 and S5).

### Datasets for deriving global maps of plant and soil δ^15^N

To produce global maps of plant and soil δ^15^N (δ_P_ and δ_S_) and their difference (δ_P_ – δ_S_), we utilized δ^15^N observations from a global foliar δ^15^N dataset that contains 38 646 site-level measurements compiled by Craine *et al.* [18] and a global soil δ^15^N dataset that contains 5887 measurements compiled by Craine *et al.* [21] (*n* = 5609) and Sena‐Souza *et al.* [22] (*n* = 278). The global maps of δ_P_, δ_S_ and δ_P_ – δ_S_ were produced using 16 predictors with gridded fields, including three climate drivers, three natural abundances of microbial symbionts, seven soil properties, GPP, and NH*_x_* and NO*_y_* deposition fluxes (Table S3). The three climate drivers are precipitation (*P*), temperature (*T*) and P/PET. The 0.5° × 0.5° monthly data of *P, T* and PET during the period 1981–2018 were collected from the Climate Research Unit (CRU) Time Series (TS) v4.03 datasets. The 1° × 1° global maps of the abundance of three microbial symbionts (AM, ECM and N-fixers) were collected from Steidinger *et al.* [8]. The 10 km × 10 km global maps of seven soil properties (bulk density, organic carbon (OC), C/N ratio, soil pH and fractions of clay, sand and silt) were collected from the Global Soil Datasets for Earth System Modelling produced by Beijing Normal University (BNU) [42]. This BNU soil dataset provides soil information across eight soil layers (from 0 to 2.3 m) and herein we adopted soil information of the upper four layers (∼30 cm). The 0.5° × 0.5° NO*_y_* and NH*_x_* deposition fluxes (1982–2015) were collected from Tian *et al.* [43]. The GPP were collected from three sources: (i) 10 km × 10 km monthly data (2000–2019) from MODIS [29]; (ii) the 0.5° × 0.5° monthly data (1982–2016) from Keenan *et al.* [30]; and (iii) the 0.5° × 0.5° monthly data (1988–2017) from Jung *et al.* [31]. These three GPP products were used independently in our RF models and Bayesian approach. All the monthly data were averaged to obtain a mean annual value and all these predictors were re-gridded at a resolution of 0.1° × 0.1°.

### RF model for global maps of plant and soil δ^15^N

We first aggregated 38 646 and 5887 site-level measurements of plant and soil δ^15^N (δ_P_ and δ_S_) into 2238 and 933 0.1° × 0.1° grid cells, respectively (locations shown in Fig. S5). Then we collected the 16 predictors in grid cells in which δ_P_ and δ_S_ observations were available. With these gridded δ_P_ and δ_S_ observations, we employed three independent RF models to produce global maps of δ_P_, δ_S_ and δ_P_ − δ_S_ by using a well-established package in Python v3.8.5, RandomForestRegressor (Figs S6–S10 and Text S2). To test the reliability of these three RF models, we also conducted a *K*-fold (*K* = 10) cross-validation and found that withholding 10% of the samples could only slightly degenerate the model performances, i.e. decrease the *R*^2^ by <5% for the model validation (Fig. S7). This indicates that the RF models were robust to predict the global maps of δ_P_, δ_S_ and δ_P_ − δ_S_.

The global map of δ_P_ indicated an area-weighted global mean of 0.8 (0.3–1.3)‰ ((min.–max.) of 95% confidence interval) (Fig. S9a)—slightly higher than the previous estimate (0.4‰) from a linear regression model by Amundson *et al.* [19]. Spatially, δ_P_ decreases between low and high latitudes from 4.3‰ to –1.9‰. Similarly, the global map of δ_S_ gives a global mean of 4.8 (4.4–5.3)‰ (Fig. S9b)—slightly lower than the previous estimate of 5.5‰ [11,19]. Estimates of δ_S_ decrease between the low and the high latitudes from 7.6‰ to 2.4‰. Directly subtracting the map of δ_S_ from that of δ*_P_*, which are subject to individual biases, would have added additional uncertainties to the global map of δ*_P_* – δ_S_ and create spurious patterns. Thus, we directly upscaled the δ_P_ – δ_S_ point measurements into a map by using a separate RF model, resulting in a global mean δ_P_ – δ_S_ of –4.2 (–4.6 to –3.7)‰ (Fig. S9c)—slightly higher than the previous estimate of –5.2‰ [19]. The uncertainties (quantified by SDs) for δ_P_, δ_S_ and δ_P_ − δ_S_ are shown in Fig. S10. The global average of SD of δ_S_ is lower than that of δ_P_, which is lower than that of δ_P_ − δ_S_. Spatially, the latitudinal gradients of all these SDs are minor: the SDs of δ_P_ and δ_P_ − δ_S_ share similar patterns, i.e. with the lowest values in the tropical regions and highest values in the boreal regions, whereas the latitudinal gradient of δ_S_ is opposite to those of δ_P_ and δ_P_ − δ_S_.

### Implementation of Bayesian approach at site level and global scale

For the two representative groups of natural forests [19,20] (12 forests across tropical, subtropical, warm-temperate and cool-temperate climate regions in China [20] and climosequence data in six forests [19]; Tables S1 and S2), the site-level measurements of δ_P_ and δ_S_ were directly used to derive the corresponding *f*_BNFs_ via the proposed Bayesian approach. Considering the lack of observations on *f*_DEPd_ and *ε*_U_ at these natural sites, we adopted a prior value of *f*_DEPd_ as zero and prior *ε*_U_ as 7.35‰ [15] (tested to have minor effects on the results, as they will be optimized) such that the derived *f*_BNFs_ were only constrained by the isotope signals.

For the global analysis, we first re-gridded the 0.1° × 0.1° global δ_P_ and δ_S_ maps upscaled by RF models to a resolution of 1° × 1° and then used them to derive the parameter values for *f*_BNFs_, *f*_DEPd_ and *ε*_U_ (Text S3). For each 1° × 1° grid cell, the prior global map of *ε*_U_ was assumed to be a linear combination of the fractions of N-uptake fluxes from roots, the AM and ECM pathways and their respective fractionation factors (Equation (13) in Text S3 and Fig. S14). The prior map of *f*_DEPd_ was estimated by using the fractions of total vegetation N demand satisfied by the canopy uptake of N deposition (*f*_DEPd, tot_) and the vegetation-internally recycled N (*f*_recycled, tot_), i.e. *f*_DEPd_ = *f*_DEPd, tot_/(1 − *f*_recycled, tot_). As the direct measurements of *f*_DEPd, tot_ are very limited and *f*_DEPd, tot_ is thus difficult to be upscaled from site to global scale, the prior global map of *f*_DEPd, tot_ was set as constant across the globe. As *f*_DEPd, tot_ was frequently reported to be within a range of 5%–15% [44,45] (Table S14), we adopted a central value of 10% for *f*_DEPd, tot_ to estimate *f*_DEPd_. The global map of *f*_recycled, tot_ was estimated from the recycled N of leaf and root tissues before their senescence. With a prescribed value of *f*_DEPd_, the prior global map of *f*_BNFs_ was produced by using a Monte Carlo method based on the theoretical negative relationship between *ε*_U_ and *f*_BNFs_ (Equation (4) and Text S3).

### Estimating symbiotic and total BNF from isotope-based *f*_BNFs_ and ${f}_{{\mathrm{\bf BNF}}_{\mathrm{\bf T}}} $

The global maps of symbiotic and total BNF were estimated as the products of the vegetation-external N demand by isotope-based *f*_BNFs_ and ${f}_{{\mathrm{BNF}}_{\mathrm{T}}}$, respectively (Text S4). To derive ${f}_{{\mathrm{BNF}}_{\mathrm{T}}}$ from *f*_BNFs_, we produced a global map of the fraction of symbiotic fixation in total BNF (*β*_S_) (Fig. S15b) by using the biome-specific *β*_S_ estimates from Table 3 in Davies-Barnard and Friedlingstein [2] and a land-cover map of HYDE v3.2 [46]. To estimate the vegetation-external N demand, we utilized global maps of GPP, C-allocation factors to plant tissues (leaf, wood and root), C:N stoichiometry and NRE (Text S4). Specifically, we adopted three widely used GPP products [29–31] and two sets of C-allocation factors and C:N stoichiometry [32,34], and their uncertainties are discussed in the next paragraph. The 1° × 1° leaf NRE was obtained from the biome-specific NRE values estimated from the datasets provided by Deng *et al.* [47] (Fig. S15a); the root NRE was set as a constant at 0.275 from the ORCHIDEE-CNP model [48]. Notice that we excluded crop and pastural areas from all global maps by following the land-cover map of HYDE v3.2 [46].

To account for the uncertainty from GPP, we utilized three widely used GPP products from MODIS [29], Keenan *et al.* [30] and Jung *et al.* [31]. Moreover, with the GPP product from Keenan *et al.* [30], we tested the impacts of two sets of C-allocation factors and C:N stoichiometry to account for their uncertainties: (i) 1° × 1° C-allocation factors obtained from Bloom *et al.* [32], leaf C:N ratio estimated from a leaf N-concentration map at a 500-m resolution produced by Moreno-Martínez *et al.* [33] and wood and root C:N ratios estimated by multiplying the leaf C:N ratio by the conversion coefficients from the ORCHIDEE model [48] (Table S15); (ii) biome-specific estimates of C-allocation factors and C:N ratios from Wang *et al.* [34]. Note that the C-allocation factors from Wang *et al.* [47] are fractions of NPP rather than GPP; thus, we kept the fraction of respiration in GPP the same as those in Bloom *et al.* [32] and found that the set of parameters from Wang *et al.* [34] results in a 19% lower symbiotic BNF (43.1 versus 53.4 Tg N yr^−1^) and a 17% lower total BNF (74.3 versus 89.8 Tg N yr^−1^).

As *f*_DEPd_ or *f*_DEPd, tot_ could have impacts on *f*_BNF__s_ and ${f}_{{\mathrm{BNF}}_{\mathrm{T}}}$ but with few observations, we also tested the sensitivity of isotope-based BNF estimates to *f*_DEPd, tot_ (Tables S7 and S8). We found that a low boundary of zero for *f*_DEPd_ or *f*_DEPd, tot_ results in the highest estimates of symbiotic and total BNF as 55.2 and 93.6 Tg yr^−1^, respectively (Table S9). Overall, our total BNF is consistent with the C-limited method of the CABLE model (78.1 Tg N yr^−1^) (Table 1) but has a much lower uncertainty compared with the estimate based on meta-analysis (52–130 Tg N yr^−1^) [2]. Compared with previous biome-level BNF estimates [2,5,48], our isotope-based BNF results show higher values in evergreen and deciduous broadleaf forests and grass, but comparable values in evergreen needleleaf forests, and lower values in deciduous needleleaf forests (Table 1).

### Feature selection and partial dependence

We adopted the Shapley (SHAP) values to analyse the feature importance and partial dependence for *f*_BNFs_. The SHAP values, originating from the game theory, have been widely used for evaluating the marginal contribution of individual predictors in predicting the target variables [49]. Here, we selected the RF model to estimate the SHAP values and conduct the analysis. We first adopted the recursive feature elimination method to remove the predictors of less importance. With this method, six predictors were removed from the original 16 predictors and only 10 predictors were kept in the RF models: *T, P*, P/PET, AM and ECM fungi, N-fixers, OC content, clay, NO*_y_* and NH*x* deposition. Next, we adopted the variance inflation factor (VIF), together with correlation coefficients, to remove the predictors with high multicollinearity. We calculated the VIF for the 10 selected predictors (Table S16) and found that ECM, AM, *P*, P/PET and *T* have a VIF of >5, suggesting high multicollinearity for these predictors. We also calculated the correlation matrix of these 10 predictors (Fig. S23) and found that AM and ECM are highly correlated with *R* = –0.96, *P* and P/PET are highly correlated with *R* = 0.75, and *P* and *T* are correlated with *R* = 0.56. Thus, we removed the predictors of AM and *P*, resulting in eight predictors, all with VIF < 5 (Table S17). Finally, we estimated SHAP values for the remaining predictors, retaining the capability of capturing the spatial variations of *f*_BNFs_ (with *R*^2^ unchanged). These analyses were implemented with three well-established packages in Python v3.8.5: RandomForestRegressor, RFE and shap. In each 1° × 1° grid cell, we can obtain a vector of SHAP values, with each element indicating the marginal contribution of a given predictor to the target variable. Across the globe, the means of the absolute SHAP values were used to evaluate the importance of these predictors and ensembles of SHAP values were used to evaluate the partial dependence of the target variables on predictors.

### Comparison of isotope-based *f*_BNFs_ and BNF with CMIP6 models and previous publications

We collected the simulation outputs of BNF and N-uptake fluxes from 11 ESMs (with these two fluxes available) in CMIP6 datasets (Table S4). First, we directly compared the simulated global distributions of total BNF with our isotope-based estimates (Fig. 4). Next, we used the observation-based fraction of symbiotic BNF in total BNF (*β*_S_) to infer *f*_BNFs_ from the CMIP6 models, which were compared with our isotope-based *f*_BNFs_ (Fig. S21). Considering that the CMIP6 datasets do not provide the split of BNF into symbiotic and free-living pathways, we adopted an observation-based global map of *β*_S_ (Fig. S15b) to estimate the symbiotic BNF. Assuming that the fraction of canopy uptake of N deposition as zero (i.e. *f*_DEPd_ = 0, as prescribed in the CMIP6 models), we calculated the *f*_BNFs_ values for the CMIP6 models with Equation (2a) by using the simulated fluxes of total BNF and N uptake. Although this method would have led to some uncertainty in the estimation of *f*_BNFs_, it would not have changed the overall findings in this study. Finally, we compared the BNF values aggregated globally and in four latitudinal bands (60°–90°N, 30°–60°N, 30°S–30°N and 90°–30°S) to our isotope-based estimates (Table S12).

We also compared our isotope-based BNF estimates with those reported in previous publications (Fig. S22). We collected the BNF estimates from previous publications, which are classified based on their representative approaches: (i) CASA-CNP model by Cleveland *et al.* [24] and Peng *et al.* [5]; (ii) δ^15^N-based global N budgets by Vitousek *et al.* [6]; (iii) CLM-FUN2.0 model by Shi *et al.* [25]; and (iv) global meta-analysis by Davies‐Barnard and Friedlingstein [2]. Note that the model-data-based upscaling by Cleveland *et al.* [7] was excluded from our comparison because of its exceptionally high estimates of BNF fluxes. Our biome-specific estimates of symbiotic and/or total BNF were also compared with those simulated by ORCHIDEE-CNP [44], CASA-CNP [5] and the meta-analysis by Davies‐Barnard and Friedlingstein [2] (Table 1), with biomes (plant functional types) aggregated according to the rules specified in Table S18. Moreover, the biome-specific BNFs in unit area from different sources are also compared in Table S19, in which the values for each biome were converted to have a consistent area.

## Supplementary Material

nwaf459_Supplemental_File

## Data Availability

The foliar δ^15^N observations were obtained from Craine *et al.* [18] (https://doi.org/10.5061/dryad.v2k2607). The soil δ^15^N observations were obtained from Craine *et al.* [21] and Sena‐Souza *et al.* [22] (https://esajournals.onlinelibrary.wiley.com/action/downloadSupplement?doi=10.1002%2Fecs2.3223&file=ecs23223-sup-0001-DataS1.zip). The climate data from the CRU TS v4.03 datasets are available at https://catalogue.ceda.ac.uk/uuid/10d3e3640f004c578403419aac167d82. The soil properties from Global Soil Dataset for use in the Earth System Model datasets produced by BNU are available at http://globalchange.bnu.edu.cn/research/soilw. The global maps of the abundance of microbial symbionts (AM, ECM and N-fixers) from Steidinger *et al.* [8] are available at https://static-content.springer.com/esm/art%3A10.1038%2Fs41586-019-1128-0/MediaObjects/41586_2019_1128_MOESM4_ESM.zip. The global maps of the GPP are from three sources: (i) from Keenan *et al.* [30], available at http://cdiac.ornl.gov/GCP/; (ii) from MODIS [29], available at https://lpdaac.usgs.gov/products/mod17a2hv006/; (iii) from Jung *et al.* [31], available at https://www.bgc-jena.mpg.de/geodb/projects/Home.php. The global maps of nitrogen depositions (NH*_x_* and NO*_y_*) were obtained from Tian *et al.* [43] (https://esg.pik-potsdam.de/projects/isimip/). Three sets of global BNF maps simulated by the CSCA-CNP model (with methods A, B and C) from Peng *et al.* [5] were obtained by requesting the data from their corresponding author. All the historical simulation outputs of ESMs are available from CMIP6 (https://esgf-node.llnl.gov/search/cmip6/). The BNF products inferred from N-isotope signals in plant and soil are available at https://doi.org/10.6084/m9.figshare.29468129.v1. The Python codes of the RF models for producing global maps of δ^15^N of plant, soil and their difference are available at https://github.com/myFeng818/Codes_for_Random_Forest_Models_for_d15N.git. The Python codes of the coupled Bayesian approach and Monte Carlo method for inferring BNF from global maps of δ^15^N are available at https://github.com/myFeng818/Codes_for_Bayesian_approach_for_BNF_inference.git.
