# Supplementary material for: Dramatic biases in terrestrial nitrogen fixation in Earth System Models revealed by natural isotope signatures
Source: Natl Sci Rev. 2025 Oct 28;12(12):nwaf459. doi: 10.1093/nsr/nwaf459 (PMC12707069; doi:10.1093/nsr/nwaf459)
Supplement: nwaf459_Supplemental_File [file nwaf459_supplemental_file.docx]

Supplementary Information for

**Dramatic biases in terrestrial nitrogen fixation in Earth System Models revealed by natural isotope signatures**

Maoyuan Feng^1,2,10^, Shushi Peng^1,2^*, Philippe Ciais^3,4^, Daniel S. Goll^3^, Benjamin Z. Houlton^5^, Ying-Ping Wang^6^, Yilong Wang^7^, Pan Liu^8^, Joshua B. Fisher^9^, Pierre Regnier^10^

^1^Sino-French Institute for Earth System Science, College of Urban and Environmental Sciences, and Laboratory for Earth Surface Processes, Peking University, Beijing, China

^2^Institute of Carbon Neutrality, Peking University, Beijing, China

^3^Laboratoire des Sciences du Climat et de l’Environnement, LSCE/IPSL, CEA-CNRS-UVSQ, Université Paris-Saclay, Gif-sur-Yvette, France

^4^The Cyprus Institute, Konstantinou Kavafi Street, Nicosia, Cyprus

^5^Department of Ecology and Evolutionary Biology and Department of Global Development, Cornell University, Ithaca, New York, USA

^6^CSIRO Environment, Private Bag 10, Clayton South VIC 3169, Australia

^7^Key Laboratory of Alpine Ecology, Institute of Tibetan Plateau Research, Chinese Academy of Sciences, Beijing, China

^8^State Key Laboratory of Water Resources Engineering and Management, Wuhan University, Wuhan, China

^9^Schmid College of Science and Technology, Chapman University, Orange, CA, 92866, USA

^10^Department Geoscience, Environment & Society-BGEOSYS, Université Libre de Bruxelles, Brussels, Belgium

*Correspondence to Shushi Peng ([speng@pku.edu.cn](mailto:speng@pku.edu.cn))

**Supporting Text**

# **1. Theoretical negative** **relationship between *f*_BNF_*_s_* and *ε*_U_**

Based on a theoretical model of nitrogen (N) isotope mass balance for a plant-soil system (Fig. 1a in the main), we derived a theoretical negative relationship between the fraction of symbiotic BNF in the vegetation N demand from external sources (here called vegetation-external N demand) (*f*_BNF_*_s_*) and the isotope fractionation of plant N uptake (*ε*_U_). Compared to Houlton *et al.* [1–2], we extended the existing theoretical model by incorporating the previously omitted processes of symbiotic BNF and canopy uptake of N deposition (Fig. 1a in the main). Although rock N weathering is a globally or regionally important flux to terrestrial ecosystems that accounts for 11–20% of new N supply [3], this input occurs mainly in areas of high relief and tectonic uplift that are underlain by N-enriched sedimentary rocks [3], and the fraction of this input that goes into the vegetation N demand, from 1.2 Pg N yr^-1^ [4] to 2.7 Pg N yr^-1^ [5], is relatively small, especially for the environments where the BNF is high. Thus, the isotope effect of this N input is negligible and was not considered in our model. With the theoretical model as shown in Fig. 1a in Box 1, the mass balance of N and ^15^N in the plant and soil pools can be represented as follows:

*dN_s_*/*dt* = (1 − *β*_S_)*I*_BNF_ + (1 − γ_DEPd_)*I*_DEP_ – *L* – *G* + *R* – *U* (1a)

*dN_P_*/*dt* = *β*_S_*I*_BNF_ + γ_DEPd_*I*_DEP_ + *U* – *R* (1b)

*d*^15^*N_S_*/*dt* = (1−*β*_S_)*I*_BNF_⋅*α*_BNF_ + (1 − γ_DEPd_)*I*_DEP_⋅*α*_DEP_ – *L*⋅*α*_L_ – *G*⋅*α*_G_ + *R*⋅*α*_R_ – *U*⋅*α*_U_ (1c)

*d*^15^*N_P_*/*dt* = *β*_S_*I*_BNF_⋅*α*_BNF_ + γ_DEPd_*I*_DEP_⋅*α*_DEP_ + *U*⋅*α*_U_ – *R*⋅*α*_R_ (1d)

where *N_S_* and *N_P_* are N stocks in soil and plant, respectively; ^15^*N_S_* and ^15^*N_P_* are ^15^N stocks in soil and plant, respectively; *t* is the time. *I_DEP_* and *I_BNF_* are N deposition and BNF, respectively; γ_DEPd_ is the fraction of direct canopy uptake of total N deposition, and *β*_S_ is the fraction of symbiotic fixation in total BNF; *U* and *R* are plant uptake and return N fluxes, respectively; *L* and *G* are leaching and gaseous N losses, respectively. *α*_DEP_, *α*_BNF_, *α*_L_, *α*_G_, *α*_R_, and *α*_U_ are fractions of ^15^*N* over ^14^*N* for samples of deposition, BNF, leaching, gas, return and plant uptake fluxes, respectively.

To proceed with the theoretical derivation, we have two primary assumptions: (1) ecosystem gains and losses of N and ^15^N are in balance, and (2) the vegetation-internal N resorption of leaf and root before senescence does not fractionate substantially. First, assuming that the plant-soil system is at or close to the steady state (inputs equal to losses), the changing rates of N stocks in plant and soil equal zero:

(1 − *β*_S_)*I*_BNF_ + (1 − γ_DEPd_)*I*_DEP_ – *L* – *G* + *R* – *U =0* (2a)

*β*_S_*I*_BNF_ + γ_DEPd_*I*_DEP_ + *U* – *R =* 0 (2b)

Similarly, the mass balance equations of ^15^N stocks can be represented as:

(1−*β*_S_)*I*_BNF_⋅*α*_BNF_ + (1 − γ_DEPd_)*I*_DEP_⋅*α*_DEP_ – *L*⋅*α*_L_ – *G*⋅*α*_G_ + *R*⋅*α*_R_ – *U*⋅*α*_U_ *=*0 (3a)

*β*_S_*I*_BNF_⋅*α*_BNF_ + γ_DEPd_*I*_DEP_⋅*α*_DEP_ + *U*⋅*α*_U_ – *R*⋅*α*_R_*=*0 (3b)

Combining Eq. (2) with Eq. (3) and replacing the fractions of ^15^*N* over ^14^*N* (*α*) with natural N isotope ratios (δ^15^N) for soil and plant could result in the following equations:

(1 − γ_DEPd_)*I*_DEP_ ⋅*δ*_DEP_+ (1 − *β*_S_)*I*_BNF_⋅*δ*_BNF_– *L*⋅*δ*_L_ – *G*⋅*δ*_G_ + *R*⋅*δ*_R_ – *U*⋅*δ*_U_ =0 (4)

γ_DEPd_*I*_DEP_⋅*δ*_DEP_ + *β*_S_*I*_BNF_⋅*d*_BNF_+ *U*⋅*δ*_U_ – *R*⋅*δ*_R_ =0 (5)

where *δ*_DEP_, *δ*_BNF_, *δ*_L_, *δ*_G_, *δ*_R_, and *δ*_U_ are natural N isotope ratios for deposition, BNF, leaching, gas, return and uptake fluxes, respectively.

Specifically, *δ*_U_, *δ*_L_, and *δ*_G_ can be represented by soil δ^15^N and respective isotope fractionations, while *δ*_R_ equals to δ^15^N in plant tissues (assumption (2)):

*δ*_U_ = *δ*_S_ – *ε*_U_ (6a)

*δ*_L_ = *δ*_S_ – *ε*_L_ (6b)

*δ*_G_ = *δ*_S_ – *ε*_G_ (6c)

*δ*_R_ = *δ*_P_ (6d)

where *δ*_S_ and *δ*_P_ are N isotope ratios of soil and plant, respectively; *ε*_U_, *ε*_L_, and *ε*_G_ are effective fractionation factors for plant N uptake, leaching and gaseous N losses, respectively.

Substituting Eqs. (6a)–(6d) into Eqs. (4) and (5) results in the following equations:

(1 − γ_DEPd_)*I*_DEP_ ⋅*δ*_DEP_+ (1−*β*_S_)*I*_BNF_⋅*δ*_BNF_– *L*⋅(*δ*_S_ – *ε*_L_) – *G*⋅(*δ*_S_ – *ε*_G_) + *R*⋅*δ*_P_ – *U*⋅(*δ*_S_ – *ε*_U_) =0 (7)

γ_DEPd_*I*_DEP_⋅*δ*_DEP_ + *β*_S_*I*_BNF_⋅*δ*_BNF_+ *U*⋅(*δ*_S_ – *ε*_G_) – *R*⋅*δ*_R_ =0 (8)

In this study, we focus on N isotope mass balance of the plant (Eqs. (5) and (8)), which was used to derive the fraction of vegetation-external N demand satisfied by symbiotic BNF (*f*_BNFs_). Dividing both sides of Eq. (8) by *R*=*U*+*β*_S_*I*_BNF_+γ_DEPd_*I*_DEP_ (Eq. (2b)), we obtained:

$\text{δ}_{\text{P}}\text{=}\left( \text{1 }\text{-}\text{ }\text{f}_{\text{BNFs}}-\text{f}_{\text{DEPd}} \right)\left( \text{δ}_{\text{S}} \text{-}\text{ }\text{ε}_{\text{U}} \right)\text{ + }\text{f}_{\text{BNFs}}\text{δ}_{\text{BNF}}+\text{f}_{\text{DEPd}}\text{δ}_{\text{DEP}}$ (9)

where

$\text{f}_{\text{BNFs}}\text{=}\frac{\text{β}_{\text{s}}\text{I}_{\text{BNF}}}{\text{β}_{\text{s}}\text{I}_{\text{BNF}}\text{+}\text{γ}_{\text{d}}\text{I}_{\text{DEP}}\text{ +}\text{U}}$ (10a)

$\text{f}_{\text{DEPd}}\text{=}\frac{\text{γ}_{\text{d}}\text{I}_{\text{DEP}}}{\text{β}_{\text{s}}\text{I}_{\text{BNF}}\text{+}\text{γ}_{\text{d}}\text{I}_{\text{DEP}}\text{ +}\text{U}}$ (10b)

Assuming *f*_DEPd_ is known, solving *f*_BNFs_ as an unknown term from Eq. (9) results in a theoretical relationship between *f*_BNFs_ and δ_P_−δ_S_, δ_DEP_, δ_BNF_, *f*_DEPd_ and *ε*_U_, as follows:

$\text{f}_{\text{BNFs}}\text{= }\text{-}\text{ }\frac{\left( \text{δ}_{\text{P}}\text{ }\text{-}\text{ }\text{δ}_{\text{S}} \right) \text{+} \text{ε}_{\text{U}}}{\text{δ}_{\text{S}}\text{ }\text{-}\text{ }\text{δ}_{\text{BNF}\text{ }}\text{-}\text{ }\text{ε}_{\text{U}}}\text{ }\text{-}\text{ }\text{f}_{\text{DE}\text{P}_{\text{d}}}\frac{\text{δ}_{\text{S}}\text{ }\text{-}\text{ }\text{δ}_{\text{DEP}}\text{ }\text{-}\text{ }\text{ε}_{\text{U}}}{\text{δ}_{\text{S}}\text{ }\text{-}\text{ }\text{δ}_{\text{BNF}}\text{ }\text{-}\text{ }\text{ε}_{\text{U}}}$ (11)

Inversely, the isotope fractionation of plant N uptake *ε*_U_ can be represented by the fraction of symbiotic BNF *f*_BNFs_ and plant and soil δ^15^N signals:

$\text{ε}_{\text{U}}\text{ }\text{=}\text{ }\text{δ}_{\text{S}}\text{ }\text{-} \text{δ}_{\text{BNF}}\text{ +} \frac{{\text{(}\text{δ}}_{\text{BNF}}\text{-} \text{δ}_{\text{P}}\text{)}\text{ }\text{+}\text{ }\text{f}_{\text{DEPd}}\text{(}\text{δ}_{\text{DEP}}\text{ }\text{-}\text{ }\text{δ}_{\text{BNF}}\text{)}}{\text{1 }\text{-}\text{ }\text{f}_{\text{BN}\text{F}_{\text{s}}}\text{-}\text{ }\text{f}_{\text{DEPd}}}$ (12)

Since ${\text{(}\text{δ}}_{\text{BNF}}\text{-} \text{δ}_{\text{P}}\text{) + }\text{f}_{\text{DEPd}}\text{(}\text{δ}_{\text{DEP}}\text{ }\text{-}\text{ }\text{δ}_{\text{BNF}}\text{)<0}$ holds for almost all cases, Eq. (12) indicates a theoretical negative relationship between ε_U_ and *f*_BNFs_. With this relationship, we can have the maximum of ε_U_ when *f*_BNFs_ equals zero, i.e., ε_U,max_ = δ*_S_* − δ*_P_* + *f*_DEPd_(δ_DEP_ − δ_P_)/(1 − *f*_DEPd_).

With the theoretical negative relationship between *f*_BNFs_ and *ε*_U_ as specified by Eqs. (11) and (12), we first use the assumed parameters (*δ*_S_, *δ*_P_, *δ*_BNF_, *δ*_DEP_ and *f*_DEPd_) to test how these parameters may affect the theoretical curves (Fig. S1). Generally, *δ*_S_ >*δ*_P_ >*δ*_BNF_ holds for most cases of plant-soil system (Figs. S1–S2). In Fig. S1a, as *δ*_P_ is decreased to approach *δ*_BNF_, the theoretical curve moves from the bottom left to top right corner, and finally becomes a horizontal line indicating a level of *f*_BNFs_ = 1. This implies that for a prescribed level of *ε*_U_, the isotope-based *f*_BNFs_ is more likely to increase as *δ*_P_ decreases, and it will always be 1 when *δ*_P_ equals *δ*_BNF_. In Fig. S1b, the increase of *δ*_S_ will drive the theoretical curve to move from right to left parts of the panel, indicating minor decreases in the isotope-based *f*_BNFs_. In Fig. S1c, the increase of *δ*_BNF_ will drive the theoretical curve to move from bottom to top parts of the panel, indicating an increase in the isotope-based *f*_BNFs_. Finally, in Fig. S1d, the increase of *f*_DEPd_ will drive the theoretical curve to rotate in anticlockwise direction. This implies that the change of *f*_DEPd_ could change the isotope-based *f*_BNFs_ slightly, but whether it is increased or decreased by *f*_DEPd_ depends on parameter *ε*_U_ that is determined by N fluxes from different acquisition pathways and respective fractionations. Overall, the theoretical curves are more sensitive to the isotope signatures (*δ*_S_, *δ*_P_, and *δ*_BNF_), over the fraction *f*_DEPd_.

Next, to illustrate the theoretical negative relationship between *f*_BNFs_ and *ε*_U_ in the real world, we collected realistic paired field observations of plant and soil δ^15^N for ecosystems with different types of microbial symbionts (N fixing bacteria (N-fixers), ecto-mycorrhizal (ECM) and arbuscular mycorrhizal (AM) fungi). The paired field observations were collected at Brazilian savannas (15°56’41’’S) [6], North Caucasus (43°27’N) [7], and Hawi’i (19°00’–20°20’N) [8]. Since plants with ECM have higher *ε*_U_ and lower δ_P_ compared to those with AM and N fixing bacteria (N-fixers), we can simply separate all observations into ECM and non-ECM groups (AM and N-fixers) based on the magnitudes of δ_P_ and δ_BNF_. We used the 1:1 line to divide the panel with coordinates of δ_S_-δ_P_ and δ_S_-δ_BNF_ into two zones (Fig. S2): (1) ECM zone where δ_S_–δ_P_≥δ_S_–*δ*_BNF_, i.e., δ_P_≤δ_BNF_, the plant δ^15^N is dominated by the isotope fractionation of plant N uptake through ECM, and *ε*_U_ is pretty high and could reach the potential maximum with *f*_BNF_*_s_* equal zero. (2) AM and N-fixers zone where δ_S_–δ_P_<δ_S_–δ_BNF_, i.e., δ_P_>δ_BNF_, the plant δ^15^N is co-regulated by the isotope fractionation of plant N uptake through AM and the isotope signature of BNF. In Fig. S2, some ECM-dominated observations fall in the AM and N-fixers zone while few AM-dominated observations fall in ECM zone. This is probably due to the large uncertainty of site observations of plant and soil δ^15^N. In particular, the high uncertainty of soil δ^15^N may be attributed to the large variations of δ^15^N measurements across vertical profile of soil column. Moreover, for the ECM-dominated system, we have a high but uncertain *ε*_U_ (which can be approximated as δ_S_–δ_P_) and a zero value of *f*_BNF_*_s_*, which is difficult to be illustrated by the theoretical negative relationship but should be represented as single points. Thus, in the following, these abnormal observational points are excluded, and we illustrated only the plants with AM and N-fixers (Figs. S3–S4). Fig. S3 shows the theoretical negative relationship between *f*_BNFs_ and *ε*_U_ at Brazilian savannas, where plants have both N-fixers and AM. We found that plants with N-fixers have systematically higher *f*_BNFs_ and thus their curves are located at upper and righter positions within the panel, compared to those with AM. Fig. S4 compares theoretical negative relationships in the Alpine of North Caucasus and Hawi’i, where all plants have microorganisms of AM.

# **2. Training and testing of Random Forest models**

With 38646 foliar δ^15^N measurements from Craine *et al.* [9] and 5887 soil δ^15^N measurements from Craine *et al.* [10] and Sena‐Souza *et al.* [11], we adopted machine learning techniques to produce global maps of plant and soil δ^15^N (δ_P_ and δ_S_) and their difference (δ_P_ − δ_S_). The foliar and soil δ^15^N measurements were aggregated into 2431 and 956 0.1°×0.1° grid cells, respectively (Fig. S5). Most data sites are distributed in Northern America, Europe, southeast Asia and Australia while only a small portion of data sites are distributed in boreal regions. This could cause some systematic bias for the δ^15^N predictions in boreal regions. Note that we used foliar δ^15^N to represent the plant δ^15^N because of the little difference of δ^15^N across plant organs (leaf, wood, root) [12]. To produce global maps of δ_P_, δ_S_ and their difference (δ_P_ − δ_S_), we collected 16 predictors (three climate drivers, three abundances of microbial symbionts, seven soil properties, GPP, NHx and NOy depositions) from global datasets (see Methods; Table S3).

We used three separate Random Forest (RF) models to produce global maps of δ_P_, δ_S_ and δ_P_ − δ_S_. By excluding grid cells where any of the 16 predictors have invalid or missing values, we used plant δ^15^N observations in the remaining 2224 grid cells and soil δ^15^N observations in the remaining 933 0.1°×0.1° grid cells to train the random forest (RF) models. As for upscaling the global map for δ_P_ − δ_S_, we need pairwise observations of δ_P_ and δ_S_. Considering the lack of *naturally* pairwise observations of δ_P_ and δ_S_, we collected δ_S_ from RF-produced global map at sites of δ_P_ as quasi-observations of δ_S_ to establish the *artificial* pairwise observations. This assumes that our δ_S_ map is reliable enough and could be considered as quasi-observations. For each of the three RF models, we trained the model by adopting 500 decision trees with a well-established package, RandomForestRegressor, in Python v3.8.5. For each decision tree, the bootstrap strategy was activated for sampling the predictors, the maximum feature was set as square root of feature numbers (i.e., 4 features), and the minimum leaf of the decision tree was set as 1. The training and testing of the RF models for δ_P_, δ_S_ and δ_P_ − δ_S_ were shown in Fig. S6. To examine the reliability of the RF models, we further conducted a *K*-fold (*K*=10) cross validation by training the three RF models with 90% samples and testing the models with the remaining 10% samples. The training and testing processes were repeated by 100 times, resulting in the frequency distribution of the variance explained by the RF model as in Fig. S7. For all the three RF models, we found that withholding 10% of samples could only slightly degenerate the model performance, i.e., decrease the *R*^2^ in the model validation by <5%, indicating that the RF models were robust to predict δ_P_, δ_S_ and δ_P_ − δ_S_.

For the plant δ^15^N, the RF model has *R*^2^ as 0.88 and 0.49 for the training and testing, respectively, and has RMSE as 1.29‰ and 2.66‰ for the training and testing, respectively (Fig. S6a and S6b). Compared to the linear regression model by Amundson *et al.* [13], across the 2224 grid cells with observations available, our RF model increased *R*^2^ from 0.22 to 0.72 while decreased RMSE from 3.28‰ to 2.01‰ (Fig. S8a and S8b). For the soil δ^15^N, the RF model has *R*^2^ as 0.92 and 0.55 for the training and testing, respectively, and has RMSE as 0.77‰ and 1.83‰ for the training and testing, respectively (Fig. S6c and S6d). Compared to the linear regression model by Amundson *et al.* [13], across the 933 grid cells with observations available, our RF models increased *R*^2^ from 0.21 to 0.66 while decreased RMSE from 2.74‰ to 1.61‰ (Fig. S8c and S8d). For the difference between plant and soil δ^15^N (δ_P_-δ_S_), the RF model has *R*^2^ as 0.86 and 0.40 for the training and testing, respectively, and has RMSE as 1.29‰ and 2.65‰ for the training and testing, respectively (Fig. S6e and S6f). Compared to the linear regression model by Amundson *et al.* [13], across the 2224 grid cells where the artificial pairwise observations are available, our RF model increased *R*^2^ from 0.14 to 0.59 while decreased RMSE from 6.66‰ to 4.87‰ (Fig. S8e and S8f). The global map of δ_P_ δ_P_, δ_S_ and δ_P_ − δ_S_ were shown in Fig. S9, while the uncertainties (quantified by standard deviations (SDs)) for δ_P_, δ_S_ and δ_P_ − δ_S_ were shown in Fig. S10.

# **3. Implementation of the Bayesian approach at global scale**

With global maps of δ_P_ and δ_S_, the implementation of Bayesian approach at global scale needs the parameters of δ^15^N signals of BNF and N deposition (δ_BNF_ and δ_DEP_), as well as the prior global maps of isotope fractionation of plant uptake (*ε*_U_), and the fractions of vegetation-external N demand satisfied by canopy uptake of N deposition (*f*_DEPd_) and symbiotic BNF (*f*_BNF_*_s_*). Following Denk *et al.* [14], δ_BNF_ was in the range of −2.02 ± 2.2‰, and herein we set δ_BNF_ as a constant of −2.02‰ across the globe (Table S13). The δ_DEP_ was reported to be within a range of −3–3‰ [2]; herein we adopted a value of 1.5‰ since the observations from ice core indicate a positive value of δ_DEP_ for natural terrestrial ecosystems in recent decades [15–16]. Empirically, the prior *ε*_U_ depends on relative fractions of N uptake fluxes from three pathways, i.e., (1) arbuscular mycorrhizal (AM), (2) ectomycorrhizal (ECM) fungi, (3) and non-mycorrhizal or direct root uptake. Thus, we assumed *ε*_U_ as a linear combination of relative fractions of N uptake from three different pathways and their respective isotope effects, i.e.,

*ε*_U_=*ε*_AM_*f*_AM_+*ε*_ECM_*f*_ECM_+*ε*_root_*f*_root_ (13)

where *ε*_AM_, *ε*_ECM_, and *ε*_root_ are isotope fractionation factors for N uptake pathways from AM, ECM, and non-mycorrhizal roots, respectively; *f*_AM_, *f*_ECM_, and *f*_root_ are relative fractions of N uptake fluxes from AM and ECM, and roots, respectively. Specifically, Denk *et al.* [14] reported that isotope fractionations for the plant N uptake without mycorrhizal fungi (i.e., non-mycorrhizal plants) are –7.27±4.07‰ and –7.5±0.97‰, respectively, for the N forms of NO_3_^-^ and NH_4_^+^. Moreover, Craine *et al.* [12] indicated that plants with AM and ECM fungi have their plant δ^15^N more depleted by 2‰ and 5.9‰, respecitively, compared to non-mycorrhizal plants. Thus, we adopted the fractionation factors for N uptake from AM, ECM, and non-mycorrhizal roots as –9.35, –13.29, and –7.35‰ (calculated as the mean of –7.27‰ and –7.5‰), respectively. The relative fractions of N uptake fluxes from different pathways could be obtained from the most recent Fixation and Uptake of Nitrogen Model (FUN v3.0) [17]. Therefore, the global map of *ε*_U_ could be produced with Eq. (13) and shown in Fig. S14.

As summarized in Table S14, the vegetation canopy was reported to be able to retain 16–80% of the total N deposition, which could meet 5–40% of total vegetation N demand. As the fraction of canopy uptake of N deposition in total vegetation N demand (*f*_DEPd,tot_) was frequently reported to be within a range of 5–15%, we adopted a central value of 10% for *f*_DEPd,tot_ and tested the sensitivity of *f*_DEPd,tot_ from 5% to 15% in our analysis. As direct measurements of *f*_DEPd,tot_ are very limited in the current literature, we set *f*_DEPd,tot_ as constant across the globe. With a known *f*_DEPd,tot_, the fraction of canopy uptake of N deposition in the vegetation-external N demand (*f*_DEPd_) could be estimated with the fraction of vegetation-internal recycled N in the total vegetation N demand (*f*_recycled,tot_), i.e., *f*_DEPd_ = *f*_DEPd,tot_/(1 − *f*_recycled,tot_). This fraction *f*_recycled,tot_ could be estimated with C allocation factors, C:N stoichiometries and N resorption efficiencies for plant tissues (leaf, wood, and root):

$\text{f}_{\text{recycled, tot}}\text{= }\frac{\frac{\text{A}\text{F}_{\text{leaf}}}{\text{C}\text{N}_{\text{leaf}}}\text{NR}\text{E}_{\text{leaf}}\text{+}\frac{\text{A}\text{F}_{\text{root}}}{\text{C}\text{N}_{\text{root}}}\text{NR}\text{E}_{\text{root}}}{\frac{\text{A}\text{F}_{\text{leaf}}}{\text{C}\text{N}_{\text{leaf}}}\text{+}\frac{\text{A}\text{F}_{\text{wood}}}{\text{C}\text{N}_{\text{wood}}}\text{+}\frac{\text{A}\text{F}_{\text{root}}}{\text{C}\text{N}_{\text{root}}}}$ (14)

where *AF_leaf_*, *AF_wood_*, and *AF_root_* are allocation factors of GPP to leaf, wood, and root, respectively (*AF_leaf_* + *AF_wood_* + *AF_root_* <1); *CN_leaf_*, *CN_wood_*, and *CN_root_* are target C/N ratios of leaf, wood and root, respectively; *NRE_leaf_* and *NRE_root_* are the N resorption efficiencies of leaf and root, respectively. Based on Eq. (14), we could obtain a global map of *f*_recycled,tot_ which could be further used to derive the global map of *f*_DEPd_ with the equation of *f*_DEPd_ = *f*_DEPd,tot_/(1 − *f*_recycled,tot_).

With prior global maps of *ε*_U_ and *f*_DEPd_ estimated as above, we used a Monte Carlo (MC) approach to produce a prior global map of *f*_BNFs_ by applying the theoretical negative relationship between *ε*_U_ and *f*_BNF_*_s_* (Eq. (11) or Eq. (3) in Box 1 in the main text). Within each 1°×1° grid cell, the observations of δ_P_ and δ_S_ and the parameters of *δ*_BNF_, *δ*_DEP_, *ε*_U_, and *f*_DEPd_ were assumed as Gaussian distributions, and the MC approach was implemented according to Eq. (11). The uncertainties or standard deviations (SDs) of δ_P_ and δ_S_ were captured by ensembles produced by the RF models. The SD of *ε*_U_ was specified as 30% of the value (the impact of this fraction on the derived mean of *f*_BNF_*_s_* was tested and shown to be minor). Note that, due to the stochasticity of the approach, some individual ensembles may violate the constraints of our isotope mass balance model (e.g., non-negative condition) on parameters *f*_BNF_*_s_*, *f*_DEPd_, and *ε*_U_ and thus need to be corrected as follows:

(1) if ${\text{f}\text{ }}_{\text{BNFs}}^{\text{i}}\text{<0}$ (*i* is the index of the ensemble), the fraction ${\text{f}\text{ }}_{\text{BNFs}}^{\text{i}}$ is set as zero, and $\text{ε}_{\text{U}}^{\text{i}}$ is set as the potential maximum, i.e., $\text{δ}_{\text{S}}^{\text{i}}\text{-}\text{δ}_{\text{P}}^{\text{i}}+\text{ }\text{f}_{\text{DEPd}}^{\text{i}}\text{(}\text{δ}_{\text{DEP}}\text{-}\text{ }\text{δ}_{\text{P}}^{\text{i}}\text{)/(1}\text{-}\text{ }\text{f}_{\text{DEPd}}^{\text{i}}\text{)}$;

(2) if ${\text{f}\text{ }}_{\text{BNFs}}^{\text{i}}\text{≥}\text{1}$, the specific ensembles are excluded in the analysis;

(3) if $\text{δ}_{\text{P}}^{\text{i}}<\delta_{BNF}$, the fraction ${\text{f}\text{ }}_{\text{BNFs}}^{\text{i}}$ is set as zero, and $\text{ε}_{\text{U}}^{\text{i}}$ is set as $\text{δ}_{\text{S}}^{\text{i}}\text{-}\text{δ}_{\text{P}}^{\text{i}}+\text{ }\text{f}_{\text{DEPd}}^{\text{i}}\text{(}\text{δ}_{\text{DEP}}\text{-}\text{ }\text{δ}_{\text{P}}^{\text{i}}\text{)/(1}\text{-}\text{ }\text{f}_{\text{DEPd}}^{\text{i}}\text{)}$;

(4) if $\text{ε}_{\text{U}}^{\text{i}}<0$, the specific ensembles are excluded in the analysis.

In the processes of producing a prior global map of *f*_BNF_*_s_*, above corrections would update the global map of *ε*_U_. Due to the theoretical negative relationship between *ε*_U_ and *f*_BNF_*_s_*, this updated *ε*_U_ could in turn affect the global map of *f*_BNF_*_s_* if we apply the MC method again. Thus, we repeated the MC approach iteratively to produce a stable global map of *f*_BNF_*_s_* (Fig. S25a) and a corresponding stable global map of *ε*_U_ (Fig. S26a). However, this constrained global map of *f*_BNF_*_s_* is stable but not optimal because the prior global map of *ε*_U_ was assumed based on our empirical knowledge (Eq. (13)). To overcome this problem, we used a Bayesian approach to derive optimal estimates of *f*_BNF_*_s_*, *f*_DEPd_, and *ε*_U_ simultaneously.

With the prior global map of *f*_DEPd_ derived from *f*_DEPd_ = *f*_DEPd,tot_/(1 – *f*_recycled,tot_) (Eq. (14)) and prior global maps of *f*_BNF_*_s_* (Fig. S25a) and *ε*_U_ (Fig. S26a) derived using MC approach, we used a Bayesian approach to obtain optimal estimates of *f*_BNF_*_s_*, *f*_DEPd_, and *ε*_U_ (see Methods). Similar to the procedures as described for the MC method, the standard Bayesian approach was first implemented without considering the involved physical constraints; after the global maps of *f*_BNF_*_s_*, *f*_DEPd_, and *ε*_U_ converge to stable ones, we apply the constraints to correct the stable global maps, as in the MC approach. Finally, the posterior global map of *f*_BNF_*_s_* was obtained as Fig. 2a in the main, and the maps of *ε*_U_ and *f*_DEPd_ was obtained as Fig. S24a and S24c, respectively.

We used the global maps of *f*_BNF_*_s_* produced by the MC method and the Bayesian approach to derive global maps of $\text{f}_{\text{BNF}_{\text{T}}}$ (Fig. S11c) as well as symbiotic and total BNF (Fig. S18). Compared to the MC-based results, the Bayesian approach decreases the values of *f*_BNF_*_s_* and $\text{f}_{\text{BNF}_{\text{T}}}$ across the globe. Furthermore, the estimates of global symbiotic and total BNF derived from Bayesian approach were lower than those obtaind from MC method (Table S6 versus Table S20). Similarly, when the *f*_DEPd,tot_ is set as the lower boundary of *f*_DEPd,tot_ =0, the estimates of global symbiotic and total BNF derived from Bayesian approach were also lower than those obtaind from MC method (Table S9 versus Table S21).

# **4. From *f*_BNF_*_s_* and** $\text{f}_{\text{BNF}_{\text{T}}}$ **to symbiotic and total BNF**

We estimated symbiotic and total BNF as the products of vegetation-external N demand (sum of canopy uptake of N deposition, symbiotic BNF and plant N uptake) by *f*_BNF_*_s_* and $\text{f}_{\text{BNF}_{\text{T}}}$, respectively. First, we estimated the vegetation-external N demand ($\text{D}_{\text{N}}^{\text{e}}$) by deducting vegetation-internal recycled N from total vegetation N demand, which is estimated as the product of tissue growth (i.e., leaf, wood, and root) and C:N stoichiometry, i.e.,

$\text{D}_{\text{N}}^{\text{e}}\text{=GPP∙}\text{(}\frac{\text{A}\text{F}_{\text{leaf}}}{\text{C}\text{N}_{\text{leaf}}}\left( \text{1}\text{-NR}\text{E}_{\text{leaf}} \right)\text{+}\frac{\text{A}\text{F}_{\text{wood}}}{\text{C}\text{N}_{\text{wood}}}\text{+}\frac{\text{A}\text{F}_{\text{root}}}{\text{C}\text{N}_{\text{root}}}\left( \text{1}\text{-NR}\text{E}_{\text{root}} \right)\text{)}$ (15)

Then, the symbiotic BNF can be estimated as follows:

*BNF_S_*=*f*_BNFs_⋅$\text{D}_{\text{N}}^{\text{e}}$ (16)

Similarly, total BNF can be calculated as follows:

*BNF_T_*=$\text{f}_{\text{BN}\text{F}_{\text{T}}}\cdot\text{D}_{\text{N}}^{\text{e}}$ (17)

where GPP is the gross primary production; *AF_leaf_*, *AF_wood_*, and *AF_root_* are allocation factors of GPP to leaf, wood, and root, respectively (*AF_leaf_* + *AF_wood_* + *AF_root_* <1); *CN_leaf_*, *CN_wood_*, and *CN_root_* are target C/N ratios of leaf, wood and root, respectively; *NRE_leaf_* and *NRE_root_* are N resorption efficiency of leaf and root, respectively.

The global maps of GPP were obtained from three widely used products: MODIS-GPP [18], Keenan *et al.* [19], and Jung *et al.* [20]. The C allocation factors (*AF_leaf_*, *AF_wood_*, and *AF_root_*) and C:N ratios were from two sources: (1) global maps of C allocation factors from Bloom *et al.* [21], the leaf C:N ratios from the leaf nitrogen concentration (LNC) from Moreno-Martínez *et al.* [22] (Fig. S28a; *CN_leaf_*=0.45/LNC, where 0.45 is the leaf C mass in unit of gC m^-2^ yr^-1^), and C:N ratios of wood (Fig. S28b) and root (Fig. S28c) estimated with leaf C:N ratio and conversion coefficients in ORCHIDEE model [23] (Table S15). (2) biome-specific estimates of C allocation factors and C:N ratios from Wang *et al.* [24]. Note that the C allocation factors from Wang *et al.* [24] are for NPP rather than GPP, thus we retained the same fraction of C allocated to auto respiration as those in Bloom *et al.* [21]. In the main, we presented the results from the first set of C allocation factors and C:N ratio as the benchmark, considering that the latest global products could result in a more reliable estimation and the biome-specific parameters would lead to a higher uncertainty. The global map of *NRE_leaf_* were obtained by assigning a constant value for a specific biome (Fig. S15), which was estimated from the datasets compiled by Deng *et al.* [25]. The global map of *NRE_root_* were set as constant (0.275) across the globe according to the settings in ORCHIDE-CNP [48]. With GPP and parameters as above, we estimated the global map of vegetation-external N demand as Fig. S16.

# **5. Posterior global map of *f*_DEPd_ and ε_U_**

We applied the Bayesian approach iteratively and finally obtained stable posterior global maps of *f*_BNF_*_s_*, *f*_DEPd_, and *ε*_U_ simultanuously. As the posterior global map of *f*_BNF_*_s_* has been discussed in the main, here we show the posterior global maps of *f*_DEPd_ and *ε*_U_ (Fig. S24). The posterior *f*_DEPd_ is higher in some temperate regions, and the highest value could be up to 0.14, leading to a global mean of 0.11, which is slightly higher than the prior global constant of *f*_DEPd,tot_ as 0.10. The latitudinal gradient of *f*_DEPd_ is almost constant across the globe. The posterior global map of *ε*_U_ has a global mean of 4.1‰, with the maximum larger than 7.2‰ in boreal region and the lowest close to zero in tropical region. The latitudinal mean of *ε*_U_ increases from 1.2‰ in the tropical regions to 5.7‰ in the boreal regions. This is consistent with our common knowledge that the plants in boreal regions have ECM and thus higher isotope effects compared to those in tropical regions with AM [14, 30]. Compared to the prior global map, the posterior *ε*_U_ has a very similar spatial pattern but with lower magnitudes (Fig. S26). In tropical and most of temperate regions, the posterior *ε*_U_ optimzed by the Bayesian approach is determined by the theoretical negative relationship between *ε*_U_ and *f*_BNF_*_s_* and constrained by observations, and thus the magnitude of *ε*_U_ is determined by the competitive relationship between symbiotic BNF and plant N uptake through AM and ECM pathwyas (where *f*_BNF_*_s_*>0). In most of boreal regions, the magnitude of *ε*_U_ is determined by plant N uptake through ECM pathway (where *f*_BNF_*_s_*=0) and thus equals to the maximum of δ*_S_* – δ*_P_* + *f*_DEPd_(δ_DEP_ – δ_P_)/(1 – *f*_DEPd_).

We also found that the posterior global map of *ε*_U_ is lower than the previously reported values in the literature (Table S13). This can be explained as that the previously reported values are likely to reflect the fractionation in the laboratory incubation conditions, whereas the Bayesian-based estimates of *ε*_U_ are more likely to reflect the effective fractionation in the fields and at a large spatial scale. In the field and at a large spatial scale, *ε*_U_ should be *effective* fractionation, which takes into account the underestimation of the fractionation due to the consumption of available N [1, 28], i.e.,

$\text{ε}_{\text{U}}\text{=}{\text{–}\text{ε'}}_{\text{U}}\frac{\text{(1-}\text{p}\text{)log(1-}\text{p}\text{)}}{\text{p}}$ (18)

where *ε*'_U_ is the fractionation factor of plant uptake without accounting for the underestimation due to the consumption of available N, and *p* is the fraction of plant uptake over all available N. –[(1-*p*)log(1-*p*)/*p*] is the factor that accounts for the underestimation of fractionation factor , i.e., it is approaching 1 when *p*≈0, and the underestimation occurs when *p*$\text{≫}$0 [1].

# **References**

1. B. Z. Houlton, D.M. Sigman, L.O. Hedin, Isotopic evidence for large gaseous nitrogen losses from tropical rainforests. *Proc. Natl. Acad. Sci. U. S. A.*, **103**, 8745-8750 (2006).

2. B. Z. Houlton, A. Marklein, E. Bai, Representation of nitrogen in climate change forecasts. *Nature Clim. Change,* **5**, 398-401 (2015).

3. B. Z. Houlton, S. L. Morford, R. A. Dahlgern, Convergent evidence for widespread rock nitrogen sources in Earth's surface environment. *Science*, **360**, 58-62 (2018).

4. C. C. Cleveland, B. Z. Houlton, W. K. Smith, A. R. Marklein, S. C. Reed, W. Parton, S. J. Del Grosso, S. W. Running, Patterns of new versus recycled primary production in the terrestrial biosphere. *Proc. Natl. Acad. Sci. U. S. A.*, **110**, 12733-12737 (2013).

5. M. Shi, J. B. Fisher, E. R. Brzostek, R. P. Phillips, Carbon cost of plant nitrogen acquisition: global carbon cycle impact from an improved plant nitrogen cycle in the Community Land Model. *Global Change Biol.,* **22**, 1299-1314 (2016).

6. M. M. C. Bustamante, L. A. Martinelli, D. A. Silva, P. B. Camargo, C. A. Klink, T. F. Domingues, R. V. Santos, ^15^N natural abundance in woody plants and soils of Central Brazilian Savannas (Cerrado). *Ecol. Appl.,* **14**, 200-213 (2004).

7. M. I. Makarov, B. Glaser, W. Zech, T. I. Malysheva, I. V. Bulatnikova, A.V. Volkov, Nitrogen dynamics in alpine ecosystems of the northern Caucasus. *Plant Soil*, **256**, 389-402 (2003).

8. A. T. Austin, P. M.Vitousek, Nutrient dynamics on a precipitation gradient in Hawai'i. *Oecologia,* **113**, 519-529 (1998).

9. J. M. Craine, A. J. Elmore, L. Wang, J. Aranibar, M. Bauters, P. Boeckx, B. E. Crowley, M. A. Dawes, S. Delzon, A. Fajardo, Y. Fang, L. Fujiyoshi, A. Gray, R. Guerrieri, M. J. Gundale, D. J. Hawke, P. Hietz, M. Jonard, E. Kearsley, T. Kenzo, M. Makarov, S. Marañón-Jiménez, T. P. McGlynn, B. E. McNeil, S. G. Mosher, D. M. Nelson, P. L. Peri, J. C. Roggy, R. Sanders-DeMott, M. Song, P. Szpak, P. H. Templer, D. Van der Colff, C. Werner, X. Xu, Y. Yang, G. Yu, K. Zmudczyńska-Skarbek, Isotopic evidence for oligotrophication of terrestrial ecosystems. *Nat. Ecol. Evol.,* **2**, 1735-1744 (2018).

10. J. M. Craine, A. J. Elmore, L. Wang, L. Augusto, W. T. Baisden, E. N. J. Brookshire, M. D. Cramer, N. J. Hasselquist, E. A. Hobbie, A. Kahmen, K. Koba, J. M. Kranabetter, M. C. Mack, E. Marin-Spiotta, J. R. Mayor, K. K. McLauchlan, A. Michelsen, G. B. Nardoto, R. S. Oliveira, S. S. Perakis, P. L. Peri, C. A. Quesada, A. Richter, L. A. Schipper, B. A. Stevenson, B. L. Turner, R. A. G. Viani, W. Wanek, B. Zeller, Convergence of soil nitrogen isotopes across global climate gradients. *Sci. Rep.*, **5,** 8280 (2015).

11. J. P. Sena-Souza, B. Z. Houlton, L. A. Martinelli, G. B. Nardoto, Reconstructing continental‐scale variation in soil δ^15^N: a machine learning approach in South America. *Ecosphere*, **11**, e03223 (2020).

12. J. M. Craine, E. N. J. Brookshire, M. D. Cramer, N. J. Hasselquist, K. Koba, E. Marin-Spiotta, L. Wang, Ecological interpretations of nitrogen isotope ratios of terrestrial plants and soils. *Plant Soil*, **396**, 1-26 (2015).

13. R. Amundson, A. T. Austin, E. A. G. Schuur, K. Yoo, V. Matzek, C. Kendall, A. Uebersax, D. Brenner, W. T. Baisden, Global patterns of the isotopic composition of soil and plant nitrogen. *Global Biogeochem. Cycles*, **17**, 1031 (2003).

14. T. R. A. Denk, J. Mohn, C. Decock, D. Lewicka-Szczebak, E. Harris, K. Butterbach-Bahl, R. Kiese, B. Wolf, The nitrogen cycle: A review of isotope effects and isotope modeling approaches. *Soil Biol. Biochem.*, **105,** 121-137 (2017).

15. M. G. Hastings, J. C. Jarvis, E. J. Steig, Anthropogenic impacts on nitrogen isotopes of ice-core nitrate. *Science,* **324**, 1288 (2009).

16. L. Geng, B. Alexander, J. Cole-Dai, E. J. Steig, J. Savarino, E. D. Sofen, A. J. Schauer, Nitrogen isotopes in ice core nitrate linked to anthropogenic atmospheric acidity change. *Proc. Natl. Acad. Sci. U. S. A.,* **111**, 5808-5812 (2014).

17. K. Allen, J. B. Fisher, R. P. Phillips, J. S. Powers, E. R. Brzostek, Modeling the carbon cost of plant nitrogen and phosphorus uptake across temperate and tropical Forests. *Front. For. Glob. Change,* **3**, 43 (2020).

18. S. Running, Q. Mu, M. Zhao, M. Sips. MYD17A2H-MODIS/Aqua gross primary productivity 8-day L4 global 500m SIN grid v061. *NASA EOSDIS Land Processes DAAC* (2021). https://doi.org/10.5067/MODIS/MYD17A2H.061.

19. T. F Keenan, I. C. Prentice, J. G. Canadell, C. A. Williams, H. Wang, M. Raupach, G. J. Collatz, Recent pause in the growth rate of atmospheric CO_2_ due to enhanced terrestrial carbon uptake. *Nat. Commun.*, **7,** 13428 (2016).

20. M. Jung, M. Reichstein, C. R. Schwalm, C. Huntingford, S. Sitch, A. Ahlström, A. Arneth, G. Camps-Valls, P. Ciais, P. Friedlingstein, F. Gans, K. Ichii, A. K. Jain, E. Kato, D. Papale, B. Poulter, B. Raduly, C. Rödenbeck, G. Tramontana, N. Viovy, Y.-P. Wang, U. Weber, S. Zaehle, N. Zeng*.* Compensatory water effects link yearly global land CO_2_ sink changes to temperature. Nature*,* **541**, 516-520 (2017).

21. A. A. Bloom, J. Exbrayat, I. R. van der Velde, L. Feng, M. Williams, The decadal state of the terrestrial carbon cycle: Global retrievals of terrestrial carbon allocation, pools, and residence times. *Proc. Natl. Acad. Sci. U. S. A.,* **113**, 1285-1290 (2016).

22. Á. Moreno-Martínez, G. Camps-Valls, J. Kattge, N. Robinson, M. Reichstein, P. van Bodegom, K. Kramer, J. H. C. Cornelissen, P. Reich, M. Bahn, Ü. Niinemets, J. Peñuelas, J. M. Craine, B. E. L. Cerabolini, V. Minden, D. C. Laughlin, L. Sack, B. Allred, C. Baraloto, C. Byun, N. A. Soudzilovskaia, S. W. Running, A methodology to derive global maps of leaf traits using remote sensing and climate data. *Remote Sens. Environ.,* **218**, 69-88 (2018).

23. Y. Sun, D. S. Goll, J. Chang, P. Ciais, B. Guenet, J. Helfenstein, Y. Huang, R. Lauerwald, F. Maignan, V. Naipal, Y. Wang, H. Yang, H. Zhang. Global evaluation of the nutrient-enabled version of the land surface model ORCHIDEE-CNP v1.2 (r5986), *Geosci. Model Dev.*, **14**, 1987–2010 (2021).

24. Y.-P. Wang, R. M. Law, B. Pak, A global model of carbon, nitrogen and phosphorus cycles for the terrestrial biosphere. *Biogeosciences*, **7**, 2261-2282 (2010).

25. M. Deng, L. Liu, L. Jiang, W. Liu, X. Wang, S. Li, S. Yang, B. Wang. Ecosystem scale trade-off in nitrogen acquisition pathways. *Nat. Ecol. Evol.,* **2**, 1724-1734 (2018).

26. H. Chen, I. C. Covert, S. M. Lundberg, S.-I. Lee. Algorithms to estimate Shapley value feature attributions. *Nat. Mach. Intell.* **5**, 590–601 (2023).

27. J. M. Craine, A. J. Elmore, M. P. M. Aidar, M. Bustamante, T. E. Dawson, E. A. Hobbie, A. Kahmen, M. C. Mack, K. K. McLauchlan, A. Michelsen, G. B. Nardoto, L. H. Pardo, J. Peñuelas, P. B. Reich, E. A. G. Schuur, W. D. Stock, P. H. Templer, R. A. Virginia, J. M. Welker, I. J. Wright, Global patterns of foliar nitrogen isotopes and their relationships with climate, mycorrhizal fungi, foliar nutrient concentrations, and nitrogen availability. *New Phytol.,* **183**, 980-992 (2009).

28. J. Peng, Y.-P., Wang, B. Z. Houlton, L. Dan, B. Pak, X. Tang, Global carbon sequestration is highly sensitive to model‐based formulations of nitrogen fixation. *Global Biogeochem. Cycles*, **34**, e2019GB006296 (2020).

29. G. A. Gurmesa, A. Wang, S. Li, S. Peng, W. de Vries, P. Gundersen, P. Ciais, O. L. Phillips, E. A. Hobbie, W. Zhu, K. Nadelhoffer, Y. Xi, E. Bai, T. Sun, D. Chen, W. Zhou, Y. Zhang, Y. Guo, J. Zhu, L. Duan, D. Li, K. Koba, E. Du, G. Zhou, X. Han, S. Han, Y. Fang, Retention of deposited ammonium and nitrate and its impact on the global forest carbon sink. *Nat. Commun.,* **13**, 880 (2022).

30. E. Bai, B. Z. Houlton, Coupled isotopic and process-based modeling of gaseous nitrogen losses from tropical rain forests. Global Biogeochem. Cycles, **23**, GB2011 (2009).

31. T. Davies‐Barnard, P. Friedlingstein, The global distribution of biological nitrogen fixation in terrestrial natural ecosystems. *Global Biogeochem. Cycles,* **34,** e2019GB006387 (2020).

33. W. Shangguan, Y. Dai, Q. Duan, B. Liu, H. Yuan, A global soil data set for earth system modeling. *J. Adv. Model. Earth Syst.*, **6**, 249-263 (2014).

34. B. S. Steidinger, T. W. Crowther, J. Liang, M. E. Van Nuland, G. D. A. Werner, P. B. Reich, G. J. Nabuurs, S. de-Miguel, M. Zhou, N. Picard, B. Herault, X. Zhao, C. Zhang, D. Routh, K. G. Peay, GFBI consortium, Climatic controls of decomposition drive the global biogeography of forest-tree symbioses. *Nature,* **569**, 404-408 (2019).

35. H. Tian, J. Yang, C. Lu, R. Xu, J. G. Canadell, R. B. Jackson, A. Arneth, J. Chang, G. Chen, P. Ciais, S. Gerber, A. Ito, Y. Huang, F. Joos, S. Lienert, P. Messina, S. Olin, S. Pan, C. Peng, E. Saikawa, R. L. Thompson, N. Vuichard, W. Winiwarter, S. Zaehle, B. Zhang, K. Zhang, Q. Zhu, The global N_2_O model intercomparison project. *Bull. Am. Meteorol. Soc.*, **99**, 1231-1251 (2018).

36. B. Smith, D. Wårlind, A. Arneth, T. Hickler, P. Leadley, J. Siltberg, S. Zaehle, Implications of incorporating N cycling and N limitations on primary production in an individual-based dynamic vegetation model. *Biogeosciences*, **11**, 2027-2054 (2014).

37. D. S. Goll, A. J. Winkler, T. Raddatz, N. Dong, I. C. Prentice, P. Ciais, V. Brovkin, Carbon–nitrogen interactions in idealized simulations with JSBACH (version 3.10). *Geosci. Model Dev.*, **10**, 2009-2030 (2017).

38. M. Inatomi, A. Ito, K. Ishijima, S. Murayama, Greenhouse gas budget of a cool-temperate deciduous broad-leaved forest in Japan estimated using a process-based model. *Ecosystems*, **13**, 472-483 (2010).

39. A. J. Wiltshire, E. J. Burke, S. E. Chadburn, C. D. Jones, P. M. Cox, T. Davies-Barnard, P. Friedlingstein, A. B. Harper, S. Liddicoat, S. Sitch, S. Zaehle, JULES-CN: a coupled terrestrial carbon–nitrogen scheme (JULES vn5.1). Geosc. Model Dev., **14**, 2161-2186 (2021).

40. D. Robinson, δ^15^N as an integrator of the nitrogen cycle. *Trends Ecol. Evol.*, **16**, 153-162 (2001).

41. E. Bai, B. Z. Houlton, Y.-P. Wang, Isotopic identification of nitrogen hotspots across natural terrestrial ecosystems, *Biogeosciences*, **9**, 3287–3304, 2012.

42. G. M. Lovett, S. E. Lindberg, Atmospheric deposition and canopy interactions of nitrogen in forests. *Can. J. Forest Res.*. **23**: 1603-1616 (1993).

43. R. L. Boyce, A. J. Friedland, C. P. Chamberlain, S. R. Poulson, Direct canopy nitrogen uptake from ^15^N-labeled wet deposition by mature red spruce. *Can. J. Forest Res.*, **26**: 1539–1547 (1996).

44. B. Muller, B. Touraine, H. Rennenberg, Interaction between atmospheric and pedospheric nitrogen nutrition in spruce (*Picea abies* L. Karst) seedlings. *Plant Cell Environ.*, **19**, 345-355 (1996).

45. E. J. Wilson, C. Tiley, Foliar uptake of wet-deposited nitrogen by norway spruce: an experiment using ^15^N, *Atmos. Environ.*, **32**, 513-518 (1998)

46. M. Ammann, R. Siegwolf, F. Pichlmayer, M. Suter, M. Saurer, C. Brunold, Estimating the uptake of traffic-derived NO_2_ from ^15^N abundance in Norway spruce needles. *Oecologia*, **118**, 124-131 (1999).

47. Schulze, E.-D. Carbon and nitrogen cycling in European forest ecosystems. *Ecol. Stud*. **142**: 1-500 (2000).

48. R. T. W. Siegwolf, R. Matyssek, M. Saurer, S. Maurer, M. S. Günthardt-Goerg, P. Schmutz, J. B. Bucher, Stable isotope analysis reveals differential effects of soil nitrogen and nitrogen dioxide on the water use efficiency in hybrid poplar leaves. *New Phytol.* **149**, 233–246 (2001).

49. T. Tomaszewski, R. L. Boyce, H. Sievering, Canopy uptake of atmospheric nitrogen and new growth nitrogen requirement at a Colorado subalpine forest. *Can. J. Forest Res.*, **33**, 2221-2227 (2003).

50. D. M. Vallano, J. P. Sparks, Quantifying foliar uptake of gaseous nitrogen dioxide using enriched foliar δ^15^N values. *New Phytol.*, **177**, 946-955 (2008).

51. H. Sievering, T. Tomaszewski, J. Torizzo, Canopy uptake of atmospheric N deposition at a conifer forest: Part I–Canopy N budget, photosynthetic efficiency and net ecosystem exchange. *Tellus B Chem. Phys. Meteorol.*, **59**, 483-492 (2007).

52. E. Gaige, D. B. Dail, D. Y. Hollinger, E. A. Davidson, I. J. Fernandez, H. Sievering, A. White, W. Halteman,  Changes in canopy processes following whole-forest canopy nitrogen fertilization of a mature Spruce-Hemlock forest. *Ecosystems*, **10**, 1133-1147 (2007).

53. E. Wortman, T. Tomaszewski, P. Waldner, P. Schleppi, A. Thimonier, W. Eugster, N. Buchmann, H. Sievering, Atmospheric nitrogen deposition and canopy retention influences on photosynthetic performance at two high nitrogen deposition Swiss forests, *Tellus B Chem. Phys. Meteorol.*, **64**, 1 (2012).

54. M. Uscola, P. Villar-Salvador, J. Oliet, C. R. Warren, Foliar absorption and root translocation of nitrogen from different chemical forms in seedlings of two Mediterranean trees, *Environ. Exp. Bot.*, **104**, 34-43 (2014).

55. M. T. Schwarz, S. Bischoff, S. Blaser, S. Boch, B. Schmitt, L. Thieme, M. Fischer, B. Michalzik, E.-D. Schulze, J. Siemens, W. Wilcke, More efficient aboveground nitrogen use in more diverse Central European forest canopies. *Forest Ecol. Manag.*, **313**, 274–282 (2014).

56. D. Houle, C. Marty, L. Duchesne, Response of canopy nitrogen uptake to a rapid decrease in bulk nitrate deposition in two eastern Canadian boreal forests. *Oecologia*, **177**, 29–37 (2015).

57. R.K.F. Nair, M.P. Perks, A. Weatherall, E.M. Baggs, M. Mencuccini, Does canopy nitrogen uptake enhance carbon sequestration by trees? *Global Change Biol.*, **22**, 875-888 (2016).

58. I. Bourgeois, J.-C. Clément, N. Caillon, J. Savarino, Foliar uptake of atmospheric nitrate by two dominant subalpine plants: insights from *in situ* triple-isotope analysis. *New Phytol.*, **223**, 1784-1794 (2019).

59. T. Liu, P. Mao, L. Shi, N. Eisenhauer, S. Liu, X. Wang, X. He, Z. Wang, W. Zhang, Z. Liu, L. Zhou, Y. Shao, S. Fu, Forest canopy maintains the soil community composition under elevated nitrogen deposition. *Soil Biol. Biochem.*, **143**, 107733 (2020).

60. D. Ferraretto, R. Nair, N.W. Shah, D. Reay, M. Mencuccini, M. Spencer, K.V. Heal Forest canopy nitrogen uptake can supply entire foliar demand, *Funct. Ecol.*, **36**, 933-949 (2022).

61. P. M. Vitousek, D. N. L. Menge, S. C. Reed, C. C. Cleveland, Biological nitrogen fixation: rates, patterns and ecological controls in terrestrial ecosystems. *Philos. Trans. R. Soc. Lond. B, Biol. Sci.,* **368**, 20130119 (2013).

**
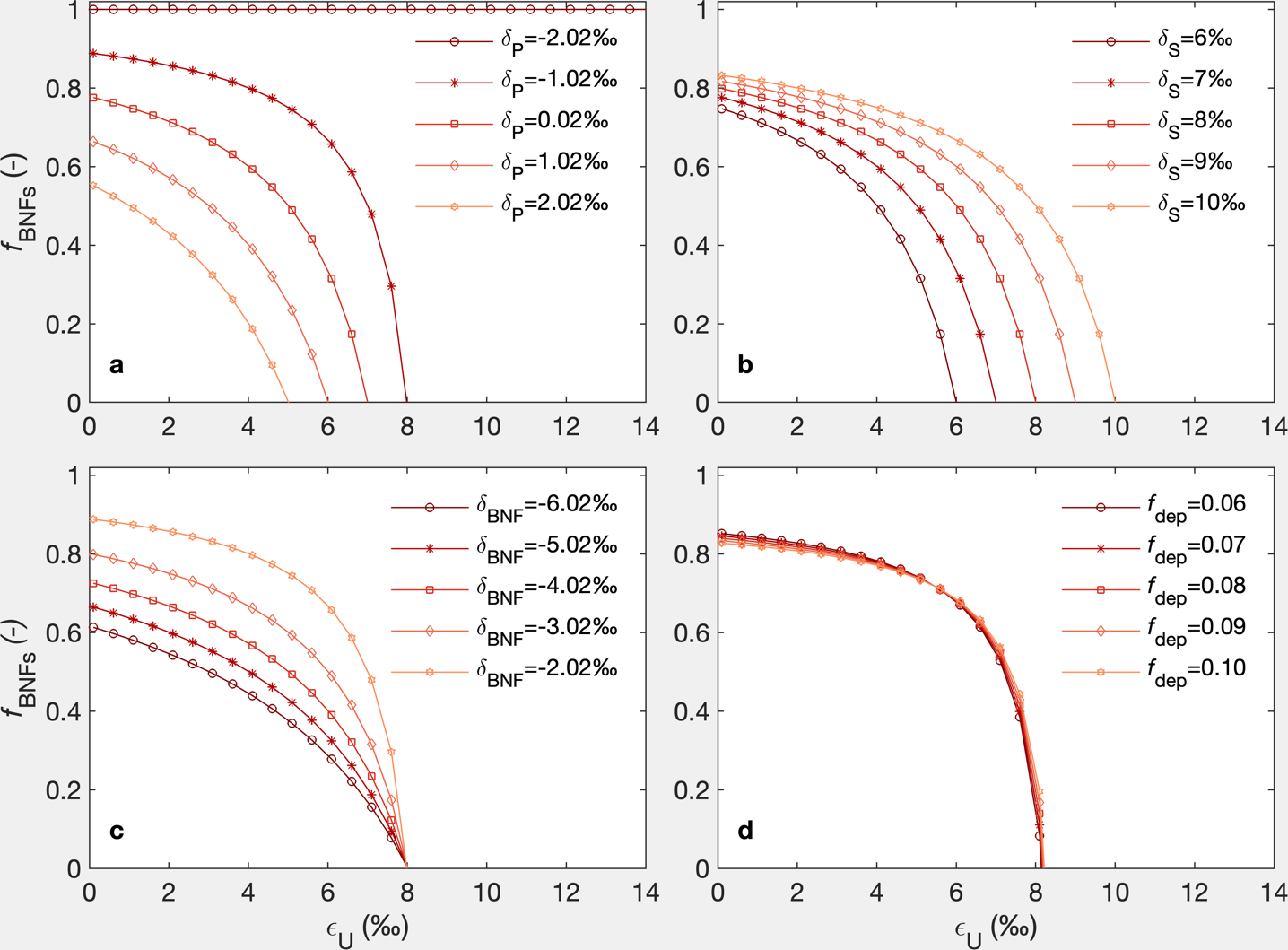
**

**Fig. S1.** Sensitivity of the theoretical negative relationship between the fraction of symbiotic biological nitrogen fixation (BNF) in vegetation-external nitrogen (N) demand (f_BNFs_) and isotope fractionation of vegetation N uptake (ε_U_). (**a–d**), Sensitivity of the theoretical curve to the plant δ^15^N (δ_P_), soil δ^15^N (δ_S_), BNF δ^15^N (δ_BNF_), and the fraction of canopy uptake of N deposition f_DEPd_, respectively. In (**a**), the parameters are set as: δ_S_ = 7‰, δ_BNF_=-2.02‰, δ_DEP_=1.5‰ and f_DEPd_=0; In (**b**), the parameters are set as: δ_P_ = -1.02‰, δ_BNF_ =-2.02‰, δ_DEP_=1.5‰ and f_DEPd_=0; In (**c**), the parameters are set as: δ_S_ = 7‰, δ_P_=-1.02‰, δ_DEP_=1.5‰ and f_DEPd_=0. In (**d**), the parameters are set as: δ_S_ = 7‰, δ_P_=-1.02‰, δ_BNF_=-2.02‰, and δ_DEP_=1.5‰.


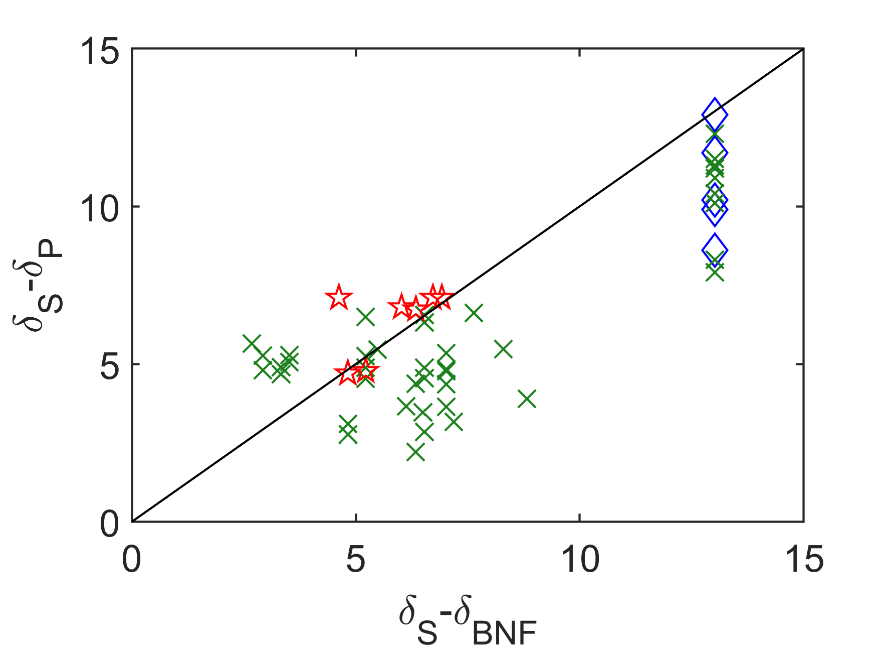


**Fig. S2.** Field observations of plant and soil δ^15^N for ecosystems with different microbial symbionts (N fixing bacteria (N-fixers), ecto-mycorrhizal (ECM) and arbuscular mycorrhizal (AM) fungi). The plants with AM, ECM, and N-fixers are represented by green crosses, red stars, and blue prismatic, respectively. The 1:1 line divides the whole panel into two zones: (1) ECM zone (top left region), and (2) non-ECM zone (bottom right region).


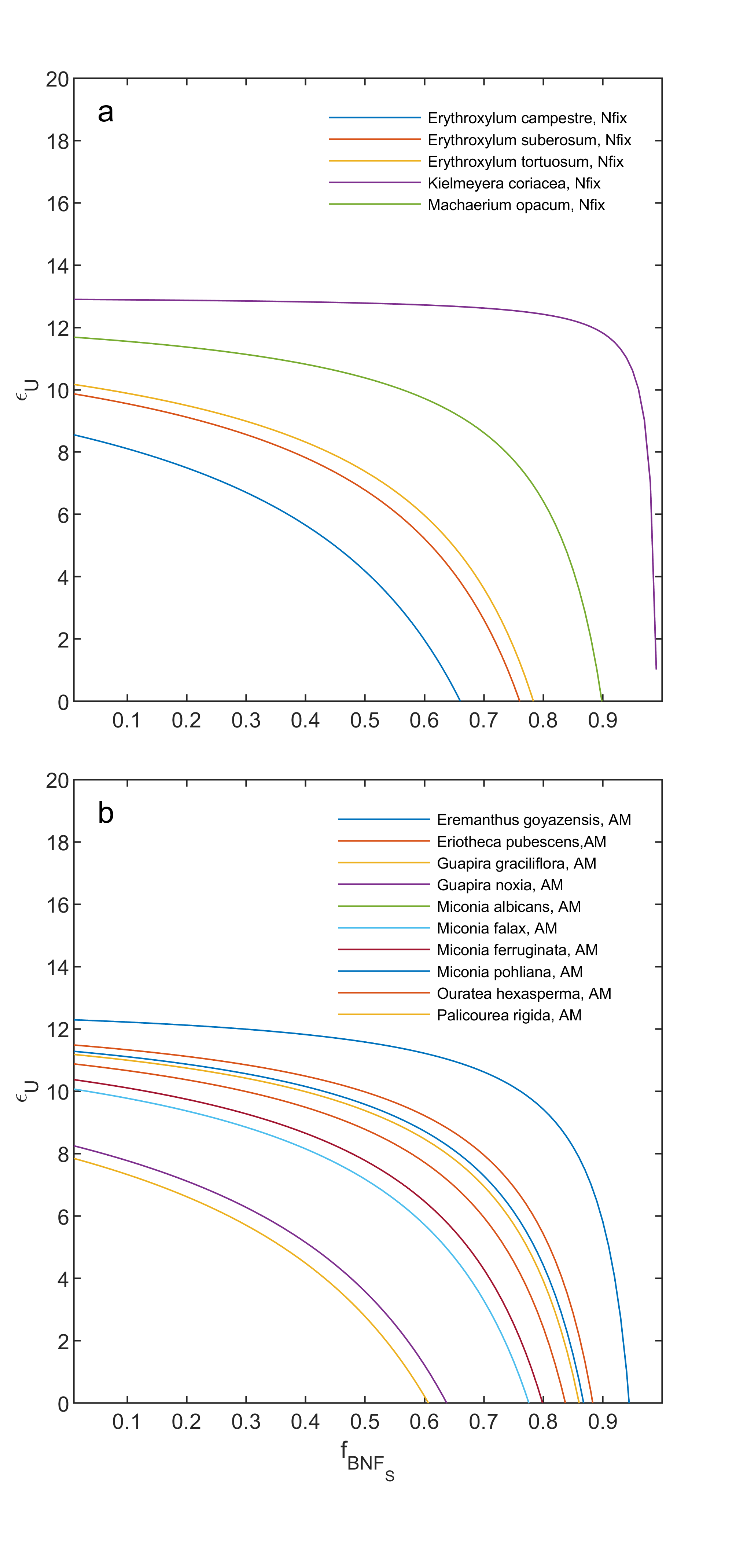


**Fig. S3.** Illustration of the theoretical negative relationship between f_BNFs_ and ε_U_ with field observations of plant and soil δ^15^N at Brazilian savannas. (**a**) and (**b**) illustrate the theoretical negative relationships for plants with nitrogen fixing bacteria and AM, respectively.


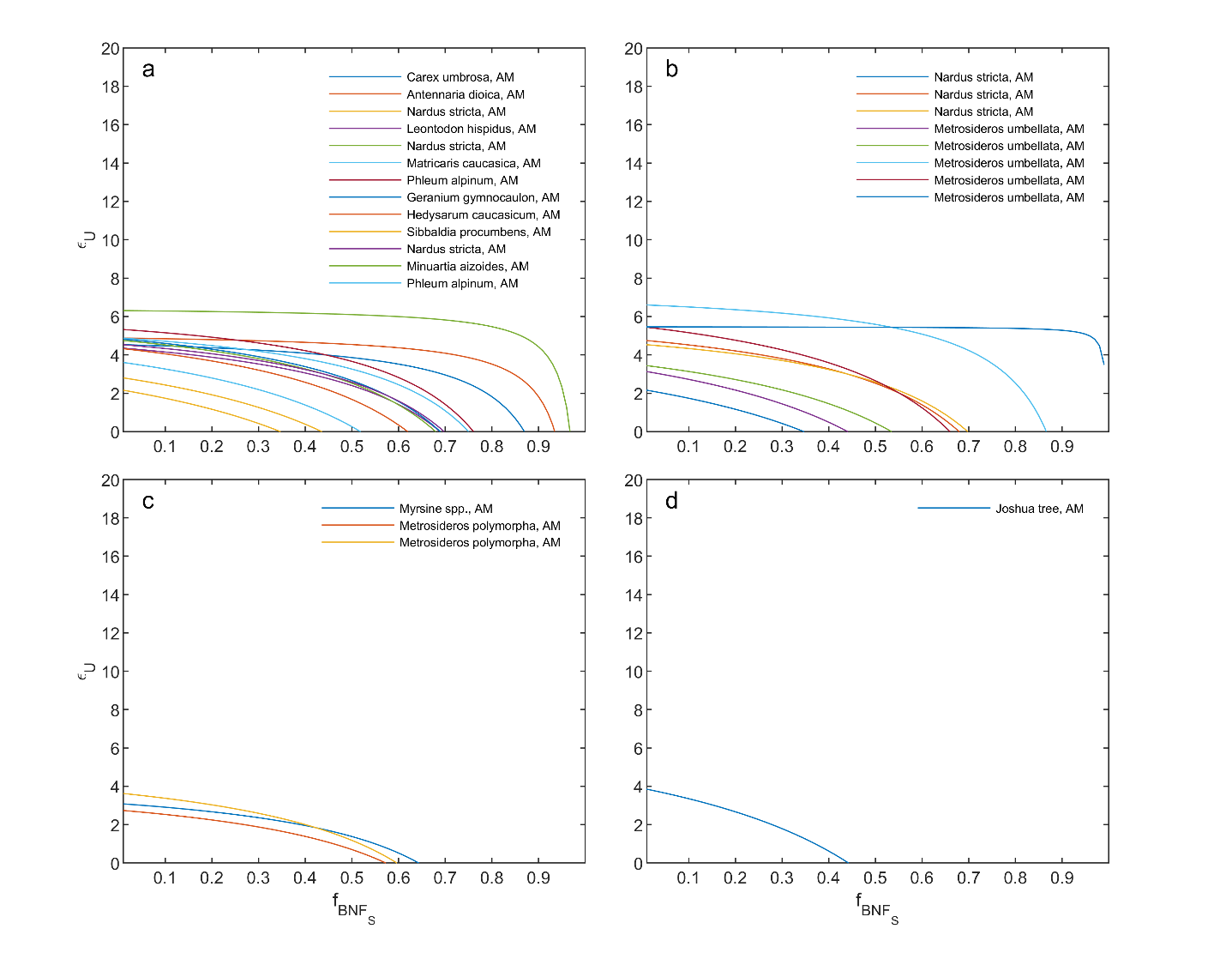


**Fig. S4.** Illustration of the theoretical negative relationship between f_BNFs_ and ε_U_ for AM-dominated plants at different sites. (**a**) Alpine of North Caucasus; (**b**) Hawi’i versus Alpine; (c) Hawi’i precipitation gradient; and (**d**) data in Amundson et al. [13].


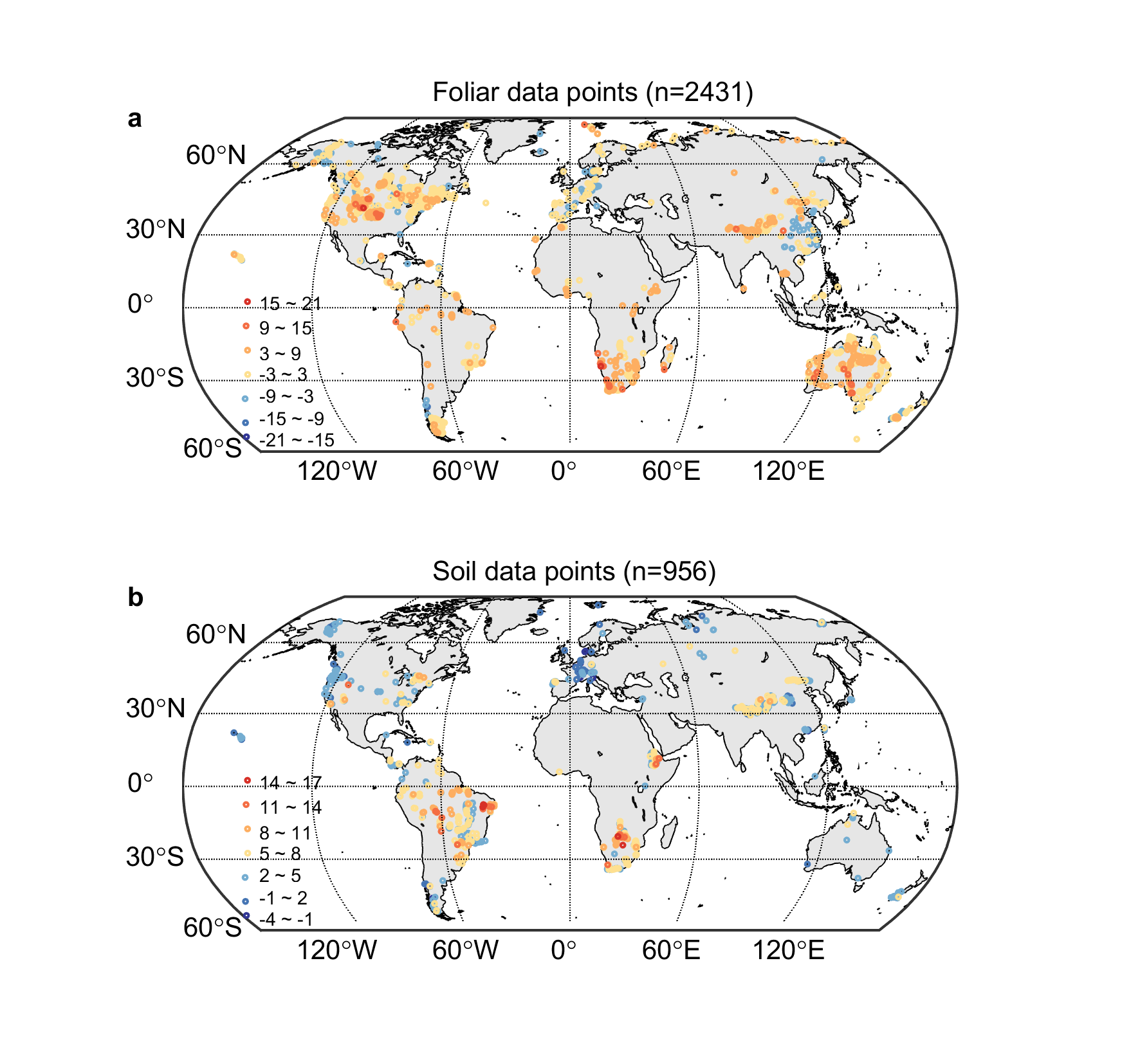


**Fig. S5.** Global distributions of sites for foliar and soil δ^15^N measurements. In (**a**), 38646 site-level foliar δ^15^N measurements are aggregated into 2431 0.1°×0.1° grid cells. In (**b**), 5887 site-level soil δ^15^N measurements are aggregated into 956 0.1°×0.1° grid cells. Different colors indicate the magnitudes of δ^15^N (Unit: ‰).


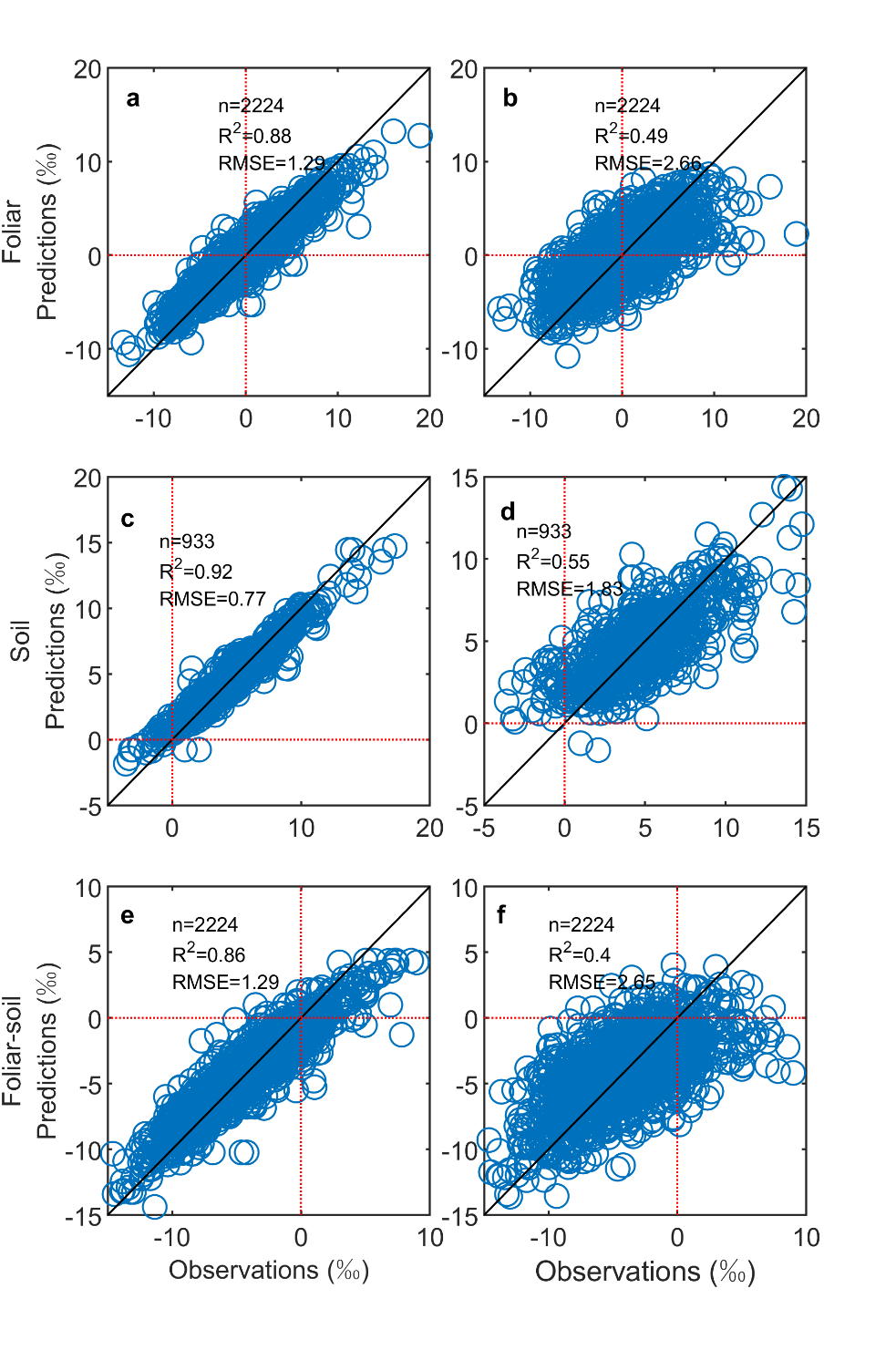


**Fig. S6.** Training and Testing (Out-of-Bag, OOB) of Random Forest (RF) models. (**a**) and (**b**) are training and testing of RF model for plant δ^15^N (δ_P_); (**c**) and (**d**) are training and testing of RF model for soil δ^15^N (δ_S_); (**e**) and (**f**) are training and testing of RF model for foliar-soil δ^15^N (δ_P_-δ_S_).


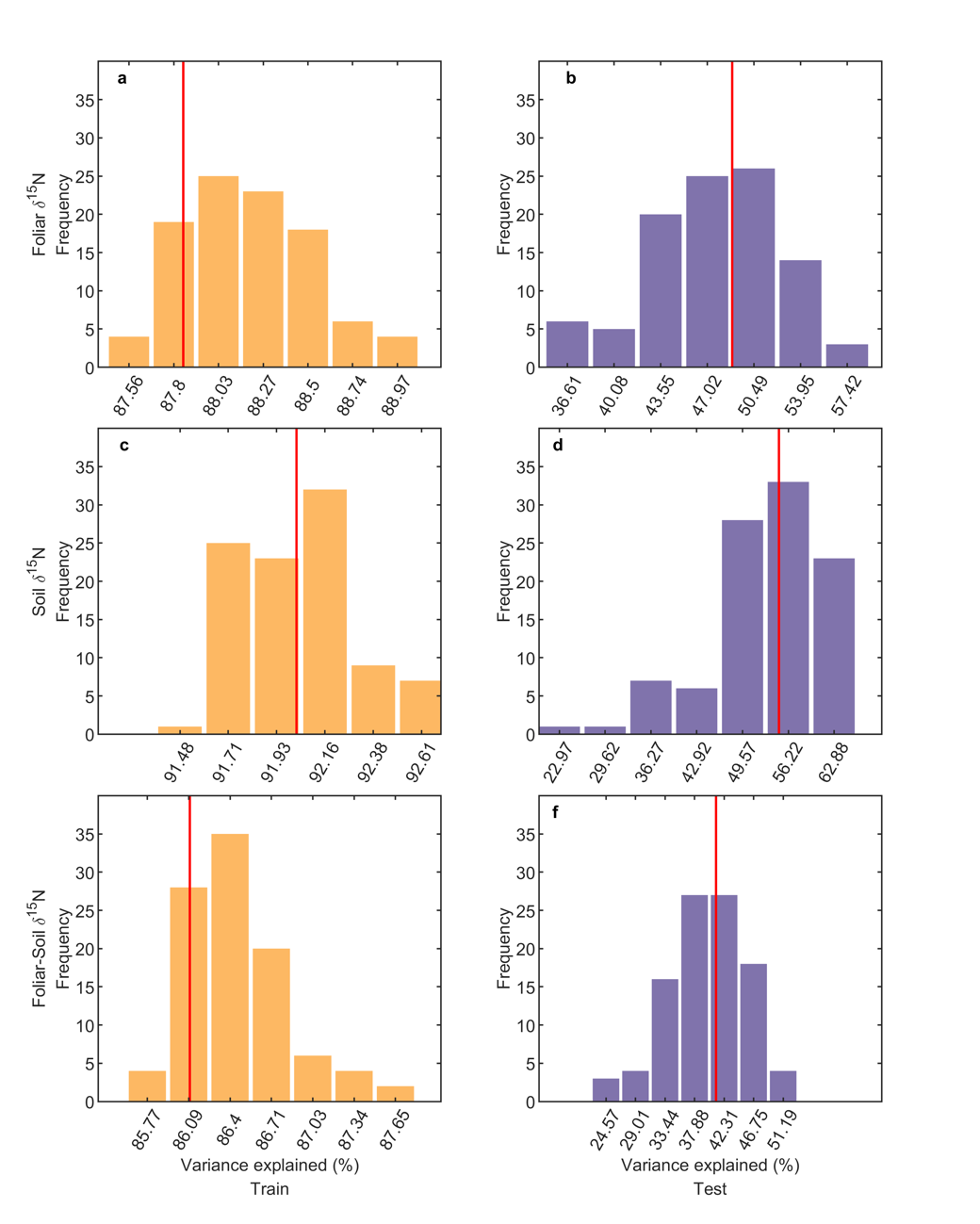


**Fig. S7.** Frequency distributions of the explained variance for the K-fold (K=10) cross validation of Random Forest (RF) models. (**a**), (**c**), and (**e**) are frequency distributions of the explained variance for the training of plant δ^15^N (δ_P_), soil δ^15^N (δ_S_), and plant-soil δ^15^N (δ_P_-δ_S_), respectively. (**b**), (**d**), and (**f**) are frequency distributions of the explained variance for the testing of δ_P_, δ_S_, and δ_P_-δ_S_, respectively, respectively. The red lines indicate the levels of explained variances of the original RF models, i.e., using all samples. The explained variance was represented by the coefficient of determination.


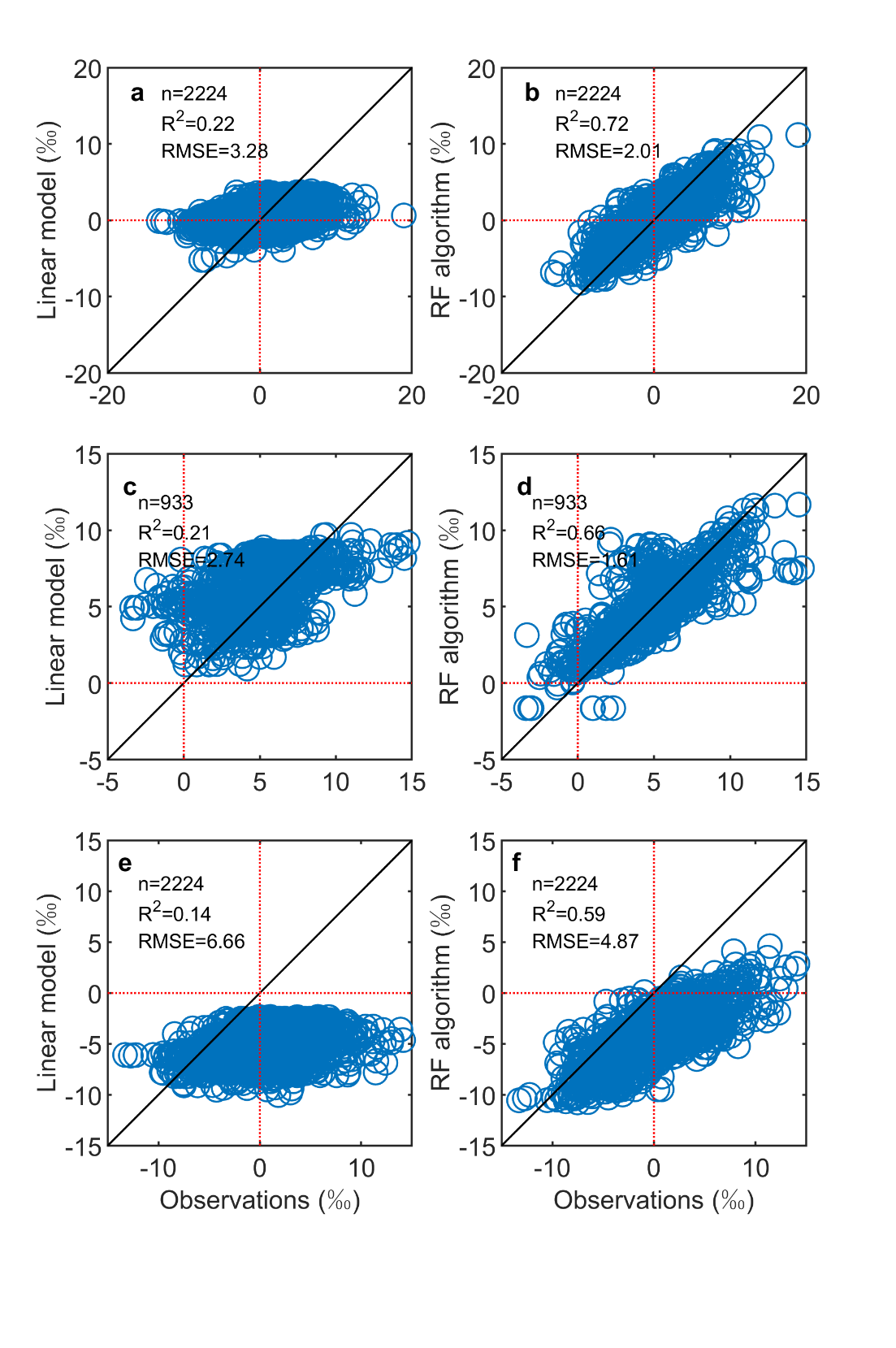


**Fig. S8.** Comparison of validities of linear regression models by Amundson *et al.* [13] (left) with those by Random Forest (RF) models (right). (**a**), (**c**), and (**e**) show the validities of linear regression models for plant δ^15^N (δ_P_), soil δ^15^N (δ_S_), and plant-soil δ^15^N (δ_P_-δ_S_), respectively. (**b**), (**d**), and (**f**) show the validities of δ_P_, δ_S_, and δ_P_-δ_S_, respectively.

*
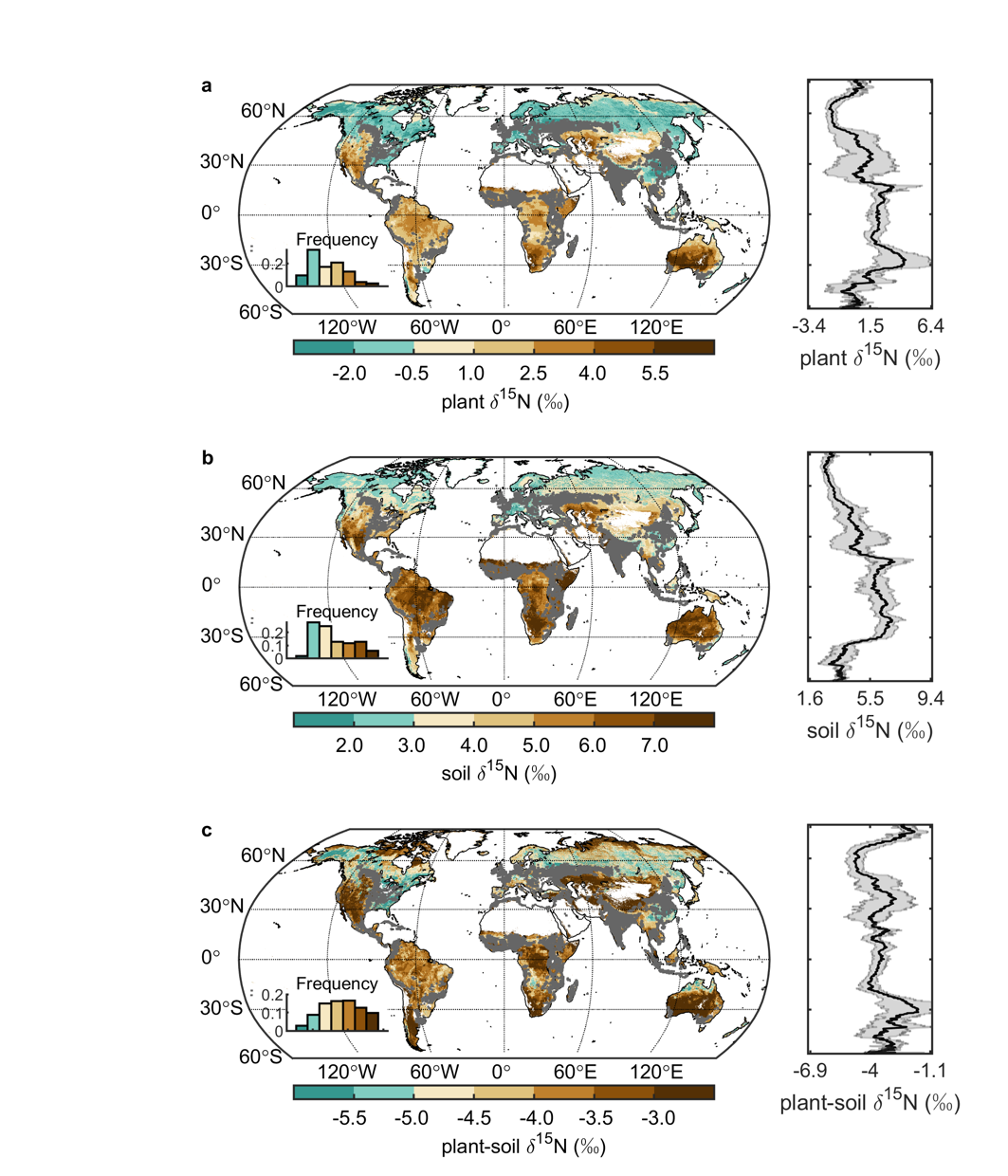
*

**Fig. S9.** Global maps of natural N isotope ratios (δ^15^N) upscaled from 38646 foliar δ^15^N and 5887 soil δ^15^N measurements using Random Forest models. (**a**–**c**), global maps of δ^15^N for plant (δ_P_), soil (δ_S_) and their difference (δ_P_ − δ_S_), respectively. The bottom left histograms present the frequency distributions of δ^15^N values. In the panels on the right of the maps, the black lines indicate the latitudinal means of δ^15^N, with their 95% confidence intervals represented by grey areas. The managed croplands and pasture were excluded in our analysis and are marked grey.


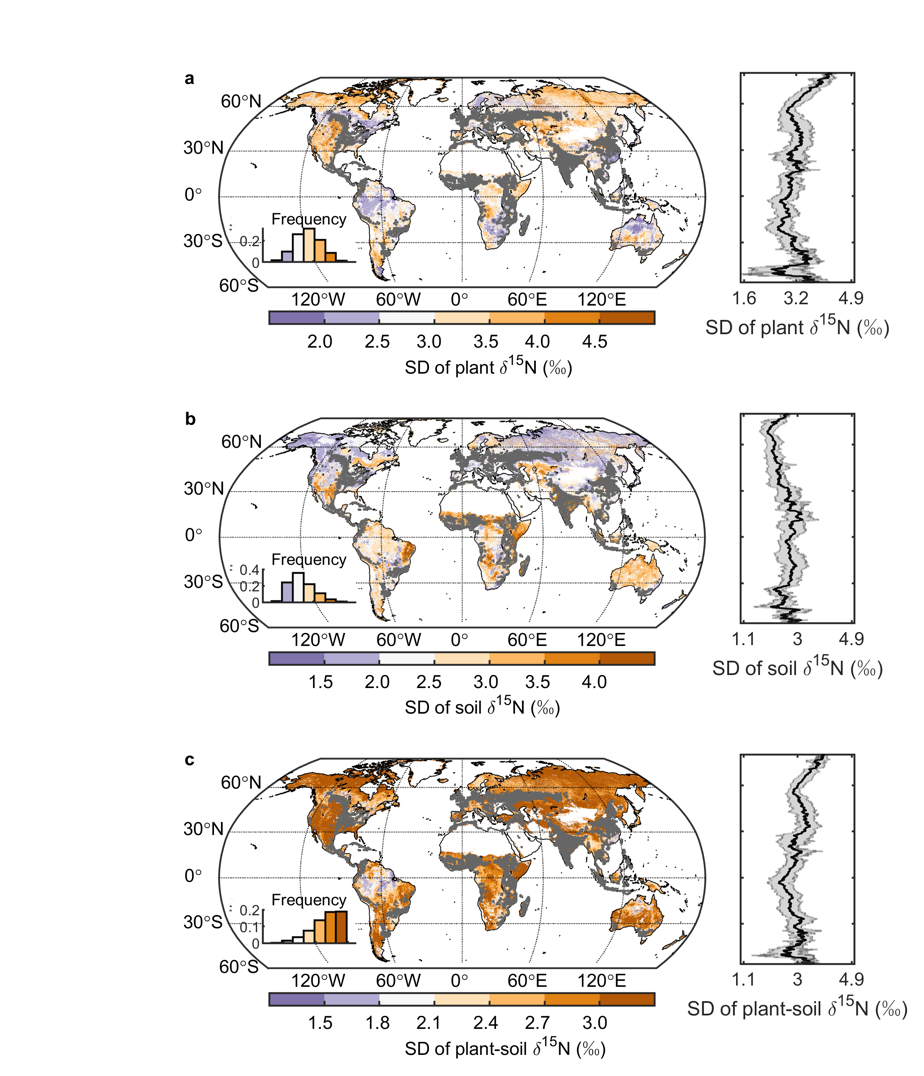


**Fig. S10.** Global maps of standard deviations (SDs) of δ^15^N produced by Random Forest (RF) models. (**a**–**c**), global maps of SDs for plant δ^15^N (δ_P_), soil δ^15^N (δ_S_), and plant-soil δ^15^N (δ_P_-δ_S_), respectively. The bottom left histograms present the frequency distributions of SD for δ^15^N values. In the panels on the right of the maps, the black lines indicate the latitudinal means, with their 95% confidence intervals represented by grey areas. The areas of managed croplands and pasture were excluded in our analysis and are represented by grey regions.


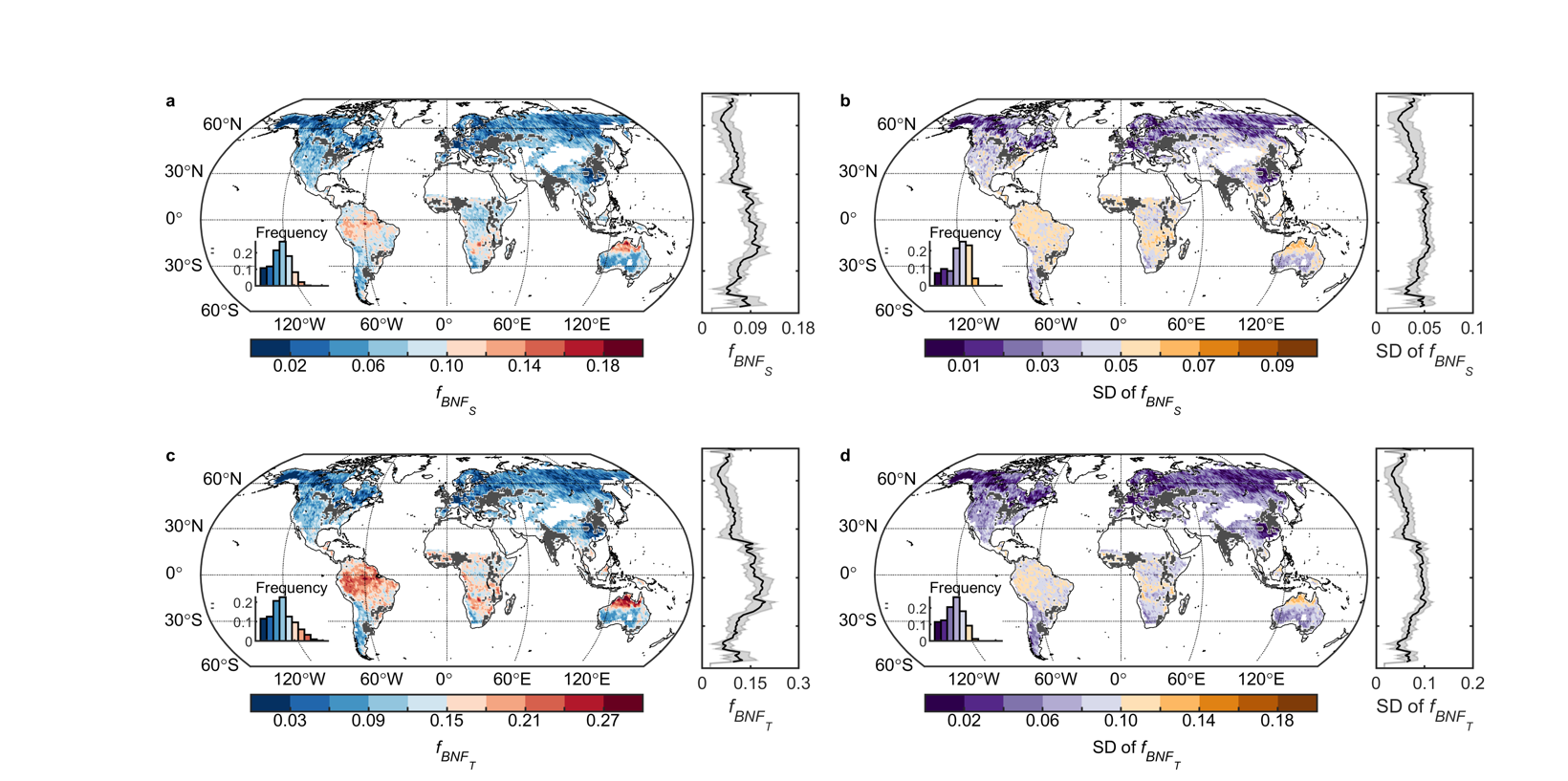


**Fig. S11.** Global maps of fractions of biological nitrogen fixation (BNF) in vegetation-external nitrogen (N) demands for natural terrestrial ecosystems derived by Bayesian approach. (**a**) and (**c**), Global maps of the fractions of symbiotic (*f*_BNFs_) and total BNF (${\text{f}_{\text{BNF}}}_{\text{T}}$) in vegetation-external N demands, respectively. (**b**) and (**d**), Global maps of standard deviations (SDs) of *f*_BNFs_ and ${\text{f}_{\text{BNF}}}_{\text{T}}$, respectively. The bottom left histograms present the frequency distributions of corresponding values. In the panels on the right of the map, the black lines indicate the latitudinal means while the grey areas indicate the 95% confidence intervals. The areas of managed croplands and pasture were excluded in our analysis, and are marked grey.

**
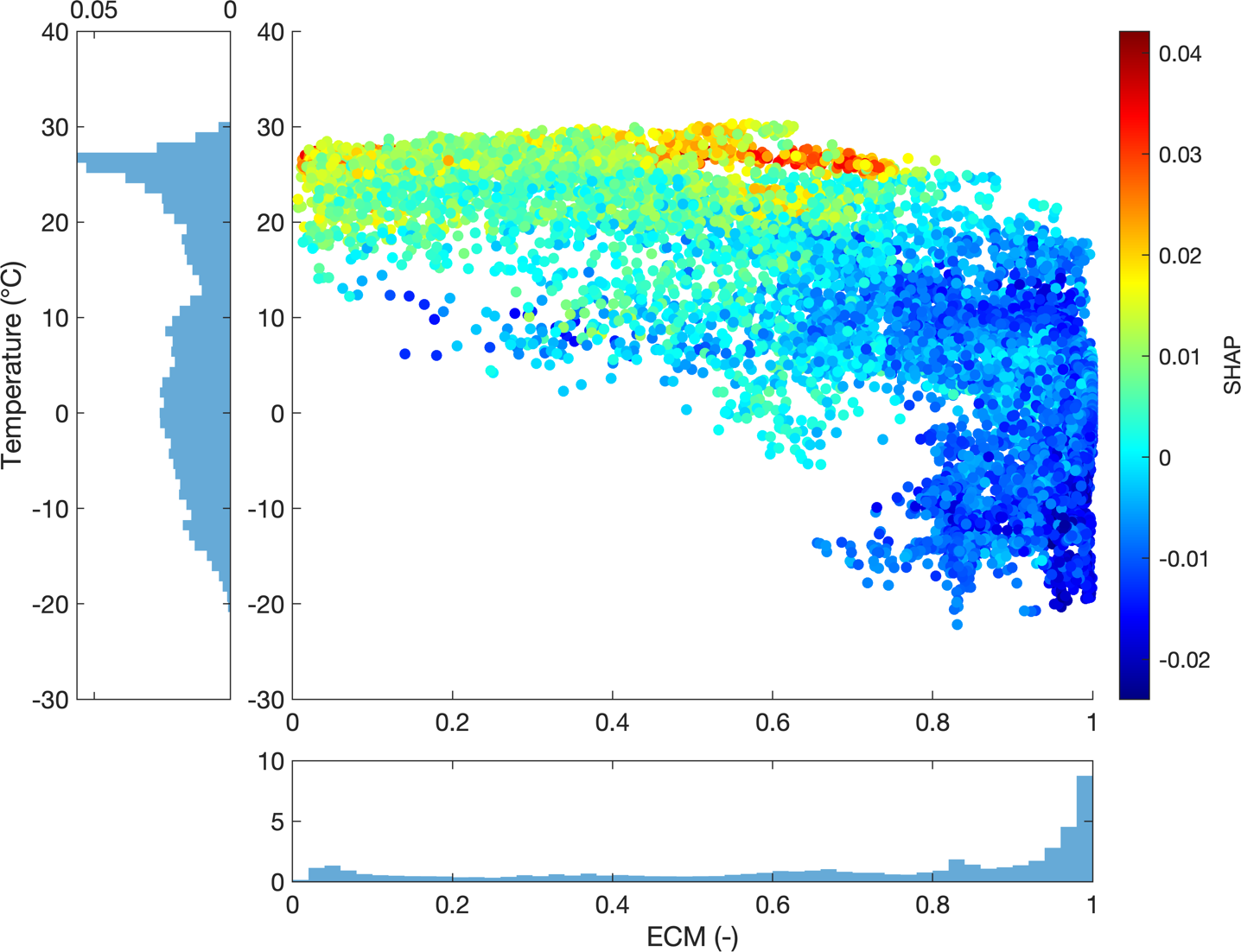
**

**Fig. S12.** Two-dimensional partial dependence plot of isotope-based f_BNFs_ versus temperature and natural abundance of ecto-mycorrhizal (ECM) fungi. The color of scatter points indicates the magnitudes of SHAP values. The histograms on the left and bottom are frequency distributions of temperature and ECM, respectively.


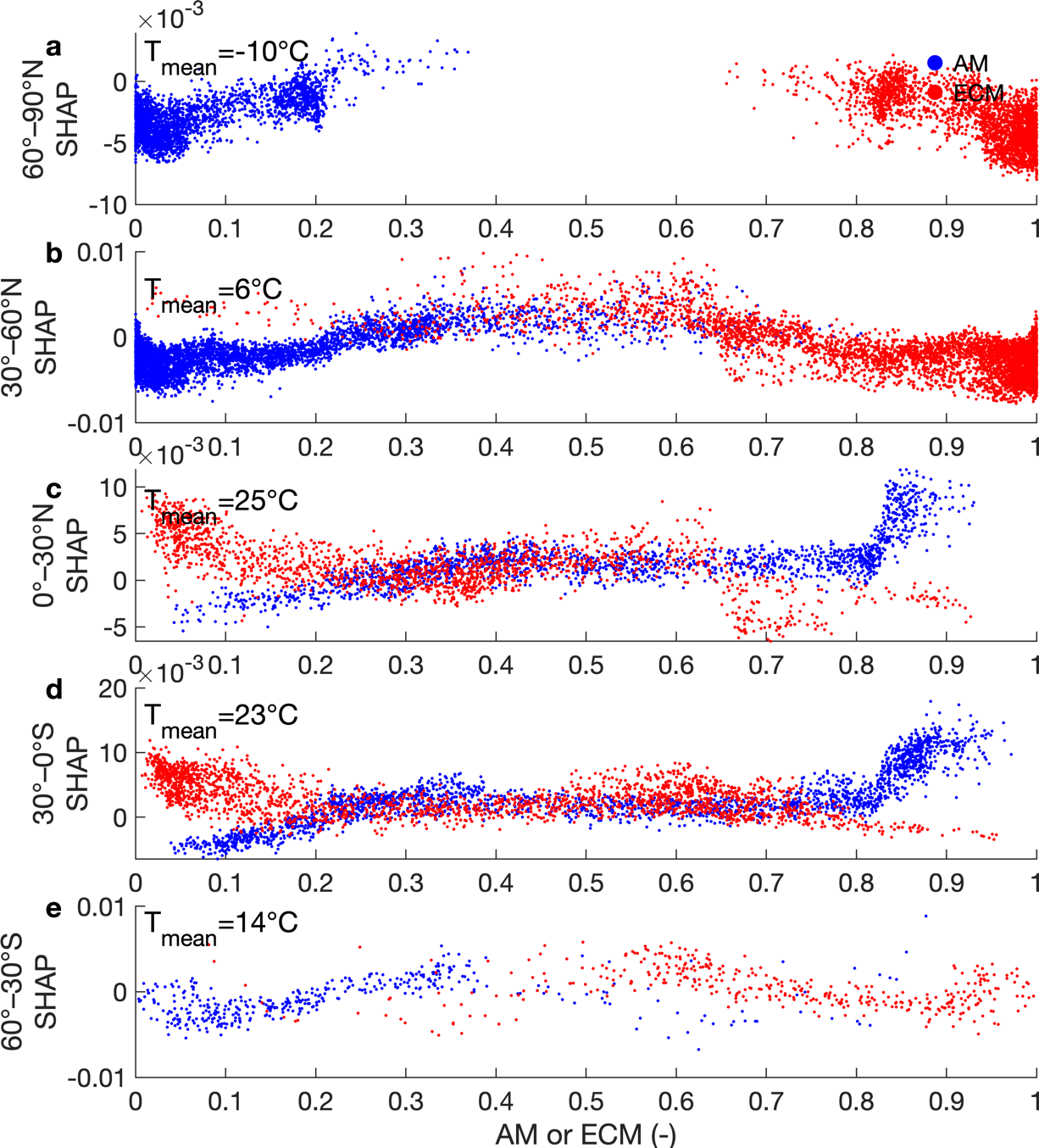


**Fig. S13.** Partial dependence plots between isotope-based *f*_BNFs_ and mycorrhizal fungi at different temperatures (corresponding to different latitudinal bands). (**a**), (**b**), (**c**), (**d**), and (**e**) are the partial dependence between isotope-based *f*_BNFs_ versus relative abundance of arbuscular mycorrhizal fungi (AM) and ectomycorrhizal fungi (ECM) in latitudinal bands of 60°–90°N, 30°–60°N, 0°–30°N, 30°–0°S, 60°–30°S, respectively.


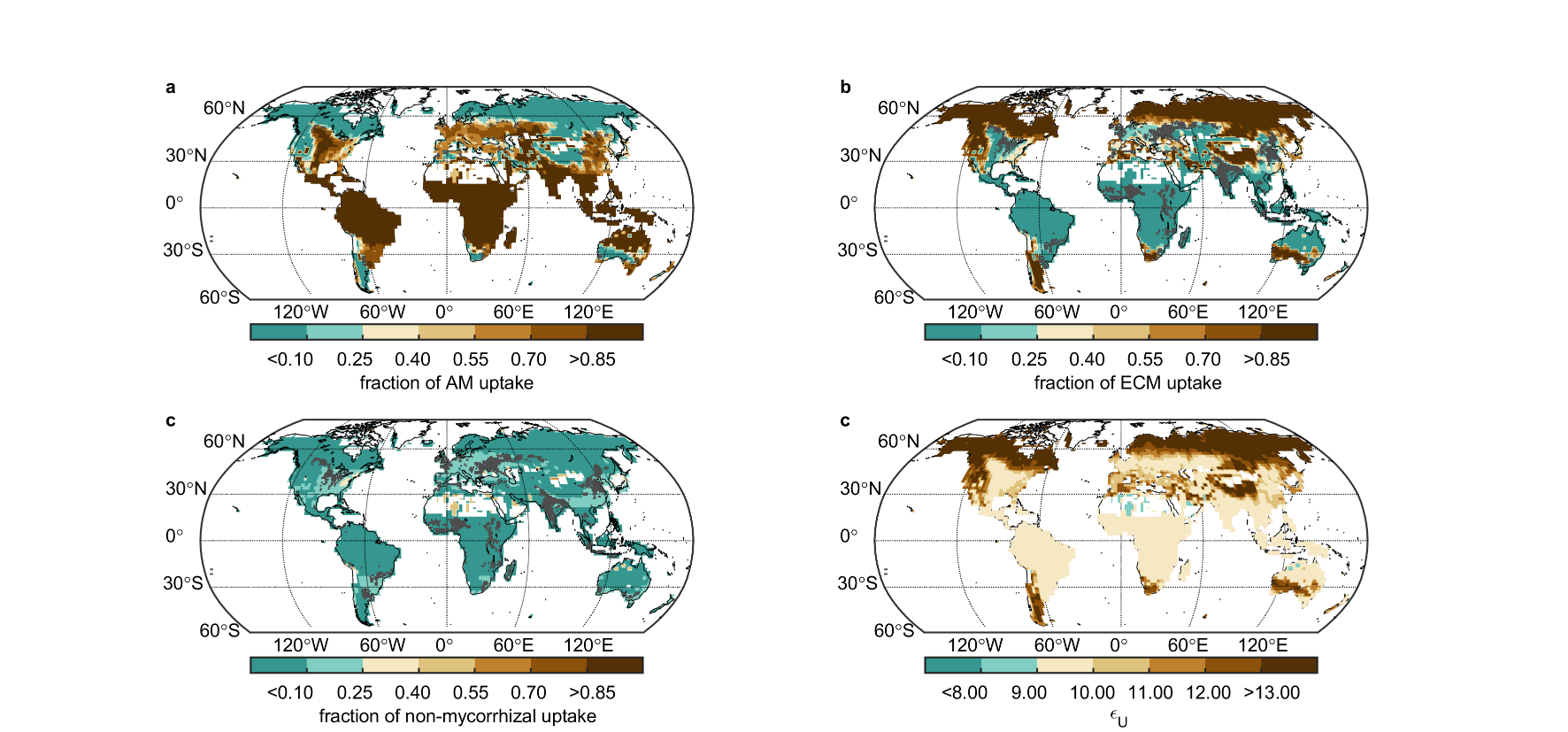


**Fig. S14.** Global maps of prior isotope fractionation of plant uptake (ε_U_). The prior ε_U_ is estimated as linear combinations of relative fractions of N uptake fluxes from arbuscular mycorrhizal fungi (AM), ecomycorrhizal fungi (ECM), and root pathways and their respective fractionation factors. (**a**), (**b**), and (**c**) are the relative fractions of N uptake fluxes from AM, ECM, and non-mycorrhizal roots, respectively, simulated by the Fixation and Uptake of Nitrogen (FUN v3.0) model [17]; (**d**) is the resulting prior global map of ε_U_. Note that the global maps of prior ε_U_ in this study are only for natural terrestrial ecosystems.


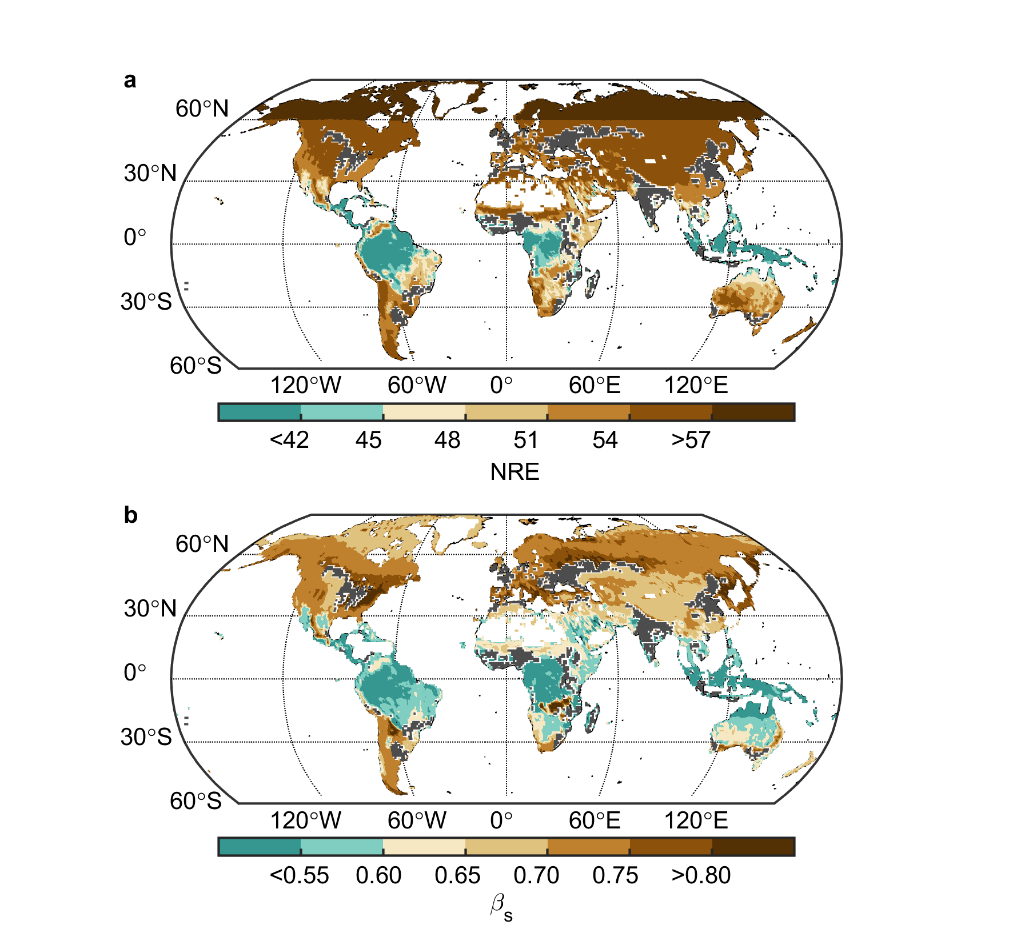


**Fig. S15.** Global maps of nitrogen resorption efficiency (NRE) and the fraction of symbiotic fixation in total BNF (β_S_). (**a**) Global map of N resorption efficiency (NRE, %); (**b**) Global map of the fraction of symbiotic over total BNF (β_S_). The areas of managed croplands and pastures were excluded in our analysis and are represented by grey regions.


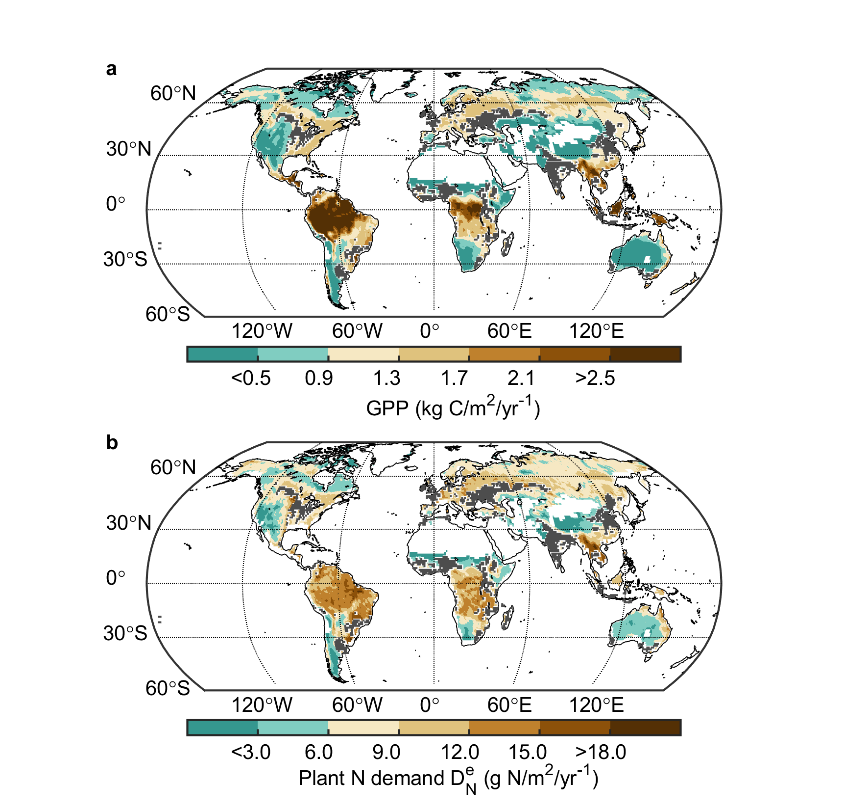


**Fig. S16.** Global map of the vegetation N demand from external sources estimated from gross primary production (GPP). (**a**) Global map of GPP from Keenan et al. [19]; (**b**) Global map of vegetation N demand from external sources. The areas of managed croplands and pastures were excluded in our analysis and are represented by grey regions.


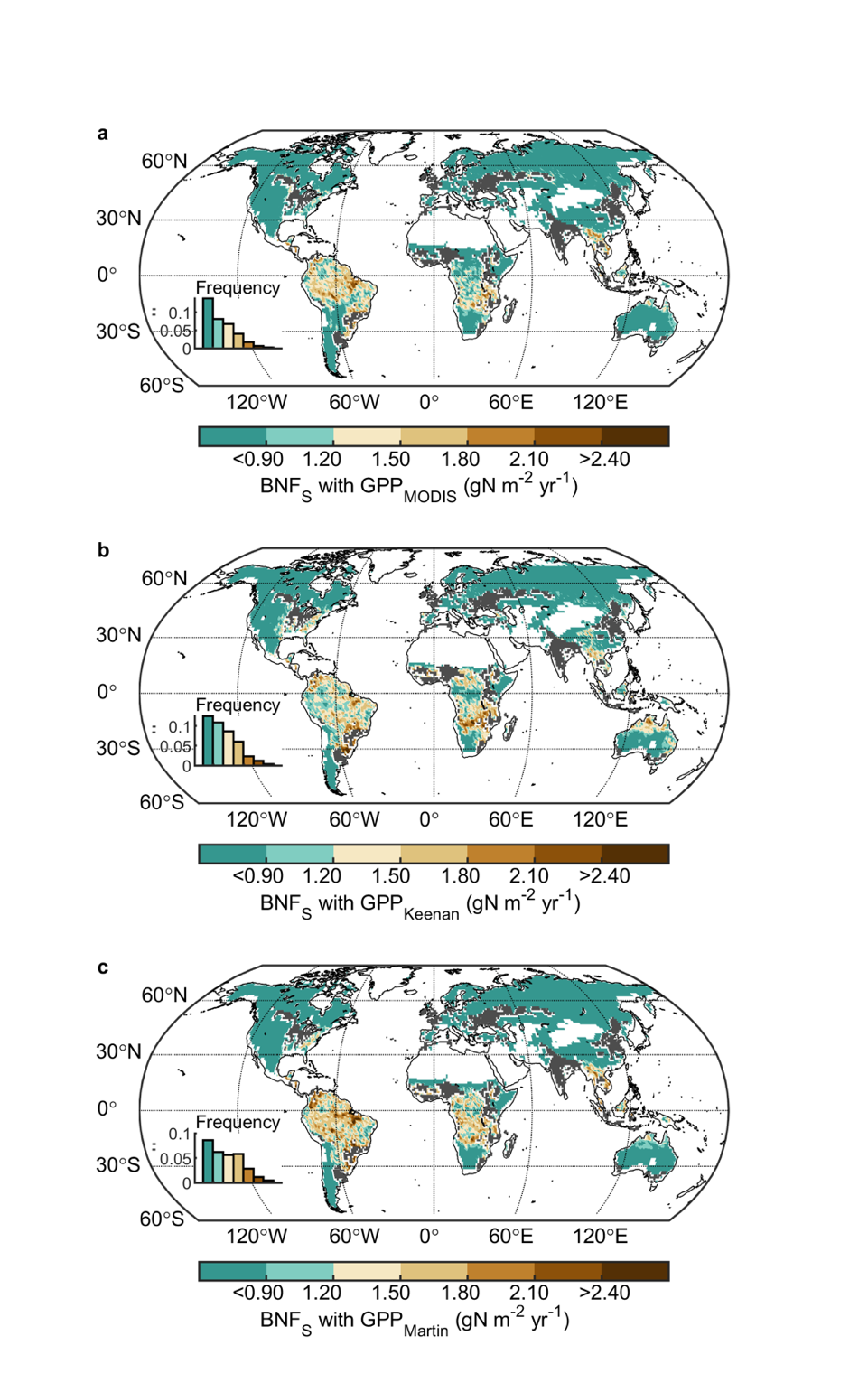


**Fig. S17.** Comparison of global maps of **symbiotic** BNF derived from three sources of gross primary production (GPP). (**a**), (**b**), and (**c**) are global maps of symbiotic BNF estimated with GPP from MODIS [18], Keenan et al. [19], and Jung et al. [20], respectively. The bottom left histograms present the frequency distributions of BNF estimates. The areas of managed croplands and pastures were excluded in our analysis and are represented by grey regions.

*
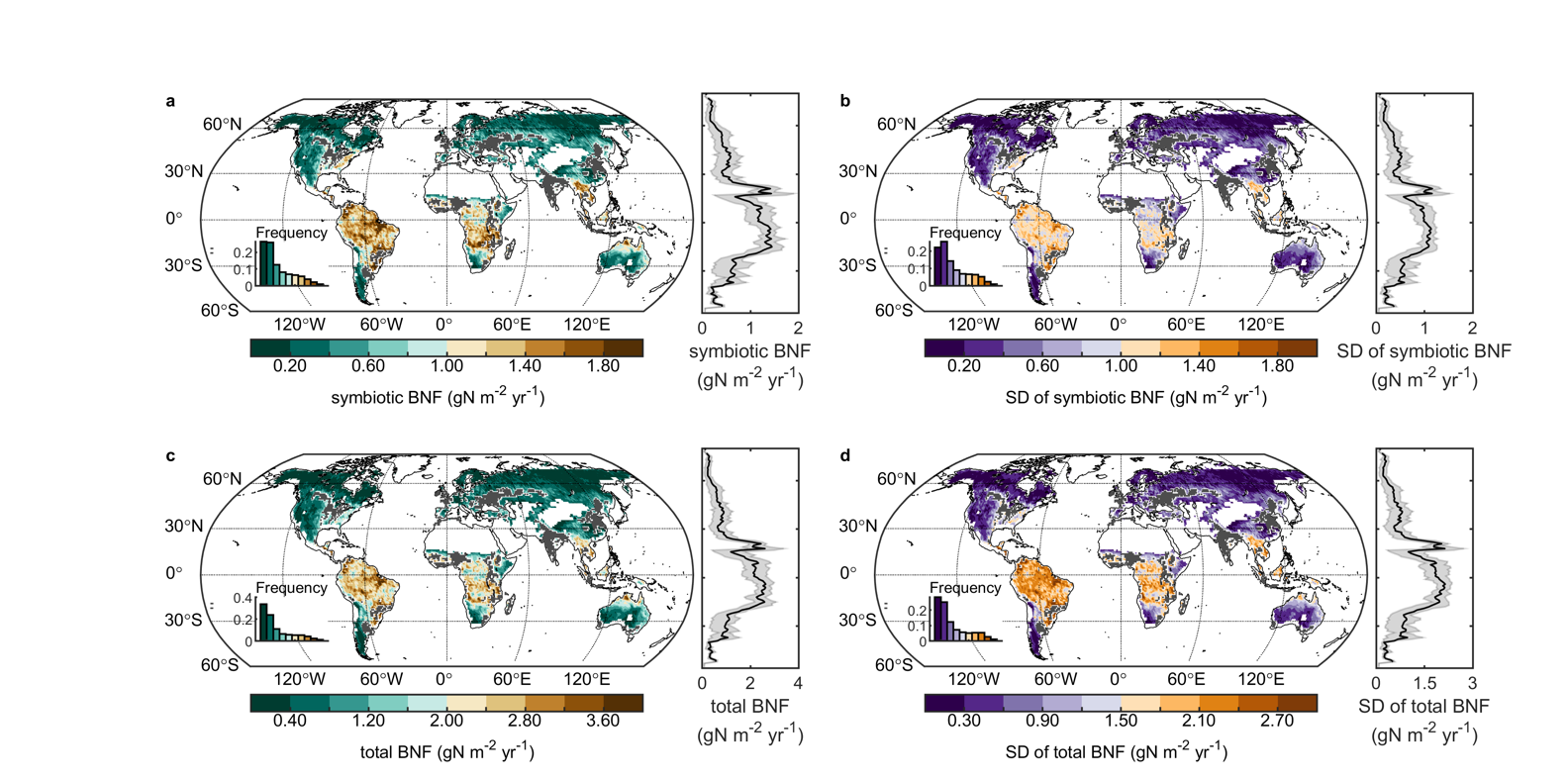
*

**Fig. S18.** Global maps of biological nitrogen fixation (BNF) estimates for natural terrestrial ecosystems derived by Bayesian approach. (**a**) and (**c**), Global maps of symbiotic and total BNF, respectively. (**b**) and (**d**), Global maps of standard deviations (SDs) for symbiotic and total BNF, respectively. The bottom left histograms present the frequency distributions of BNF estimates. In the panels on the right of maps, the black lines indicate the latitudinal means of BNF while the grey areas indicate the 95% confidence intervals. The areas of managed croplands and pasture were excluded in our analysis, and are marked grey.


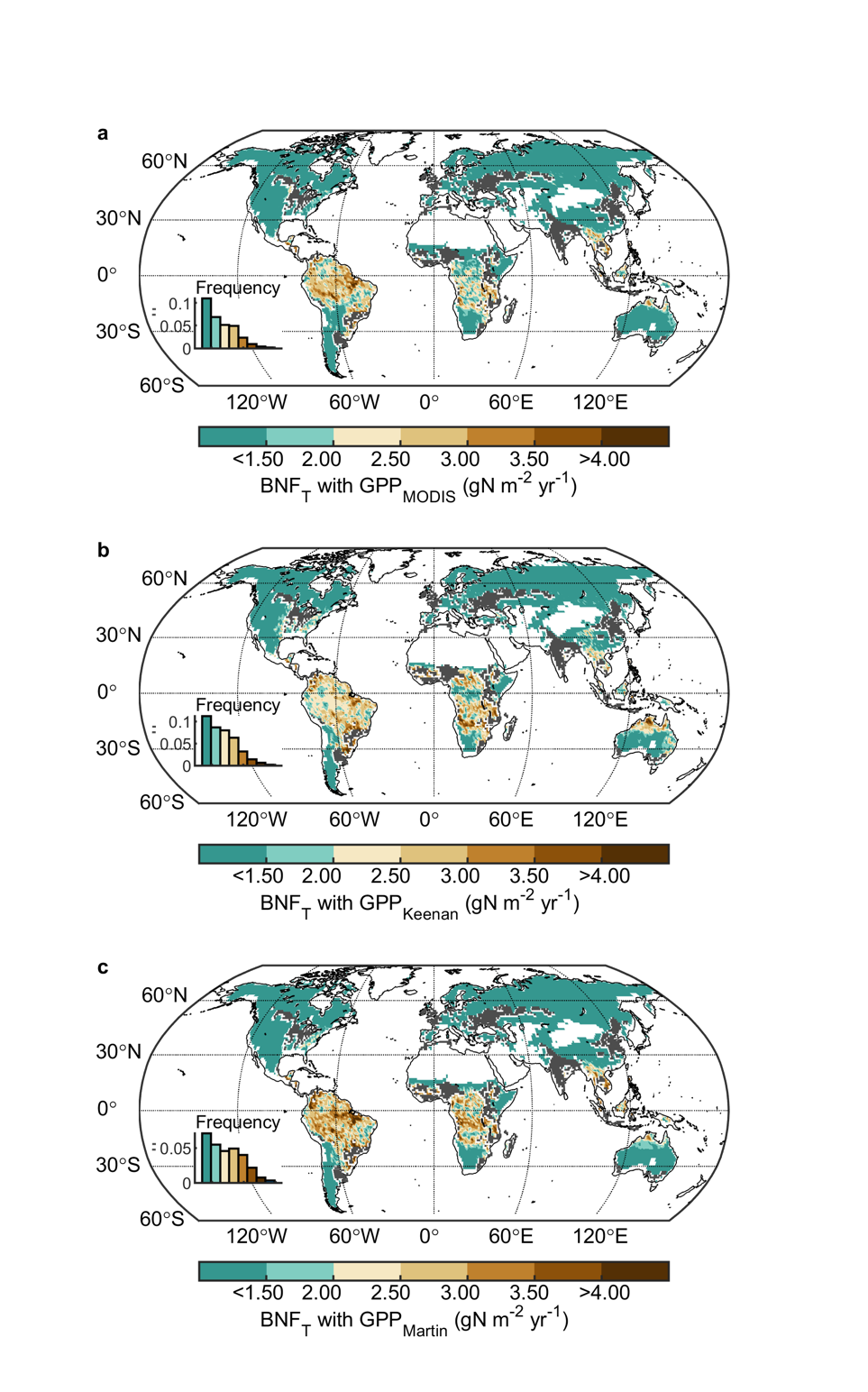


**Fig. S19.** Comparison of global maps of **total** BNF derived from three sources of gross primary production (GPP). (**a**), (**b**), and (**c**) are global maps of total BNF estimated with GPP from MODIS [18], Keenan et al. [19], and Jung et al. [20], respectively. The bottom left histograms present the frequency distributions of BNF estimates. The areas of managed croplands and pastures were excluded in our analysis and are represented by grey regions.


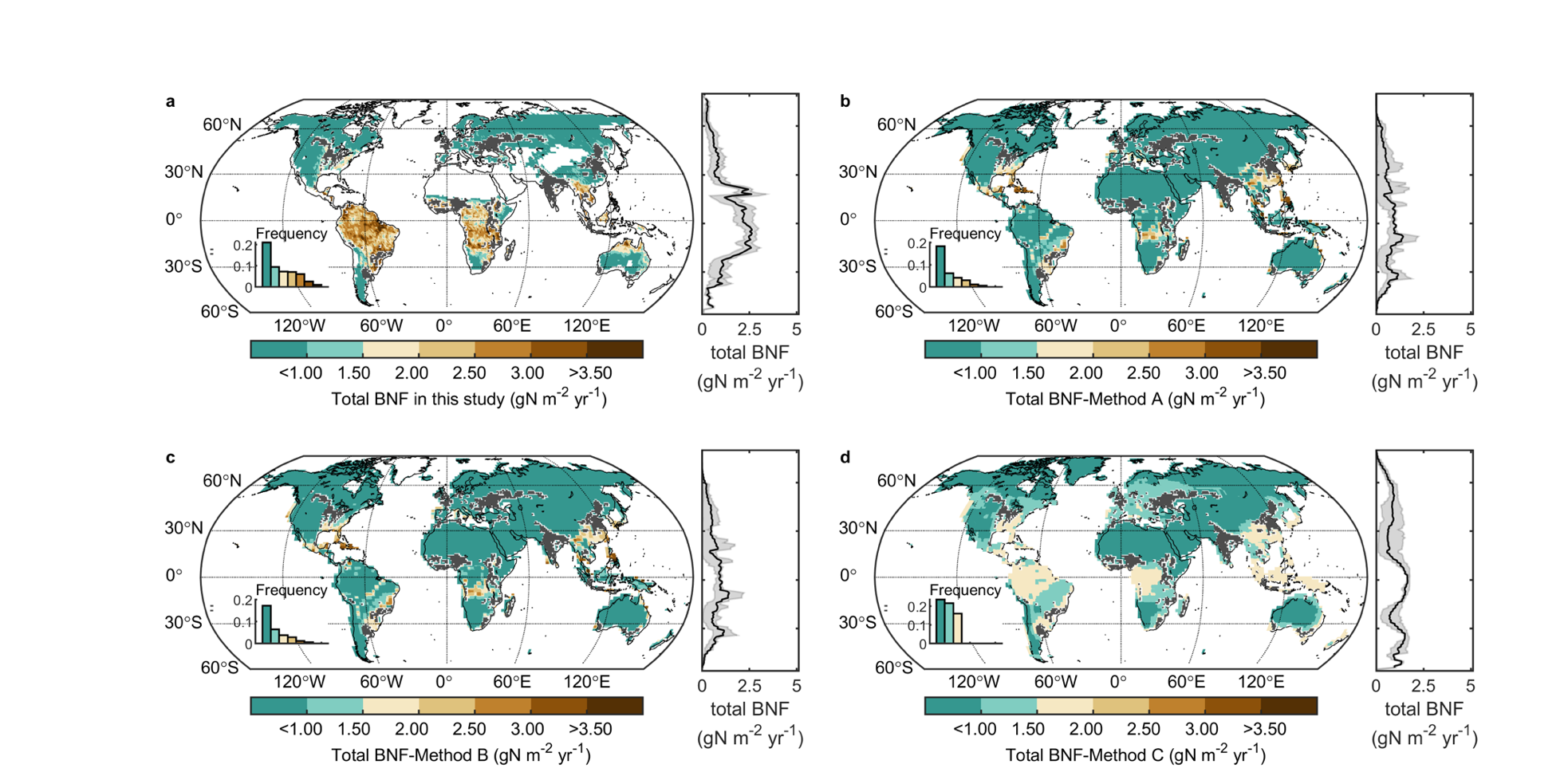


**Fig. S20.** Comparison of global map of total BNF in this study with those simulated by CSCA-CNP model [28]. (**a**) Global pattern of total BNF derived by using the Bayesian approach. (**b**), (**c**), and (**d**) are global patterns of total BNF simulated by CSCA-CNP model with Method A, Method B, and Method C, respectively. The bottom left histograms present the frequency distributions of BNF estimates. The areas of managed croplands and pastures were excluded in our analysis and are represented by grey regions.


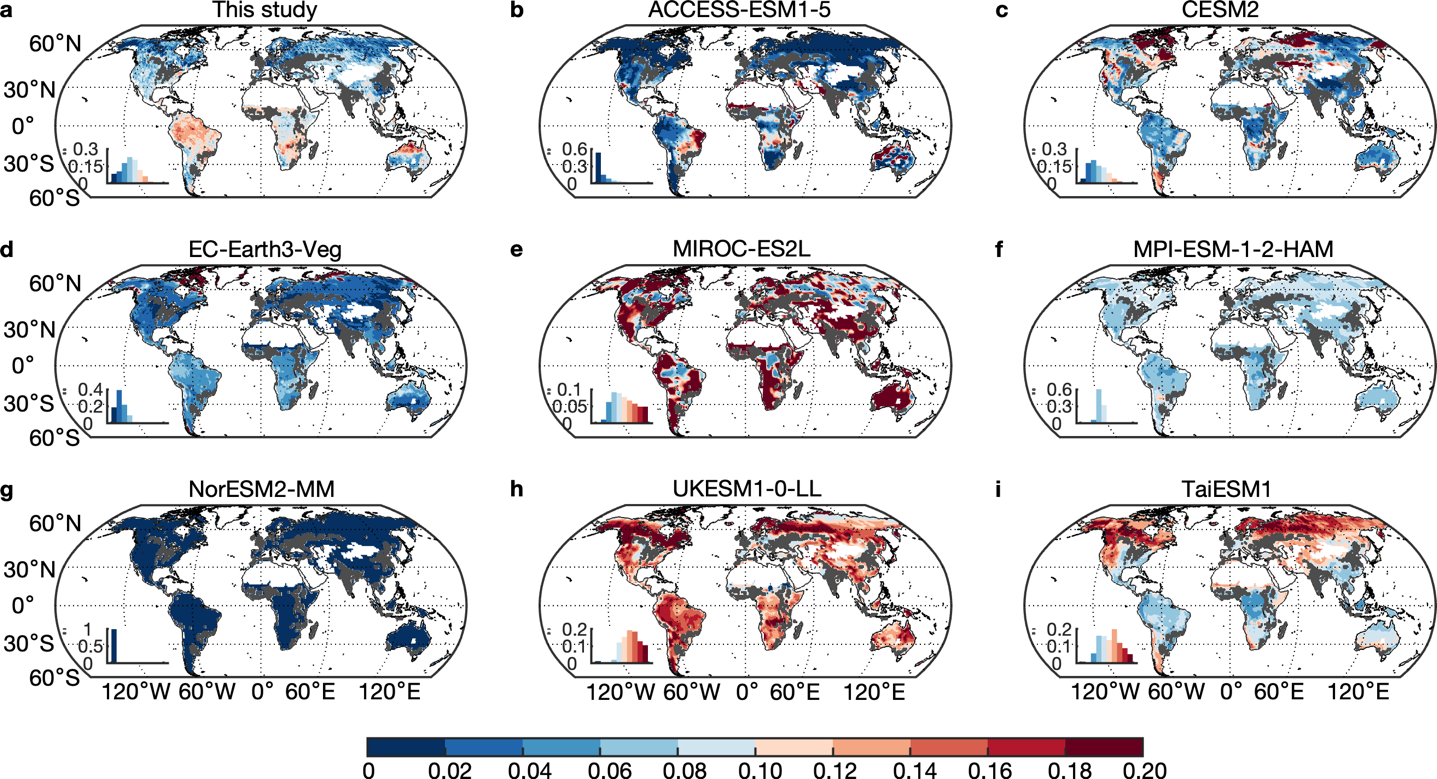


**Fig. S21.** Comparison of global maps of the fraction of symbiotic biological nitrogen fixation (*f*_BNFs_) in vegetation-external nitrogen (N) demands estimated from CMIP6 Earth System Models (ESMs) with the isotope-based estimate in this study. The fractions *f*_BNFs_ of CMIP6 models were estimated by direct outputs of BNF and plant uptake, and the fraction of symbiotic BNF in total BNF (*β*_S_) estimated from Davies-Barnard and Friedlingstein [31]. The areas of managed croplands and pastures were excluded in our analysis and are represented by grey regions.


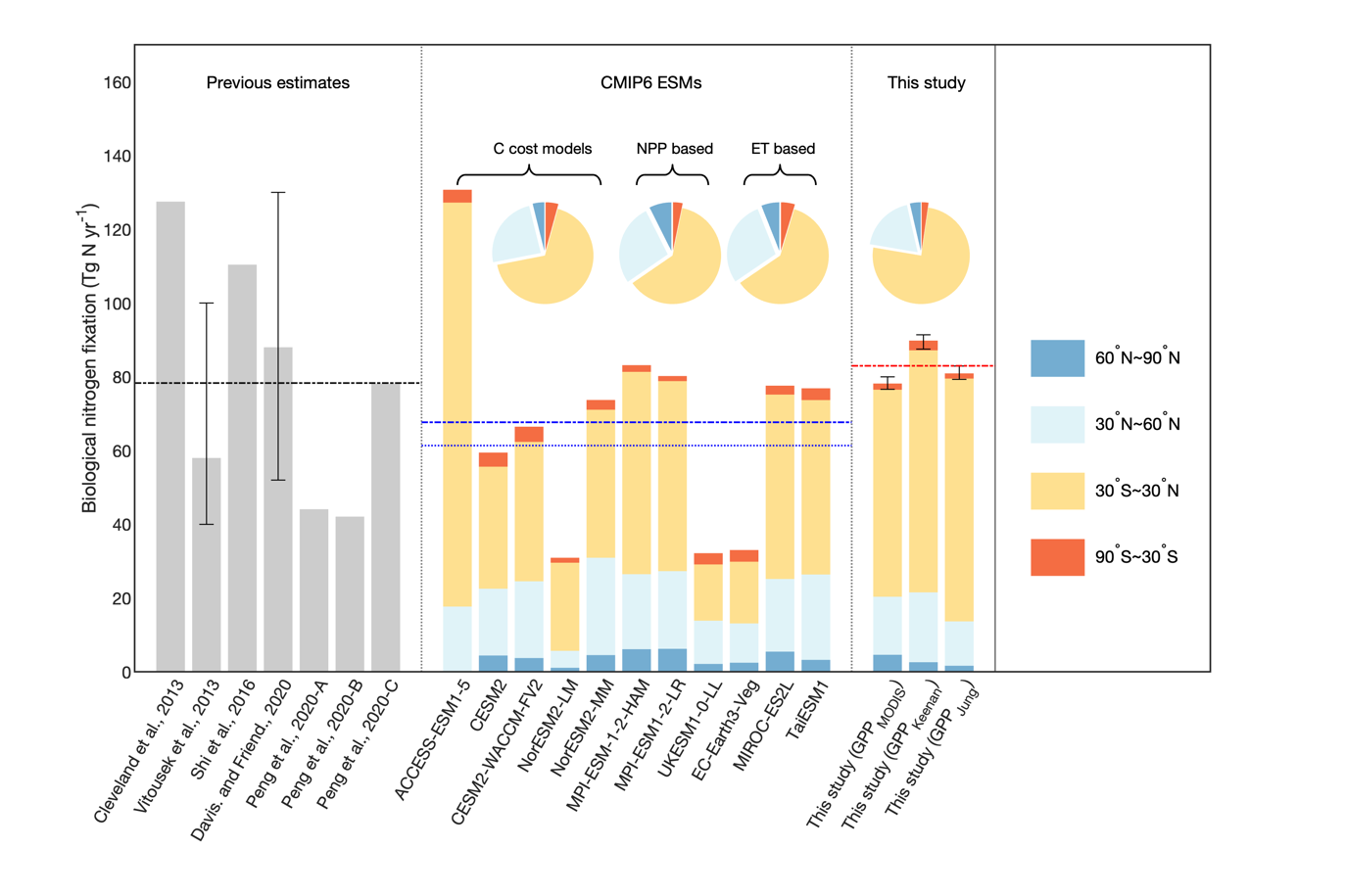


**Fig. S22.** **Underestimation** **of biological nitrogen fixation (BNF) simulated by CMIP6 Earth System Models (ESMs) benchmarked by isotope-based analysis.** The BNF estimates in previous publications are represented by grey bars and classified into several categories based on their approaches: (i) Cleveland *et al.* [4] and Peng *et al.* [28], CASA-CNP model; (ii) Vitousek *et al.* [61], δ^15^N*-*based global N budgets; (iii) Shi *et al.* [5], CLM-FUN2.0 model; and (iv) Davies-Barnard and Friedlingstein [31], global meta-analysis. The colored bars are results from CMIP6 ESMs and our isotope-based study, for which BNF estimates are split into four latitudinal bands (60°–90°N, 30°–60°N, 30°S–30°N, and 90°–30°S) and represented by stacked bars. The CMIP6 model ensemble is subdivided according to BNF mechanistic representation using (i) C cost models, (ii) net primary production (NPP)-based approaches and (iii) evapotranspiration (ET)-based approaches. The means of global BNF fluxes from previous publications, CMIP6 ESMs and this study are represented by black, blue and red dashed lines, respectively. The dotted blue line is the mean BNF of CMIP6 models by excluding the exceptionally high estimate (ACCESS-ESM1-5).


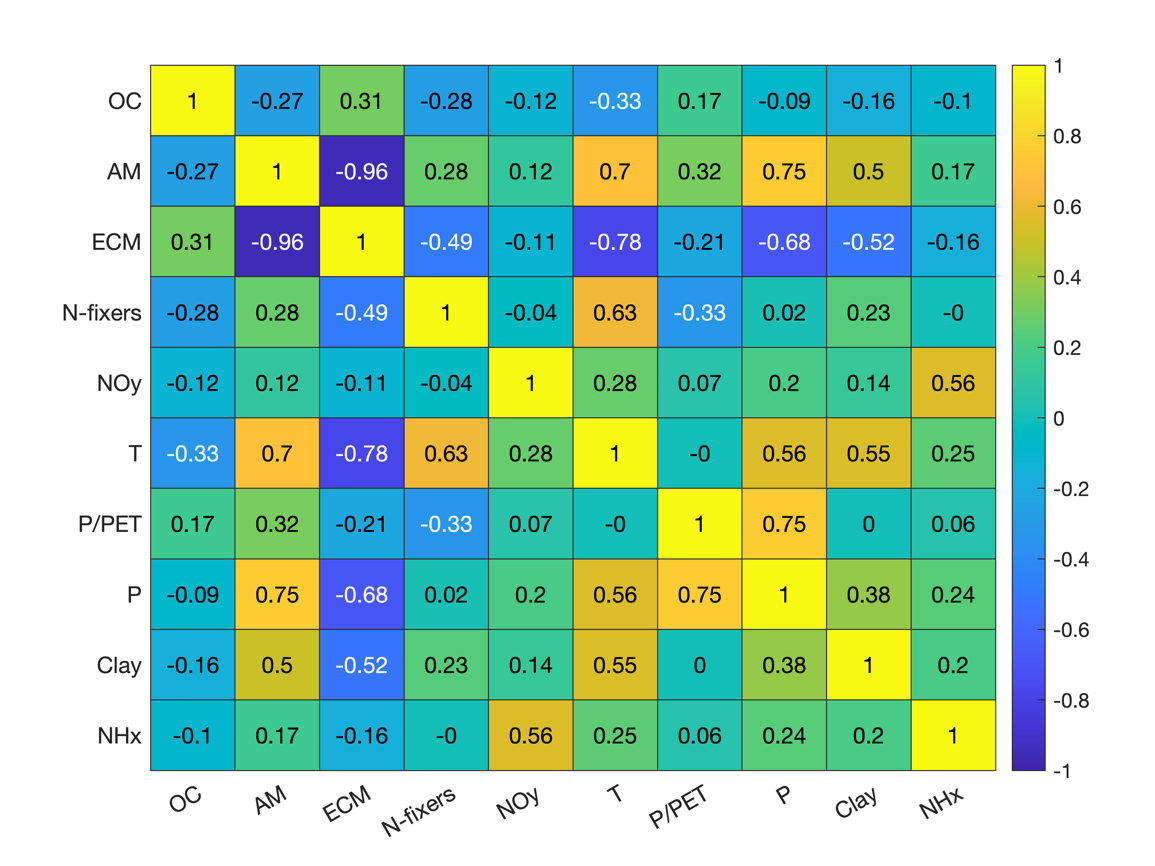


**Fig. S23.** The correlation matrix of the 10 predictors selected from the original 16 predictors with the Recursive Feature Elimination.


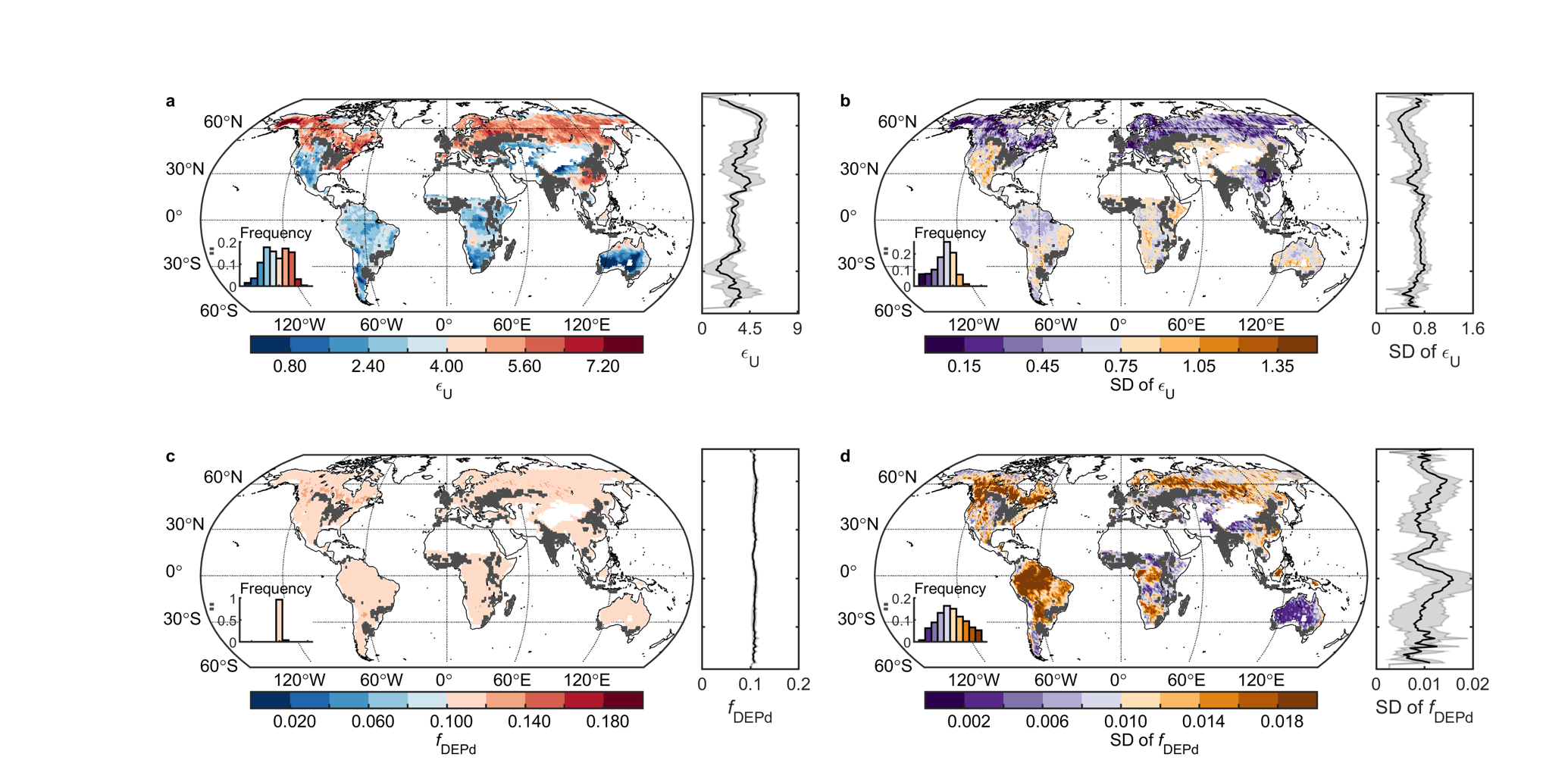


**Fig. S24.** Posterior global map of the isotope fractionation of plant uptake (ε_U_) and the fraction of canopy uptake of N deposition (f_DEPd_) estimated by Bayesian approach. (**a**) and (**b**) are global maps of posterior ε_U_ and its standard deviation (SD), respectively. (**c**) and (**d**) are global maps of posterior f_DEPd_ and its SD, respectively. The areas of managed croplands and pastures were excluded in our analysis and are represented by grey regions.


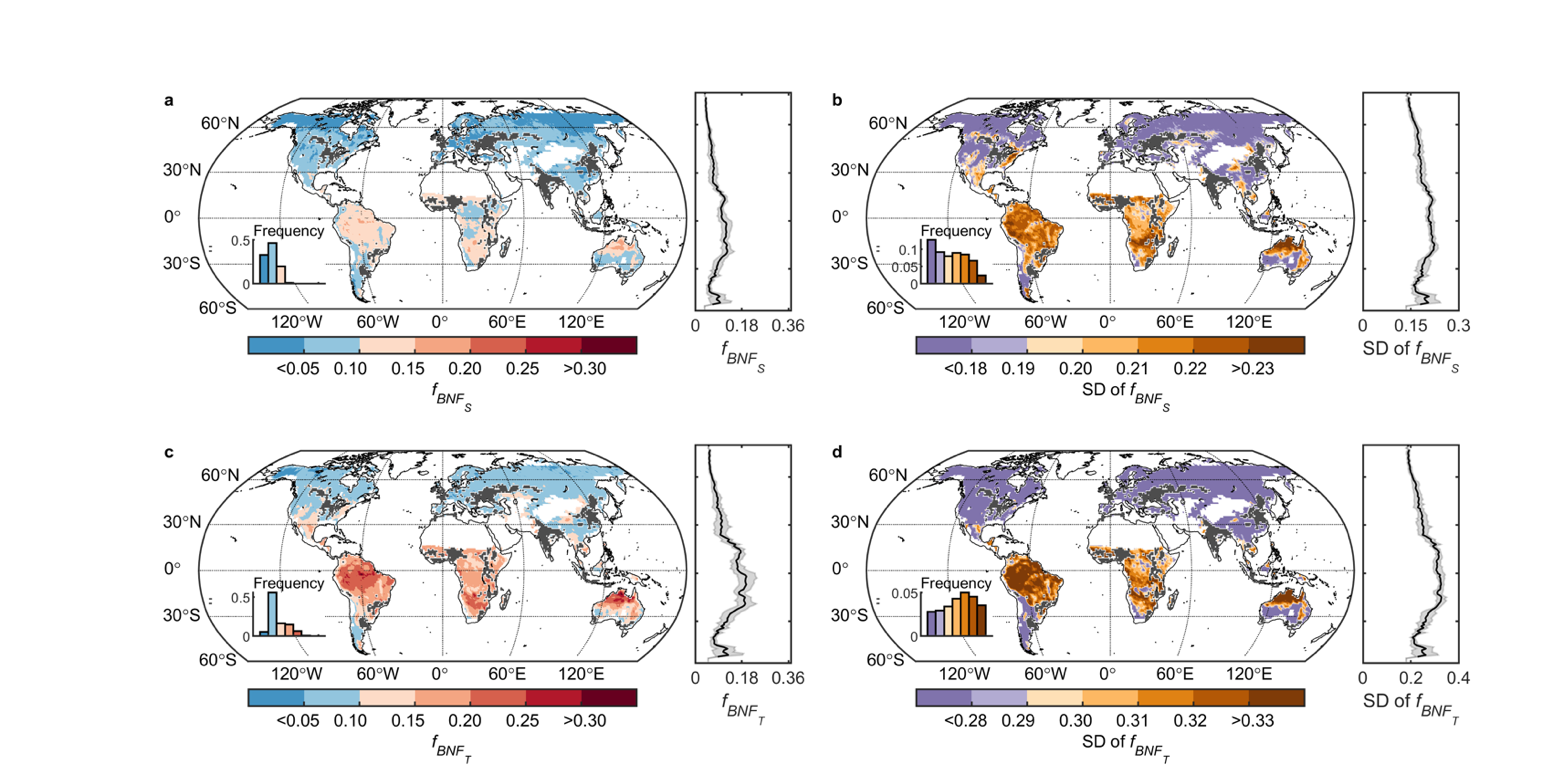


**Fig. S25.** Global maps of the fractions of symbiotic and total BNF (f_BNFs_ and $\text{f}_{\text{BNF}_{\text{T}}}$) over vegetation N demand from external sources in natural terrestrial ecosystems produced by Monte Carlo (MC) approach. (**a**) and (**b**) are the fraction of symbiotic BNF over vegetation-external N demand and its standard deviations (SD), respectively. (**c**) and (**d**) are the fraction of total BNF over vegetation-external N demand and its SD, respectively. The bottom left histograms present the frequency distributions. In the panels on the right of the maps, the black lines indicate the latitudinal means, with their 95% confidence intervals represented by grey areas. The areas of managed croplands and pastures were excluded in our analysis and are represented by grey regions.


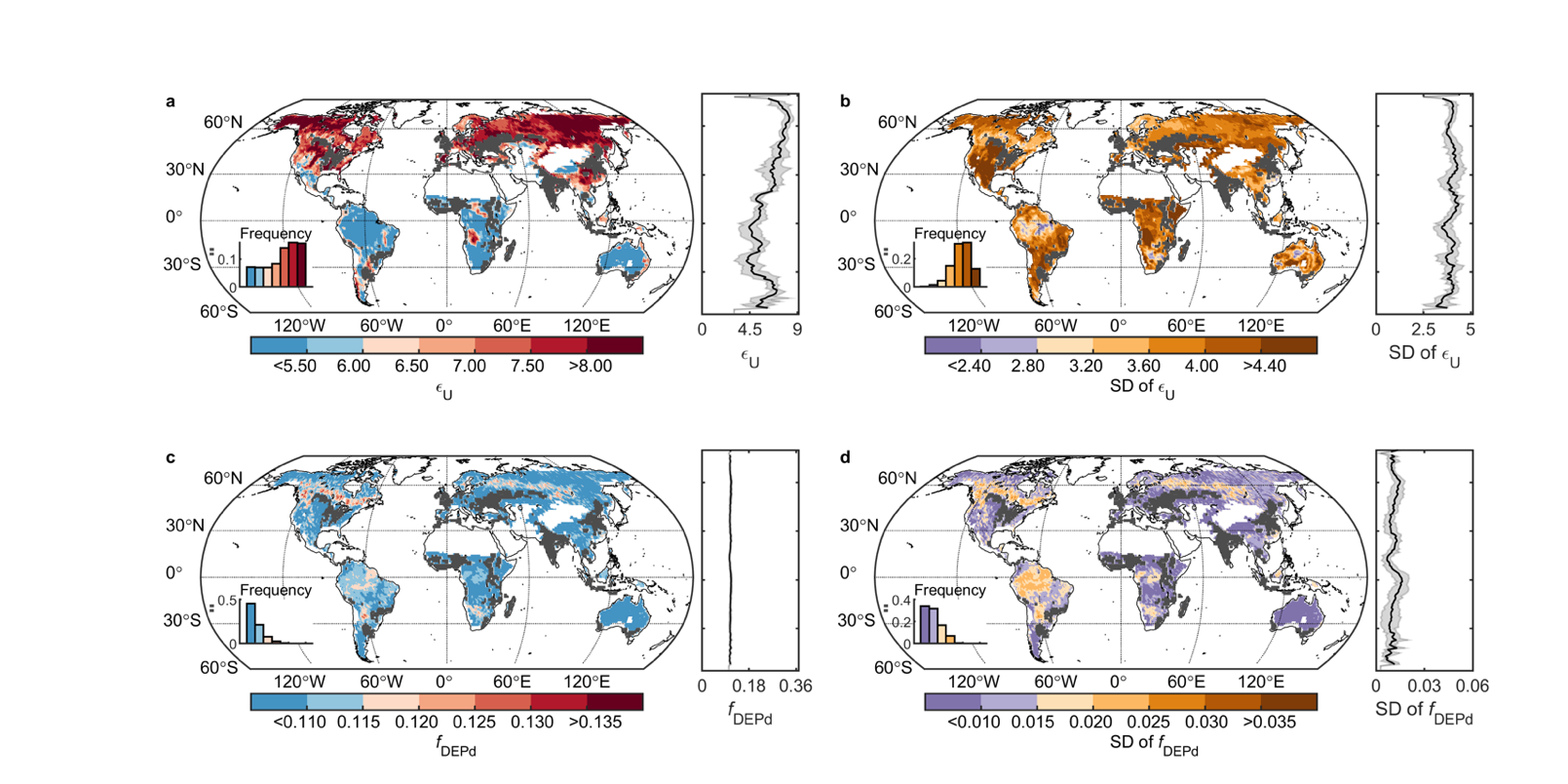


**Fig. S26.** Global maps of the fractionation factor of plant uptake (ε_U_) and the fraction of canopy uptake of N deposition (f_DEPd_) updated by Monte Carlo (MC) approach. (**a**) and (**b**) show the fractionation factor and its standard deviation (SD), respectively. (**c**) and (**d**) are global maps of posterior f_DEPd_ and its SD, respectively. The bottom left histograms present the frequency distributions. In the panels on the right of the maps, the black lines indicate the latitudinal means, with their 95% confidence intervals represented by grey areas. The areas of managed croplands and pastures were excluded in our analysis and are represented by grey regions.


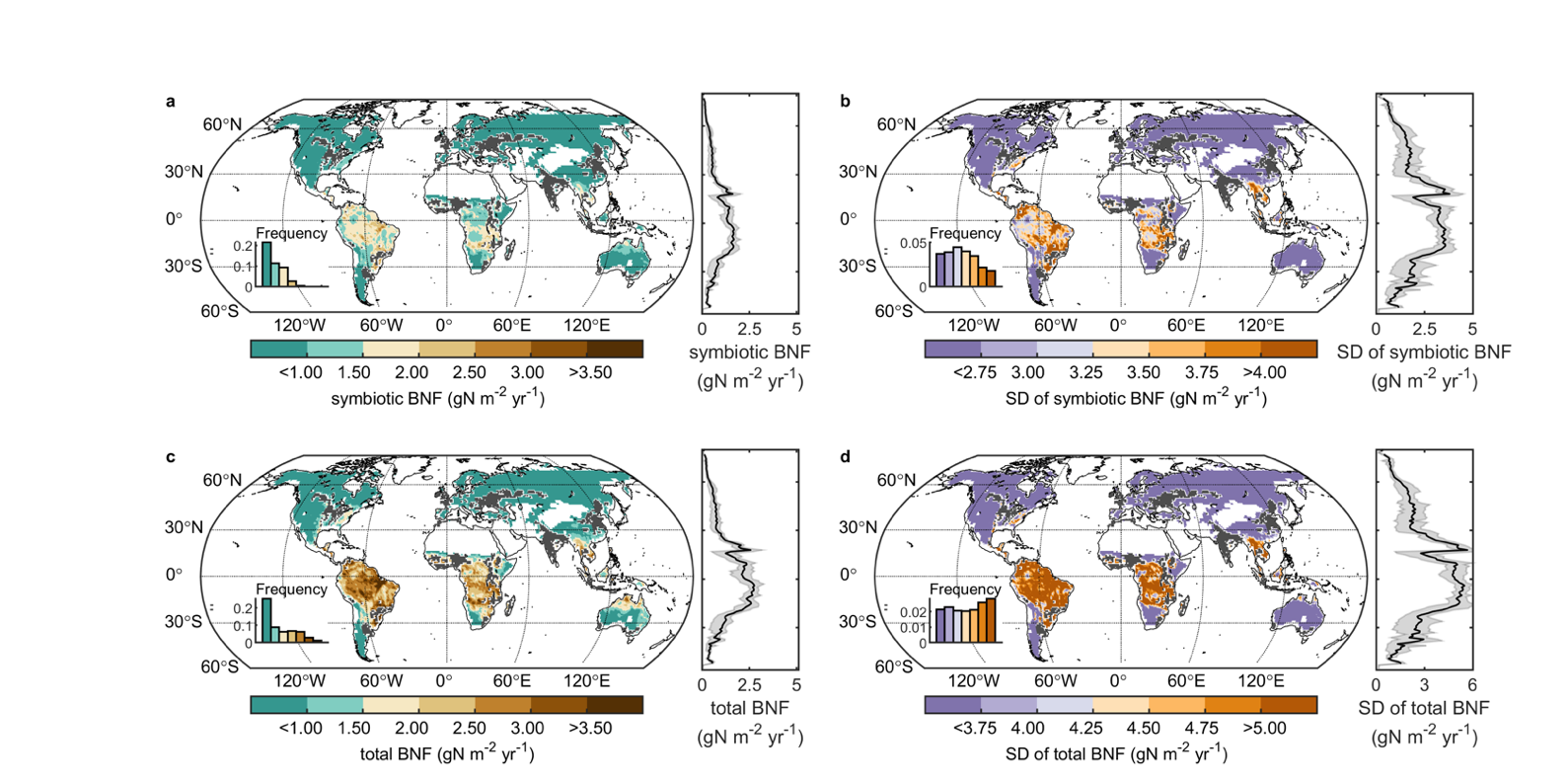


**Fig. S27.** Global maps of symbiotic and total biological nitrogen fixation (BNF) in natural terrestrial ecosystems derived by Monte Carlo (MC) approach. (**a**) and (**b**) are symbiotic BNF and its standard deviation (SD), respectively. (**c**) and (**d**) are total BNF and its SD, respectively. The bottom left histograms present the frequency distributions. In the panels on the right of the maps, the black lines indicate the latitudinal means, with their 95% confidence intervals represented by grey areas. The areas of managed croplands and pastures were excluded in our analysis and are represented by grey regions.


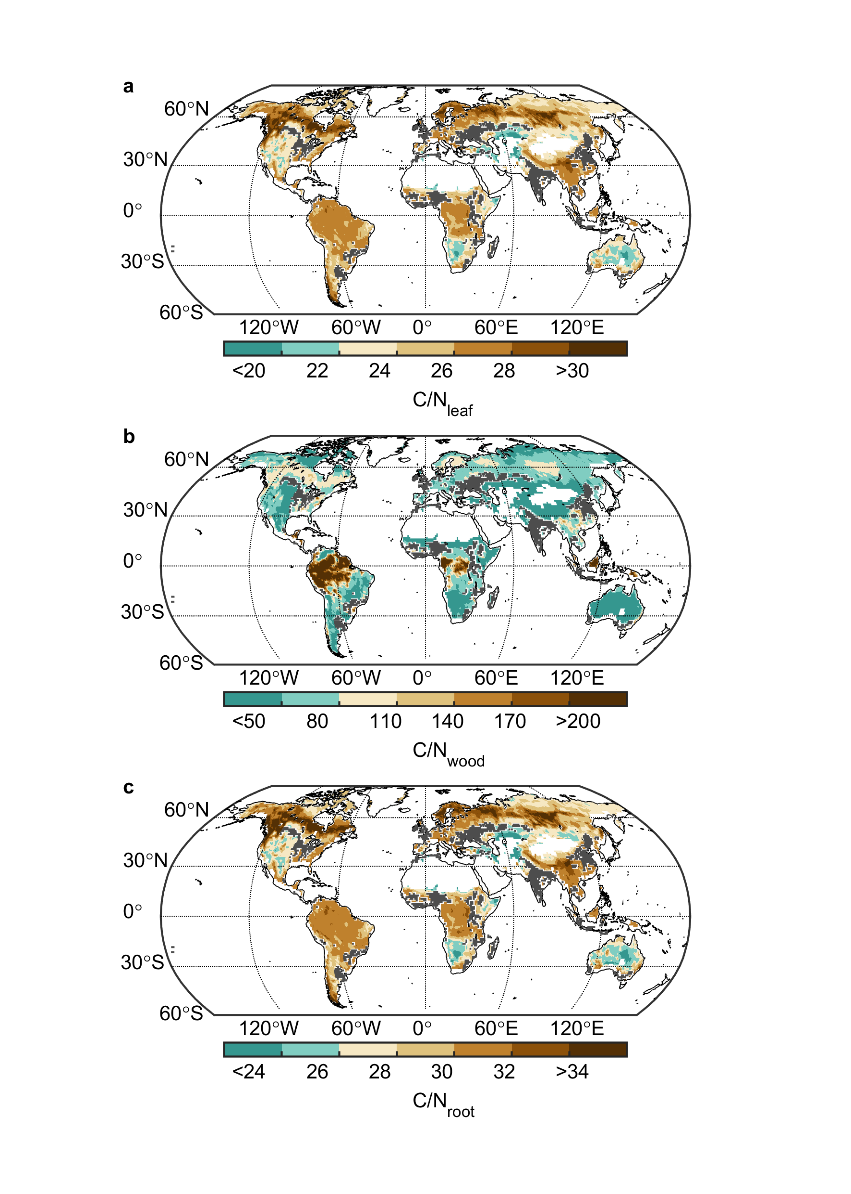


**Fig. S28.** Global maps of C:N ratios used for the estimation of vegetation N demand from external sources. (**a**), (**b**), and (**c**) are global maps of C:N ratios for leaf, wood, and root, respectively. The areas of managed croplands and pastures were excluded in our analysis and are represented by grey regions.

**Table S1.** Pairwise δ*_S_* and δ*_P_* observations collected from 13 natural forests in China and several sites collected from literature. This table was compiled from Gurmesa *et al.* [29].

| Number | Site | Longitude | Latitude | MAT | MAP (mm) | Foliage δ^15^N | Mean of mineral soil δ^15^N |
| --- | --- | --- | --- | --- | --- | --- | --- |
| 1 | Changbai mountain | 128° 08'E | 42° 38'N | 4.00 | 745.00 | -4.13 | 0.97 |
| 2 | Maoershan | 127° 58'E | 45° 33'N | 3.00 | 700.00 | -0.40 | 6.65 |
| 3 | Qing yuan forest-L | 124° 91'E | 41° 85'N | 5.00 | 775.00 | -1.80 | 6.00 |
| 4 | Qing yuan forest-M | 124° 90'E | 41° 86'N | 5.00 | 775.00 | -0.50 | 5.80 |
| 5 | Wuyishan | 117° 78'E | 27° 87'N | 14.00 | 2500.00 | -1.74 | 5.28 |
| 6 | Tieshanping | 106° 68'E | 29° 63'N | 18.00 | 1105.00 | -6.56 | 1.33 |
| 7 | Jianfengling-P | 108° 89'E | 18° 73'N | 20.00 | 2449.00 | -1.30 | 3.10 |
| 8 | Jianfengling-S | 108° 85'E | 18° 75'N | 20.00 | 2449.00 | -1.42 | 4.86 |
| 9 | Xishuangbanna | 101° 25'E | 21° 93'N | 21.00 | 1593.00 | -0.14 | 5.07 |
| 10 | Wülfersreuth | 12° E | 50° N | 5.9 | 1072 | -5.31 | 4.20 |
| 11 | Solling | 9° 34' E | 51° 31'N | 6.4 | 1090 | -2.50 | 5.20 |
| 12 | Harvard Forest-H | 72° 10'W | 42° 30'N | 7.0 | 1120 | -2.89 | 4.74 |
| 13 | Harvard Forest-R | 72° 10'W | 42° 30'N | 7.0 | 1120 | -1.83 | 5.11 |
| 14 | Maoershan+N | 127° 58'E | 45° 33'N | 2.8 | 700 | 0.80 | 6.75 |
| 15 | Tieshanping+N | 106° 68'E | 29° 63'N | 18.2 | 1105 | -5.27 | 1.57 |
| 16 | Tieshanping+N | 106° 68'E | 29° 63'N | 18.2 | 1105 | -3.36 | 2.56 |
| 17 | Speuld | 5° 39'W | 52° 13'N | 9.30 | 750.0 | -2.00 | 3.50 |
| 18 | Beddington, Maine, USA | 68° 6' 25''W | 44° 51'N 55'' | 4.90 | 1400.00 | -1.83 | 5.90 |
| 19 | Lower Michigan, USA | 84° 51'W | 44° 33'N | 5.60 | 870.00 | -1.99 | 3.94 |
| 20 | Dinghushan | 112° 10E | 23° 10'N | 21.00 | 1927 | -3.90 | 2.20 |
| 21 | Dinghushan | 112° 10E | 23° 10'N | 21.00 | 1927 | -4.90 | 2.60 |

**Table S2.** Climosequence data of δ*_S_* and δ*_P_* observations compiled by Amundson *et al.* [13]

| Number | Elevation, m | MAT, C | MAP, mm | Soil δ^15^N to 10 cm, ‰ | Soil δ^15^N to 50 cm, ‰ | δ^15^N Plants | Δplant−soil to 10 cm, ‰ | Δplant−soil to 50 cm, ‰ |
| --- | --- | --- | --- | --- | --- | --- | --- | --- |
| 1 | 1400 | 14 | 326 | 5.9 | 6.8 | 2.9 | −3 | −3.9 |
| 2 | 1750 | 12 | 436 | 3.7 | 4.9 | -2.2 | −5.9 | −7.1 |
| 3 | 470 | 18 | 330 | 1.5 | 2.8 | -1.9 | −3.4 | −4.7 |
| 4 | 1240 | 12 | 910 | 0.8 | 2.6 | -4.5 | −5.3 | −7.1 |
| 5 | 2890 | 3 | 1270 | 2.5 | 4.7 | -2.4 | −4.9 | −7.1 |
| 6 | 1829 | 19 | 1970 | 2.2 | 3.6 | -1.2 | −3.4 | −4.8 |
| 7 | 2454 | 14 | 1670 | 2.3 | 4.5 | -1.1 | −3.4 | −5.6 |
| 8 | 2545 | 14 | 1570 | 2.1 | 4.9 | -1.5 | −3.6 | −6.4 |
| 9 | 2990 | 12 | 1320 | 3.6 | 5.4 | -2.1 | −5.7 | −7.5 |
| 10 | 3505 | 9 | 1040 | 4.8 | 6 | -3.1 | −7.9 | −9.1 |
| 11 | 1370 | 16 | 2209 | 5.79 | 5.17 | 2 | −3.79 | −3.17 |
| 12 | 1370 | 16 | 2435 | 6.12 | 4.47 | 1 | −5.12 | −3.47 |
| 13 | 1370 | 16 | 2759 | 4.87 | 5.61 | -1 | −5.87 | −6.61 |
| 14 | 1320 | 16 | 3338 | 7.35 | 6.27 | 0.8 | −6.55 | −5.47 |
| 15 | 1300 | 16 | 4030 | 3.2 | 3.46 | -2 | −5.2 | −5.46 |
| 16 | 1270 | 16 | 5066 | 2.95 | 0.65 | -5 | −7.95 | −5.65 |
| 17 | 1100 | 7 | 1410 | 4.14 | 5.1 | -1.9 | −6.04 | −7 |
| 18 | 1440 | 5 | 1683 | 4.27 | 4.8 | -1.8 | −6.07 | −6.6 |
| 19 | 1600 | 4 | 1811 | 2.66 | 3.8 | -5 | −7.66 | −8.8 |
| 20 | 1800 | 3 | 1972 | 1.55 | 3 | -2.8 | −4.35 | −5.8 |

**Table S3.** Predictors of Random Forest models for global maps of foliar δ^15^N, soil δ^15^N, and their difference.

| Variables | Long-name | Datasets | Period | Resolution (°) |
| --- | --- | --- | --- | --- |
| BD | Bulk density | BNU [33] | - | 1/120 |
| pH | Soil pH | BNU [33] | - | 1/120 |
| Clay | Fraction of clay in 0-30cm | BNU [33] | - | 1/120 |
| Sand | Fraction of sand in 0-30cm | BNU [33] | - | 1/120 |
| Silt | Fraction of silt in 0-30cm | BNU [33] | - | 1/120 |
| OC | Organic C in 0-30cm | BNU [33] | - | 1/120 |
| C/N | Soil C:N ratio in 0-30cm | BNU [33] | - | 1/120 |
| AM | Arbuscular mycorrhizal fungi | Steidinger *et al.* [34] | - | 1 |
| ECM | Ectomycorrhizal fungi | Steidinger *et al.* [34] | - | 1 |
| Nfix | Nitrogen fixing bacteria | Steidinger *et al.* [34] | - | 1 |
| GPP | Gross primary production | MODIS [18] | 2000-2019 | 1/10 |
|  |  | Keenan *et al.* [19] | 1982-2015 | 0.5 |
|  |  | Jung *et al.* [20] | 1988-2017 | 0.5 |
| NHx | Monthly NHx deposition | Tian *et al.* [35] | 1981-2016 | 0.5 |
| NOy | Monthly NOy deposition | Tian *et al.* [35] | 1981-2016 | 0.5 |
| P | Mean annual precipitation | CRU TS v4.03 | 1981-2018 | 0.5 |
| P/ET | Mean annual aridity index | CRU TS v4.03 | 1981-2018 | 0.5 |
| T | Mean annual temperature | CRU TS v4.03 | 1981-2018 | 0.5 |

**Table S4.** Earth System Models (ESMs) in CMIP6 used in this study. S & F: symbiotic and free-living BNF; ND: No difference between symbiotic and free-living BNF.

| Source ID | Land surface model | Experiment | Variant | BNF types | Where BNF goes |
| --- | --- | --- | --- | --- | --- |
| ACCESS-ESM1-5 | CABLE2.4 | historical | r1i1p1f1 | S & F | Mineral |
| CESM2 | CLM5 | historical | r10i1p1f1 | S & F | Plant and Mineral |
| CESM2-WACCM-FV2 | CLM5 | historical | r1i1p1f1 | S & F | Plant and Mineral |
| MIROC-ES2L | MATSIRO6.0+VISIT-e ver.1.0 | historical | r1i1p1f2 | S & F | Plant and Mineral |
| UKESM1-0-LL | JULES-ES-1.0 | historical | r1i1p1f2 | ND | Plant |
| NorESM2-LM | CLM5 | historical | r1i1p1f1 | S & F | Plant and Mineral |
| NorESM2-MM | CLM5 | historical | r1i1p1f1 | S & F | Plant and Mineral |
| EC-Earth3-Veg | LPJ-GUESS v4 | historical | r5i1p1f1 | ND | Mineral |
| MPI-ESM-1-2-HAM | JSBACH 3.20 | historical | r1i1p1f1 | ND | Mineral |
| MPI-ESM1-2-LR | JSBACH3.20 | historical | r1i1p1f1 | ND | Mineral |
| TaiESM1 | CLM4.5 | historical | r1i1p1f1 | ND | Mineral |

**Table S5.** Summary of mathematical representations of symbiotic and free-living biological nitrogen fixation (BNF) in CMIP6 Earth System Models (ESMs).

| C-N module | ESMs | Symbiotic BNF | Free-living BNF | References |
| --- | --- | --- | --- | --- |
| CASA-CNP | ACCESS-ESM1-5 | *F_n,fix_ = v_fix_f_fix_C_fix_* $\text{f}_{\text{fix}}\text{=1-}\text{f}_{\text{up}}\text{=1-}\text{min(}\text{1.0,}\frac{\text{λ}_{\text{fix}}}{\text{λ}_{\text{up}}^{\text{*}}}\text{)}$  $\text{λ}_{\text{up}}\text{=}\frac{\frac{\text{∂}\text{F}_{\text{C,fix}}}{\text{∂}\text{C}_{\text{fix,l}}}}{\frac{\text{∂}\text{F}_{\text{N,fix}}}{\text{∂}\left( \text{f}_{\text{up}}\text{C}_{\text{fix,r}} \right)}}\text{=}\text{f}_{\text{up}}\frac{\frac{\text{∂}\text{F}_{\text{C,fix}}}{\text{∂}\text{C}_{\text{fix,l}}}}{\frac{\text{∂}\text{F}_{\text{N,fix}}}{\text{∂}\text{C}_{\text{fix,r}}}}\text{=}\text{f}_{\text{up}}\text{λ}_{\text{up}}^{\text{*}}$  where *F_n,fix_* is the symbiotic nitrogen fixation rate, *v_fix_* is the rate of N fixed per unit of nodulated root carbon (g N (g C)^−1^ year^−1^), and *C_fix_* is the C used for nitrogen fixation. *f_fix_* and *f_up_* are the fractions of nodulated and non-nodulated root area, respectively. $\text{λ}_{\text{up}}$ and $\text{λ}_{\text{fix}}$ are the cost of nitrogen uptake and fixation, respectively. | linear functions of ET or NPP, following Cleveland *et al.*[4] | Wang *et al.* [24] |
| CLM5/CLM4.5 | CESM2 family;  NorESM2 family;  AWI-ESM-1-1-LR;  TaiESM1 family | In CLM5 (for CESM2 and NorESM2), Fixation of Nitrogen and Uptake (FUN) Model;  In CLM4.5 (for AWI-ESM-1-1-LR and TaiESM1),  $\text{N}\text{F}_{\text{nfix,sminn}}\text{=}\frac{\text{1.8}\left( \text{1-}\exp\left( \text{-0.003C}\text{F}_{\text{an}\text{n}_{\text{npp}}} \right) \right)}{\text{86400∙365}}$  where $\text{N}\text{F}_{\text{nfix,sminn}}$ is the rate of total BNF rate (g N m^-2^ day^-1^), and $\text{C}\text{F}_{\text{an}\text{n}_{\text{npp}}}$ is the net primary production (gC m^-2^ yr^-1^) | $\text{N}\text{F}_{\text{nfix,sminn}}\text{=}\frac{\text{0.006}\left( \text{0.0117+C}\text{F}_{\text{ann\_ET}}\text{ } \right)}{\text{86400∙365}}$  where $\text{N}\text{F}_{\text{nfix,sminn}}$ is the rate of free-living BNF rate (g N m^-2^ day^-1^), and $\text{C}\text{F}_{\text{ann\_ET}}$ is annual evapotranspiration (mm yr^-1^) | Technical note of CLM5.0/CLM4.5 |
| LPJ-GUESS v4 | EC-Earth3 family | *BNF*=0.0102*AET*+0.524  where BNF is the rate of total BNF (kg N ha yr^-1^), and AET (mm yr^-1^) is the 5-year average actual evapotranspiration. | Incorporated into total BNF | Smith *et al.* [36] |
| JSBACH v3.20 | MPI-ESM family | $\text{BNF=}\text{f}_{\text{bnf}}\text{×}\left( \text{1-}\text{e}^{\text{f}_{\text{emp}}\text{w}_{\text{C}}\bar{\text{NPP}}} \right)\frac{\text{w}_{\text{N}}}{\text{w}_{\text{C}}}$  where f_emp_ = -0.003 day g^-1^ C is an empirical relationship from (Cleveland *et al.*, 1999), *f*_bnf_ = 0.7 g N m-2 day^-1^ is the calibrated parameter to achieve a global sum of BNF of 100 Mt yr^-1^ for a simulated NPP of 65 Gt yr^-1^; w_N_ and w_C_ are standard atomic weights of nitrogen and carbon, respectively | Incorporated into total BNF | Goll *et al.* [37] |
| VISIT-e | MIROC-ES2L | Using formula and parameterization in Cleveland *et al.* [4] | Incorporated into total BNF | Inatomi *et al.* [38] |
| JULES-ES-1.0 | UKESM1-0-LL | *BNF_i_* = ζ*NPP_pot,i_*  where *BNF_i_* is the biological nitrogen fixation of plant function type (PFT) *i*; and *NPP_pot,i_* is the net primary production of PFT *i* before considering the N limitation; ζ is the rate of fixation, set as 0.0016 kg N kg C^-1^, such that the present day NPP of 60 Pg C yr^-1^ results in 100 Tg N yr^-1^. | Incorporated into total BNF | Wiltshire *et al.* [39] |

**Table S6.** Comparison of biome-level symbiotic and total biological nitrogen fixation (BNF) estimated from three different products of gross primary production (GPP) (Bayesian approach). The three GPP sources are from MODIS [18], Keenan et al. [19], and Jung et al. [20]. In this table, the fraction of canopy uptake of N deposition to vegetation N demand (f_DEPd,tot_) is set as 10%.

| PFT | Area (10^6^ km^2^) | GPP from MODIS | | | |  | GPP from Jung | | | GPP from Keenan | | | |
| --- | --- | --- | --- | --- | --- | --- | --- | --- | --- | --- | --- | --- | --- |
|  |  | Symbiotic BNF | | Total BNF | | Symbiotic BNF | | Total BNF | | Symbiotic BNF | | Total BNF | |
|  |  | Mean | Median  (Min.–Max. of 95% CI) | Mean | Median  (Min.–Max. of 95% CI) | Mean | Median  (Min.–Max. of 95% CI) | Mean | Median  (Min.–Max. of 95% CI) | Mean | Median  (Min.–Max. of 95% CI) | Mean | Median  (Min.–Max. of 95% CI) |
| ENF | 10.8 | 4.2 | 4.1  (4.0–4.1) | 6.3 | 6.3  (6.2–6.4) | 3.6 | 3.6  (3.5–3.7) | 5.6 | 5.6  (5.5–5.7) | 4.7 | 4.7  (4.7–4.8) | 7.2 | 7.2  (7.1–7.3) |
| EBF | 15.9 | 14.5 | 14.6  (14.1–14.7) | 26.1 | 26.0  (25.7–27.0) | 15.8 | 15.5  (15.5–16.3) | 28.7 | 28.4  (28.0–29.7) | 14.0 | 14.1  (13.6–14.1) | 25.2 | 25.7  (24.4–25.8) |
| DNF | 3.8 | 1.2 | 1.1  (1.1–1.2) | 1.6 | 1.6  (1.6–1.7) | 0.5 | 0.5  (0.5–0.5) | 0.7 | 0.8  (0.7–0.8) | 0.8 | 0.9  (0.9–0.9) | 1.2 | 1.2  (1.2–1.2) |
| DBF | 9.8 | 10.8 | 10.8  (10.6–11.0) | 18.1 | 18.2  (17.8–18.5) | 11.5 | 11.7  (11.3–11.8) | 19.8 | 19.9  (19.6–20.2) | 13.2 | 13.2  (13.1–13.6) | 22.4 | 22.3  (21.8–22.7) |
| Grass | 33.6 | 16.0 | 15.8  (15.7–16.4) | 26.0 | 26.0  (25.4–26.5) | 15.6 | 15.7  (15.2–16.0) | 26.0 | 26.2  (25.5–26.5) | 20.5 | 20.5  (20.3–20.9) | 33.8 | 33.3  (33.1–34.5) |
| Total | 81.0 | 46.5 | 46.5  (45.6–47.4) | 78.2 | 78.0  (76.6–80.0) | 47.0 | 46.9  (46.0–48.4) | 80.9 | 80.9  (79.3–82.9) | 53.4 | 53.4  (52.6–54.3) | 89.8 | 89.6  (87.5–91.4) |

**Table S7.** Sensitivity of f_BNFs_, f_BNFT_, symbiotic and total BNF fluxes to f_DEPd,tot_ (equivalent to f_DEPd_).

| *f*_DEPd,tot_ | 0 | 0.05 (or 5%) | 0.1 (or 10%) | 0.15 (or 15%) |
| --- | --- | --- | --- | --- |
| *f*_BNFs_ | 0.071 | 0.067 | 0.063 | 0.060 |
| *f*_BNFT_ | 0.113 | 0.107 | 0.101 | 0.096 |
| BNF_S_ (Tg N yr^-1^) | 55.2 | 51.9 | 49.0 | 46.1 |
| BNF_T_ (Tg N yr^-1^) | 93.6 | 87.9 | 83.0 | 78.1 |

**Table S8.** Sensitivity of biome-level symbiotic and total biological nitrogen fixation (BNF) to the fraction of canopy uptake of N deposition to vegetation N demand (f_DEPd,tot_). This table provides the means and ranges of BNF estimates derived from three GPP products from MODIS [18], Keenan et al. [19], and Jung et al. [20].

| PFT | Area (10^6^ km^2^) | *f*_DEPd,tot_=5% | | | |  | *f*_DEPd,tot_=10% | | | *f*_DEPd,tot_=15% | | | |
| --- | --- | --- | --- | --- | --- | --- | --- | --- | --- | --- | --- | --- | --- |
|  |  | Symbiotic BNF | | Total BNF | | Symbiotic BNF | | Total BNF | | Symbiotic BNF | | Total BNF | |
|  |  | Mean | Range  (Min.–Max.) | Mean | Range  (Min.–Max.) | Mean | Range  (Min.–Max.) | Mean | Range  (Min.–Max.) | Mean | Range  (Min.–Max.) | Mean | Range  (Min.–Max.) |
| ENF | 10.8 | 4.4 | 3.8–4.9 | 6.7 | 6.0–7.6 | 4.2 | 3.6–4.7 | 6.4 | 5.6–7.2 | 3.9 | 3.4–4.4 | 6.0 | 5.3–6.8 |
| EBF | 15.9 | 15.6 | 14.6–16.9 | 28.3 | 26.3–30.6 | 14.8 | 14.0–15.8 | 26.7 | 25.2–28.7 | 13.9 | 13.1–15.0 | 25.1 | 23.6–27.2 |
| DNF | 3.8 | 0.9 | 0.6–1.2 | 1.2 | 0.8–1.7 | 0.8 | 0.5–1.2 | 1.2 | 0.7–1.6 | 0.8 | 0.5–1.1 | 1.1 | 0.7–1.5 |
| DBF | 9.8 | 12.6 | 11.5–13.9 | 21.4 | 19.4–23.5 | 11.9 | 10.8–13.2 | 20.1 | 18.1–22.4 | 11.2 | 10.2–12.3 | 18.9 | 17.2–20.9 |
| Grass | 33.6 | 18.4 | 16.7–21.7 | 30.4 | 27.6–35.6 | 17.4 | 15.6–20.5 | 28.6 | 26.0–33.8 | 16.4 | 14.7–19.2 | 27.0 | 24.6–31.6 |
| Total | 81.0 | 51.9 | 49.4–56.0 | 87.9 | 83.2–94.2 | 49.0 | 46.5–53.4 | 83.0 | 78.2–89.8 | 46.1 | 44.0–49.9 | 78.1 | 73.8–84.0 |

**Table S9.** Comparison of biome-level symbiotic and total biological nitrogen fixation (BNF) estimated from three different products of gross primary production (GPP) (Bayesian approach). The three GPP sources are from MODIS [18], Keenan et al. [19], and Jung et al. [20]. In this table, the fraction of canopy uptake of N deposition to vegetation N demand (f_DEPd,tot_) is set as 0%.

| PFT | Area (10^6^ km^2^) | GPP from MODIS | | | |  | GPP from Jung | | | GPP from Keenan | | | |
| --- | --- | --- | --- | --- | --- | --- | --- | --- | --- | --- | --- | --- | --- |
|  |  | Symbiotic BNF | | Total BNF | | Symbiotic BNF | | Total BNF | | Symbiotic BNF | | Total BNF | |
|  |  | Mean | Median  (Min.–Max. of 95% CI) | Mean | Median  (Min.–Max. of 95% CI) | Mean | Median  (Min.–Max. of 95% CI) | Mean | Median  (Min.–Max. of 95% CI) | Mean | Median  (Min.–Max. of 95% CI) | Mean | Median  (Min.–Max. of 95% CI) |
| ENF | 10.8 | 4.7 | 4.7  (4.5–4.7) | 7.1 | 7.0  (6.9–7.1) | 4.0 | 4.0  (3.9–4.1) | 6.3 | 6.3  (6.2–6.4) | 5.2 | 5.3  (5.1–5.3) | 8.1 | 8.1  (7.9–8.1) |
| EBF | 15.9 | 16.4 | 16.4  (16.2–16.8) | 29.7 | 29.6  (29.1–30.0) | 18.1 | 18.0  (17.7–18.6) | 33.0 | 33.0  (32.3–34.1) | 15.7 | 15.5  (15.2–15.9) | 28.3 | 27.6  (28.0–28.9) |
| DNF | 3.8 | 1.2 | 1.3  (1.2–1.3) | 1.7 | 1.7  (1.6–1.8) | 0.6 | 0.6  (0.6–0.6) | 0.8 | 0.9  (0.8–0.9) | 0.9 | 0.9  (0.9–0.9) | 1.3 | 1.3  (1.3–1.3) |
| DBF | 9.8 | 12.2 | 12.2  (12.0–12.3) | 20.5 | 20.4  (20.1–20.7) | 13.1 | 13.1  (12.8–13.2) | 22.5 | 22.5  (21.9–22.9) | 14.8 | 14.9  (14.4–15.1) | 25.1 | 25.0  (24.4–25.4) |
| Grass | 33.6 | 17.9 | 17.9  (17.6–18.3) | 29.2 | 29.3  (28.9–29.8) | 17.7 | 17.7  (17.2–18.0) | 29.5 | 29.4  (29.0–30.1) | 23.0 | 22.9  (22.4–23.4) | 37.8 | 37.9  (37.1–38.7) |
| Total | 81.0 | 52.4 | 52.4  (51.5–53.4) | 88.2 | 88.1  (86.7–89.5) | 53.5 | 53.3  (52.3–54.5) | 92.1 | 92.1  (90.2–94.3) | 59.7 | 59.5  (58.1–60.7) | 100.6 | 99.9  (98.6–102.5) |

**Table S10.** Impacts of observational and prior uncertainties on estimates of total BNF (Tg N yr^-1^).

| PFT | Original estimates | Change in observation SD | | Change in prior SD | |
| --- | --- | --- | --- | --- | --- |
|  |  | -50%SD | +50%SD | -50%SD | +50SD |
| ENF | 7.2 | 7.5 | 6.9 | 6.3 | 7.4 |
| EBF | 25.2 | 24.3 | 25.7 | 23.6 | 25.4 |
| DNF | 1.2 | 1.3 | 1.0 | 0.9 | 1.2 |
| DBF | 22.4 | 21.7 | 22.4 | 20.3 | 22.6 |
| Other Forests | 0.0 | 0.0 | 0.0 | 0.0 | 0.0 |
| Grass | 33.8 | 32.6 | 33.9 | 30.4 | 34.4 |
| Total | 89.8 | 87.4 | 90.0 | 81.5 | 91.0 |

**Table S11.** Impacts of observational and prior uncertainties on estimates of total BNF (Tg N yr^-1^).

| PFT | Original estimates | Change in observation SD | | Change in prior SD | |
| --- | --- | --- | --- | --- | --- |
|  |  | -50%SD | +50%SD | -50%SD | +50SD |
| ENF | 4.7 | 4.9 | 4.4 | 4.1 | 4.8 |
| EBF | 14.0 | 13.5 | 14.3 | 13.1 | 14.1 |
| DNF | 0.8 | 1.0 | 0.7 | 0.7 | 0.9 |
| DBF | 13.2 | 12.9 | 13.2 | 12.0 | 13.4 |
| Other Forests | 0.0 | 0.0 | 0.0 | 0.0 | 0.0 |
| Grass | 20.5 | 19.9 | 20.6 | 18.5 | 21.0 |
| Total | 53.4 | 52.2 | 53.2 | 48.3 | 54.1 |

**Table S12.** Comparison of the isotope-based biological nitrogen fixation (BNF) (this study) with those simulated by Earth System Models (ESMs) in CMIP6 in different latitudinal bands (Unit: Tg N).

|  | 60°N~90°N | 30°N~60°N | 30°S~30°N | 90°S~30°S | Total |
| --- | --- | --- | --- | --- | --- |
| This study (GPP_MODIS_) | 4.6 | 15.7 | 56.1 | 1.7 | 78.2 |
| This study (GPP_Keenan_) | 2.6 | 18.9 | 65.6 | 2.6 | 89.8 |
| This study (GPP_Jung_) | 1.6 | 12.0 | 65.9 | 1.4 | 80.9 |
| ACCESS-ESM1-5 | 0.1 | 17.6 | 109.5 | 3.5 | 130.7 |
| CESM2 | 4.4 | 18.1 | 33.1 | 3.8 | 59.4 |
| CESM2-WACCM-FV2 | 3.7 | 20.8 | 37.8 | 4.1 | 66.5 |
| EC-Earth3-Veg | 1.1 | 4.6 | 23.9 | 1.3 | 30.9 |
| MIROC-ES2L | 4.5 | 26.4 | 40.1 | 2.6 | 73.7 |
| MPI-ESM-1-2-HAM | 6.1 | 20.3 | 54.9 | 1.8 | 83.2 |
| MPI-ESM1-2-LR | 6.2 | 21.1 | 51.5 | 1.4 | 80.2 |
| NorESM2-LM | 2.1 | 11.7 | 15.2 | 3.1 | 32.2 |
| NorESM2-MM | 2.5 | 10.6 | 16.7 | 3.1 | 33.0 |
| UKESM1-0-LL | 5.5 | 19.7 | 50.0 | 2.4 | 77.6 |
| TaiESM1 | 3.3 | 23.2 | 47.3 | 3.2 | 76.9 |

**Table S13.** Isotope fractionation factors for plant N uptake and isotope signatures of biological nitrogen fixation (BNF) reported in the literature

|  | Year | BNF | Uptake of NO_3_^-^ | Uptake of NH_4_^+^ |
| --- | --- | --- | --- | --- |
| Denk *et al.* [14] | 2017 | −2.02 ± 2.18‰  (median of −2.2‰) | −5.9 ± 3.7‰  (median −5.4‰) | −9.4 ± 6.6‰  (median −8.0‰) |
| Bai and Houlton [30] | 2009 | -2~0 ‰ |  |  |
| David Robinson [40] | 2001 | -6~0 ‰ | -19~0‰ | -18~-9‰ |
| Houlton et al. [2] | 2015 | 0 ‰ |  |  |
| Bai *et al.* [41] | 2012 | 0 ‰ |  |  |
| Craine *et al.* [12] | 2015 | -2.5 ‰ | -0.25~0‰ |  |

**Table S14** Summary of the fractions of N deposition retained by canopy and the fraction of plant N demands satisfied by canopy N uptake (f_DEPd,tot_) in the literature.

| Reference | Sites | Fraction of N deposition retained by canopy | Fraction of plant N requirements met by canopy N uptake | Species |
| --- | --- | --- | --- | --- |
| Lovett & Lindberg [42] | 12 sites in US |  | <15% | Table 1 in the reference |
| Boyce *et al.* [43] | 44°22′N, 73°54′W |  | 2-8% | Mature red spruce |
| Muller *et al.* [44] | laboratory |  | 10-15% | Norway spruce (Picea abies) |
| Wilson and Tiley [45] | / |  | 5% | Norway spruce |
| Ammann *et al.* [46] | 47°8′N, 7°35′E |  | 10–25% | P. abies |
| Schulze E. -D. [47] | Europe | 40–65% | 15–40% | European forest |
| Siegwolf *et al.* [48] | laboratory |  | 15–20% | Hybrid poplar (Populus euramericana) |
| Tomaszewski *et al.* [49] | 40°02′N, 105°33′′W |  | 10-15% | Engelmann spruce (Picea engelmannii Parry ex Engelm.), subalpine fir (Abies lasiocarpa (Hook.) Nutt.), and lodgepole pine (Pinus contorta Dougl. ex Loud.). |
| Vallano and Sparks [50] | laboratory |  | 11% and 15% | Nicotiana tabacum and L. esculentum |
| Sievering *et al.* [51] | 40◦2′N, 105◦33′W | 50-80% |  | Picea engelmannii Parry(Engelmann spruce),Abies lasiocarpa(Hook.) Nutt.(subalpinefir) andPinus contorta Dougl.(lodge-pole pine |
| Gaige *et al.* [52] | 45° 12′ N, 68° 45′ W | 70% |  | Red spruce (Picea rubens Sarg.) and eastern hemlock (Tsuga canadensis (L.) Carr.) |
| Wortman *et al.* [53] | 46°01′N, 8°50′E; 47°28′N, 8°21′E | 20-25% |  | Quercus cerris, Quercus pubescens, Castanea sativa, Betula pendula |
| Uscola *et al.* [54] | / |  | 10% | Quercus ilex and Pinus halepensis |
| Schwarz *et al.* [55] | 53°2′ N, 13°51′E; 51°10′ N, 10°23′E; 48°24′ N, 9°24′E | 16-51% |  | Spruce forests, Beech forests |
| Houle *et al.* [56] | 47°20′N, 71°08′W | 52-59% |  | Canadian boreal forest |
| Nair *et al.* [57] | 55°86′N, 3°20′W | 60% |  | Sitka spruce |
| Bourgeois *et al.* [58] | 45°02’N, 6°20’E |  | 4-16% | D. glomerata or F. paniculata |
| Liu *et al.* [59] | 31°46′–31°52′ N, 114°01′–114°06′ E | 44-52% |  | Mixed deciduous forest |
| Ferraretto *et al.* [60] | 56°36' N, 3°48'W | 70% |  | Sitka spruce |

**Table S15.** Plant functional type (PFT)-specific coefficients for converting leaf C:N ratio to those of wood and root in ORCHIDEE model [23].

| Plant functional types (PFTs) | Conversion coefficient | |
| --- | --- | --- |
|  | Wood | Root |
| Tropical broad-leaved evergreen | 0.108 | 0.86 |
| Tropical broad-leaved raingreen | 0.110 | 0.86 |
| Temperate needleleaf evergreen | 0.134 | 0.86 |
| Temperate broad-leaved evergreen | 0.128 | 0.86 |
| Temperate broad-leaved summergreen | 0.110 | 0.86 |
| Boreal needleleaf evergreen | 0.134 | 0.86 |
| Boreal broad-leaved summergreen | 0.110 | 0.86 |
| Boreal needleleaf summergreen | 0.077 | 0.86 |
| C3 grass | 1.000 | 0.86 |
| C3 pasture | 1.000 | 0.86 |
| C4 grass | 1.000 | 0.86 |
| C4 pasture | 1.000 | 0.86 |
| C3 agriculture | 1.000 | 0.86 |
| C4 agriculture | 1.000 | 0.86 |

**Table S16.** The Variance Inflation Factor (VIF) of the 10 predictors selected from Recursive Feature Elimination.

| Predictors | OC | AM | ECM | N-fixers | NOy | T | P/PET | P | Clay | NHx |
| --- | --- | --- | --- | --- | --- | --- | --- | --- | --- | --- |
| VIF | 1.21 | 29.04 | 34.14 | 4.79 | 1.72 | 7.25 | 6.66 | 13.22 | 1.62 | 1.58 |

**Table S17.** The Variance Inflation Factor (VIF) of the finally selected 8 predictors.

|  | OC | ECM | N-fixers | NOy | T | P/PET | Clay | NHx |
| --- | --- | --- | --- | --- | --- | --- | --- | --- |
| VIF | 1.20 | 3.33 | 2.41 | 1.67 | 4.43 | 1.52 | 1.60 | 1.51 |

**Table S18.** Rules for aggregating different plant functional types (PFTs) in this study.

| PFT in ORCHIDEE | Our aggregation | IGBP land cover type |
| --- | --- | --- |
| Temperate needleleaf evergreen | Evergreen needleleaf forest (ENF) | ENF |
| Boreal needleleaf evergreen |  |  |
| Tropical broad-leaved evergreen | Evergreen broadleaf forest (EBF) | EBF |
| Temperate broad-leaved evergreen |  |  |
| Boreal needleleaf summergreen | Deciduous needleleaf forest (DNF) | EBF |
| Tropical broad-leaved raingreen | Deciduous broadleaf forest (DBF) | DBF |
| Temperate broad-leaved summergreen |  |  |
| Boreal broad-leaved summergreen |  |  |
| C3 grass | Grass | Grasslands, savannas, and woody savannas |
| C4 grass |  |  |

**Table S19.** Comparison of isotope based biological nitrogen fixation (BNF) in unit area (this study) with those simulated by terrestrial ecosystem models (TEMs) and previous publications (Unit: Tg/M km^2^)

| PFT | Terrestrial Ecosystem Models | | Davies‐Barnard and Friedlingstein [31] | | Cleveland *et al.* [4]  (CASA-CNP) | | This study | |
| --- | --- | --- | --- | --- | --- | --- | --- | --- |
|  | GOLUM | ORCHIDEE | S-BNF | T-BNF | S-BNF | T-BNF | S-BNF | T-BNF |
| ENF | 0.49 | 1.43 | 0.38 | 0.51 | 0.05 | 0.13 | 0.39 | 0.59 |
| EBF | 2.51 | 1.42 | 0.51 | 0.94 | 2.34 | 3.04 | 0.93 | 1.68 |
| DNF | 0.09 | 0.34 | 0.38 | 0.51 | 0.05 | 0.57 | 0.21 | 0.32 |
| DBF | 0.61 | 0.71 | 0.83 | 0.90 | 1.96 | 2.23 | 0.70 | 1.19 |
| Grass | 0.46 | 0.48 | 0.64 | 0.97 | 2.00 | 2.17 | 0.52 | 0.85 |
| Total | 0.93 | 0.81 | 0.62 | 1.11 | 1.18 | 1.44 | 0.60 | 1.02 |

**Table S20.** Comparison of biome-level symbiotic and total biological nitrogen fixation (BNF) estimated from three different products of gross primary production (GPP) (Monte Carlo approach). The three GPP sources are from MODIS [18], Keenan et al. [19], and Jung et al. [20]. In this table, the fraction of canopy uptake of N deposition to vegetation N demand (f_DEPd,tot_) is set as 10%.

| PFT | Area (10^6^ km^2^) | GPP from MODIS | | | |  | GPP from Jung | | | GPP from Keenan | | | |
| --- | --- | --- | --- | --- | --- | --- | --- | --- | --- | --- | --- | --- | --- |
|  |  | Symbiotic BNF | | Total BNF | | Symbiotic BNF | | Total BNF | | Symbiotic BNF | | Total BNF | |
|  |  | Mean | Median  (Min.–Max. of 95% CI) | Mean | Median  (Min.–Max. of 95% CI) | Mean | Median  (Min.–Max. of 95% CI) | Mean | Median  (Min.–Max. of 95% CI) | Mean | Median  (Min.–Max. of 95% CI) | Mean | Median  (Min.–Max. of 95% CI) |
| ENF | 10.8 | 4.8 | 4.8  (4.7–5.0) | 6.7 | 6.8  (6.3–7.1) | 4.2 | 4.2  (3.9–4.5) | 6.0 | 5.9  (5.8–6.1) | 5.3 | 5.3  (5.0–5.5) | 7.5 | 7.4  (7.3–7.9) |
| EBF | 15.9 | 16.9 | 16.8  (15.3–18.8) | 27.0 | 27.0  (25.2–30.0) | 18.9 | 18.7  (17.6–19.8) | 30.3 | 30.1  (27.9–32.7) | 16.1 | 16.8  (15.7–17.0) | 25.7 | 26.0  (24.4–27.6) |
| DNF | 3.8 | 1.4 | 1.3  (1.3–1.4) | 1.8 | 1.7  (1.6–1.8) | 0.6 | 0.6  (0.6–0.6) | 0.8 | 0.9  (0.7–0.9) | 0.9 | 0.9  (0.9–0.9) | 1.2 | 1.3  (1.2–1.4) |
| DBF | 9.8 | 12.4 | 12.6  (11.6–13.0) | 18.5 | 18.8  (17.7–19.8) | 13.4 | 13.7  (12.6–14.2) | 20.4 | 20.7  (19.2–21.9) | 14.9 | 14.6  (14.0–15.7) | 22.5 | 22.8  (21.4–23.8) |
| Grass | 33.6 | 17.8 | 17.7  (17.3–18.4) | 26.2 | 26.6  (25.0–27.4) | 18.1 | 18.4  (17.1–19.1) | 27.2 | 27.6  (25.7–28.1) | 22.9 | 22.7  (21.7–24.2) | 34.0 | 34.1  (32.5–35.4) |
| Total | 81.0 | 53.3 | 53.2  (50.2–56.6) | 80.3 | 80.9  (75.9–86.2) | 55.1 | 55.6  (51.7–58.2) | 84.6 | 85.2  (79.2–89.6) | 60.1 | 60.3  (57.3–63.3) | 90.9 | 91.5  (86.8–96.0) |

**Table S21.** Comparison of biome-level symbiotic and total biological nitrogen fixation (BNF) estimated from three different products of gross primary production (GPP) (Monte Carlo approach). The three GPP sources are from MODIS [18], Keenan et al. [19], and Jung et al. [20]. In this table, the fraction of canopy uptake of N deposition to vegetation N demand (f_DEPd,tot_) is set as 0%.

| PFT | Area (10^6^ km^2^) | GPP from MODIS | | | |  | GPP from Jung | | | GPP from Keenan | | | |
| --- | --- | --- | --- | --- | --- | --- | --- | --- | --- | --- | --- | --- | --- |
|  |  | Symbiotic BNF | | Total BNF | | Symbiotic BNF | | Total BNF | | Symbiotic BNF | | Total BNF | |
|  |  | Mean | Median  (Min.–Max. of 95% CI) | Mean | Median  (Min.–Max. of 95% CI) | Mean | Median  (Min.–Max. of 95% CI) | Mean | Median  (Min.–Max. of 95% CI) | Mean | Median  (Min.–Max. of 95% CI) | Mean | Median  (Min.–Max. of 95% CI) |
| ENF | 10.8 | 5.7 | 5.6  (5.3–5.9) | 8.0 | 7.9  (7.5–8.5) | 4.9 | 5.0  (4.6–5.0) | 7.0 | 6.8  (6.7–7.5) | 6.2 | 6.2  (6.0–6.5) | 8.7 | 8.8  (8.2–9.3) |
| EBF | 15.9 | 19.1 | 19.0  (17.3–20.3) | 30.5 | 30.9  (28.6–32.2) | 21.3 | 21.5  (19.2–22.5) | 34.2 | 33.3  (31.7–36.0) | 18.1 | 17.6  (17.1–19.6) | 28.9 | 27.8  (28.0–30.4) |
| DNF | 3.8 | 1.6 | 1.6  (1.6–1.7) | 2.1 | 1.9  (1.9–2.3) | 0.7 | 0.7  (0.7–0.7) | 0.9 | 0.9  (0.9–1.0) | 1.1 | 1.1  (1.1–1.1) | 1.5 | 1.4  (1.4–1.4) |
| DBF | 9.8 | 14.2 | 14.0  (13.6–14.9) | 21.2 | 21.3  (20.5–22.1) | 15.2 | 15.3  (14.6–15.9) | 23.2 | 23.4  (21.9–24.7) | 17.0 | 17.3  (16.1–18.0) | 25.6 | 25.6  (23.9–27.1) |
| Grass | 33.6 | 20.3 | 20.1  (19.3–21.2) | 29.9 | 30.1  (28.5–31.3) | 20.5 | 20.0  (19.6–21.5) | 30.8 | 30.9  (29.6–32.2) | 26.0 | 26.0  (24.7–27.4) | 38.7 | 38.3  (36.3–41.1) |
| Total | 81.0 | 61.0 | 60.4  (57.1–64.1) | 91.7 | 92.2  (87.2–96.4) | 62.7 | 62.5  (58.7–65.6) | 96.1 | 95.4  (90.9–101.3) | 68.4 | 68.2  (64.9–72.7) | 103.3 | 101.8  (97.9–109.4) |
